# *Perilla frutescens* Leaf-Derived Extracellular Vesicle-Like Particles Carry Pab-miR-396a-5p to Alleviate Psoriasis by Modulating IL-17 Signaling

**DOI:** 10.34133/research.0675

**Published:** 2025-04-17

**Authors:** Yali Liu, Shanmin Tao, Zhengwei Zhang, Tianjiao Li, Haoran Wang, Jiankang Mu, Yunke Wu, Ziheng He, Cheng Zhang, Dominique Jasmin Lunter, Peng Cao

**Affiliations:** ^1^ State Key Laboratory of Technologies for Chinese Medicine Pharmaceutical Process Control and Intelligent Manufacture, Nanjing University of Chinese Medicine, Nanjing 210023, China.; ^2^ Jiangsu Provincial Medical Innovation Center, Affiliated Hospital of Integrated Traditional Chinese and Western Medicine, Nanjing University of Chinese Medicine, Nanjing 210028, China.; ^3^ Shandong Academy of Chinese Medicine, Jinan 250014, China.; ^4^ Department of Pharmaceutical Technology, Faculty of Science, Eberhard Karls Universität Tübingen, 72076 Tuebingen, Germany.

## Abstract

Psoriasis, a chronic inflammatory skin disorder, remains challenging to treat due to poor skin barrier penetration, limited efficacy, and adverse effects of current therapies. Natural plant-derived extracellular vesicle-like particles (EVPs) have emerged as biocompatible carriers for bioactive molecules. Among various medicinal plants screened, *Perilla frutescens* leaf-derived EVPs (PLEVPs) exhibited strong anti-inflammatory and antioxidant effects. By incorporating PLEVPs into a hydrogel formulation, we enhanced their stability, retention at psoriatic lesions, and transdermal delivery efficiency. In vivo studies demonstrated that the PLEVPs markedly alleviated psoriasis symptoms in both preventive and therapeutic mouse models, outperforming conventional treatments. This effect was attributed to reduced oxidative stress, modulation of Treg cells, and promotion of keratinocyte apoptosis. Transcriptomic analysis revealed enrichment of the interleukin-17 (IL-17) signaling pathway, a major driver of psoriasis, while small RNA sequencing identified pab-miR396a-5p, an endogenous microRNA (miRNA) within PLEVPs, as a key regulator. Mechanistic studies showed that pab-miR396a-5p targets the 3′-untranslated region of plant heat shock protein 83a, a homolog of mammalian heat shock protein 90, leading to the suppression of nuclear factor-kappa B and Janus kinase/signal transducers and activators of transcription signaling, inhibiting the IL-17 signaling pathway. Validation using lipid nanoparticles encapsulating pab-miR396a-5p mimics confirmed comparable therapeutic effects. This study highlights the potential of plant-derived EVPs as carriers of endogenous miRNAs, enabling interkingdom communication and offering a scalable platform for psoriasis therapy.

## Introduction

Psoriasis is a chronic inflammatory skin disease characterized by recurrent, erythematous plaques with a high prevalence worldwide [1]. It involves abnormal proliferation of keratinocytes, as well as immune dysregulation that primarily affects the epidermis and dermis, resulting in the characteristic thickening of the skin [2]. Traditional therapies, such as corticosteroids, vitamin D analogs, and systemic immunosuppressants like cyclosporine, provide symptomatic relief but are associated with adverse effects, including skin irritation, immune suppression, and increased susceptibility to infections [3–5]. Similarly, traditional Chinese medicine (TCM) formulations, though widely used for their multi-targeted approach, often face limitations such as slow therapeutic effects and inconsistent efficacy [6]. Recently, new biologics that target specific inflammatory pathways such as interleukin-17 (IL-17) have shown promise, yet high costs and potential side effects limit their accessibility and long-term use. These challenges emphasize the urgent need for safer and more effective alternatives in psoriasis treatments [7,8].

Extracellular vesicles (EVs) and particles have recently gained prominence as natural and biocompatible carriers, showing potential to address limitations in current psoriasis treatments [9,10]. They are nanosized vesicles with a phospholipid bilayer structure that facilitate intercellular communication by transporting bioactive molecules such as proteins, lipids, and nucleic acids [11,12]. Mammalian-derived EVs, particularly those from sources like stem cells, have shown therapeutic potential for tissue repair and immune modulation but are limited by high production costs and low yields, which constrain their clinical applications [13–15]. In contrast, plant-derived EV-like particles (EVPs) present unique advantages, including high availability, lower immunogenic risk, and scalability for production [16,17]. These EVPs are also environmentally friendly and demonstrate strong biocompatibility, enabling therapeutic applications [18]. Recent studies have highlighted EVPs from plants like tea leaf, turmeric, and garlic of effectively delivering bioactive compounds and exhibiting anti-inflammatory, antioxidant, and immune-modulatory effects [19–22]. These findings suggest that plant-derived EVPs might be promising candidates for psoriasis treatment, potentially addressing a gap in current research on these EVPs in dermatological applications.

MicroRNAs (miRNAs) are small, single-stranded endogenous noncoding RNAs that play essential roles in regulating a wide range of biological processes, including cell proliferation, cell cycle regulation, differentiation, and inflammation development [23,24]. Encapsulation of miRNAs within EVs has been shown to enhance their stability, bioavailability, and cellular uptake, as EVs provide a protective barrier against enzymatic degradation [25–27]. This natural delivery system has gained attention for its therapeutic potential, with recent studies highlighting plant-derived EVPs naturally carrying miRNAs to exert notable regulatory effects on mammalian cellular functions. This efficacy has been demonstrated in managing inflammatory conditions such as colitis, as well as in promoting wound healing in diabetic foot ulcers and suppressing tumor growth in cancer models [28–30]. Together with the success of nucleic acid-based therapies and approaches in psoriasis treatment [31,32], we hypothesize that the therapeutic effects of plant-derived EVPs may partly rely on their miRNA content. By examining the specific miRNAs carried by EVPs, this study aims to explore their potential regulatory roles in modulating key pathways associated with psoriasis, providing a foundation for understanding how plant-derived EVPs could serve as a natural therapeutic approach for inflammatory skin disorders.

**Fig. 1.**
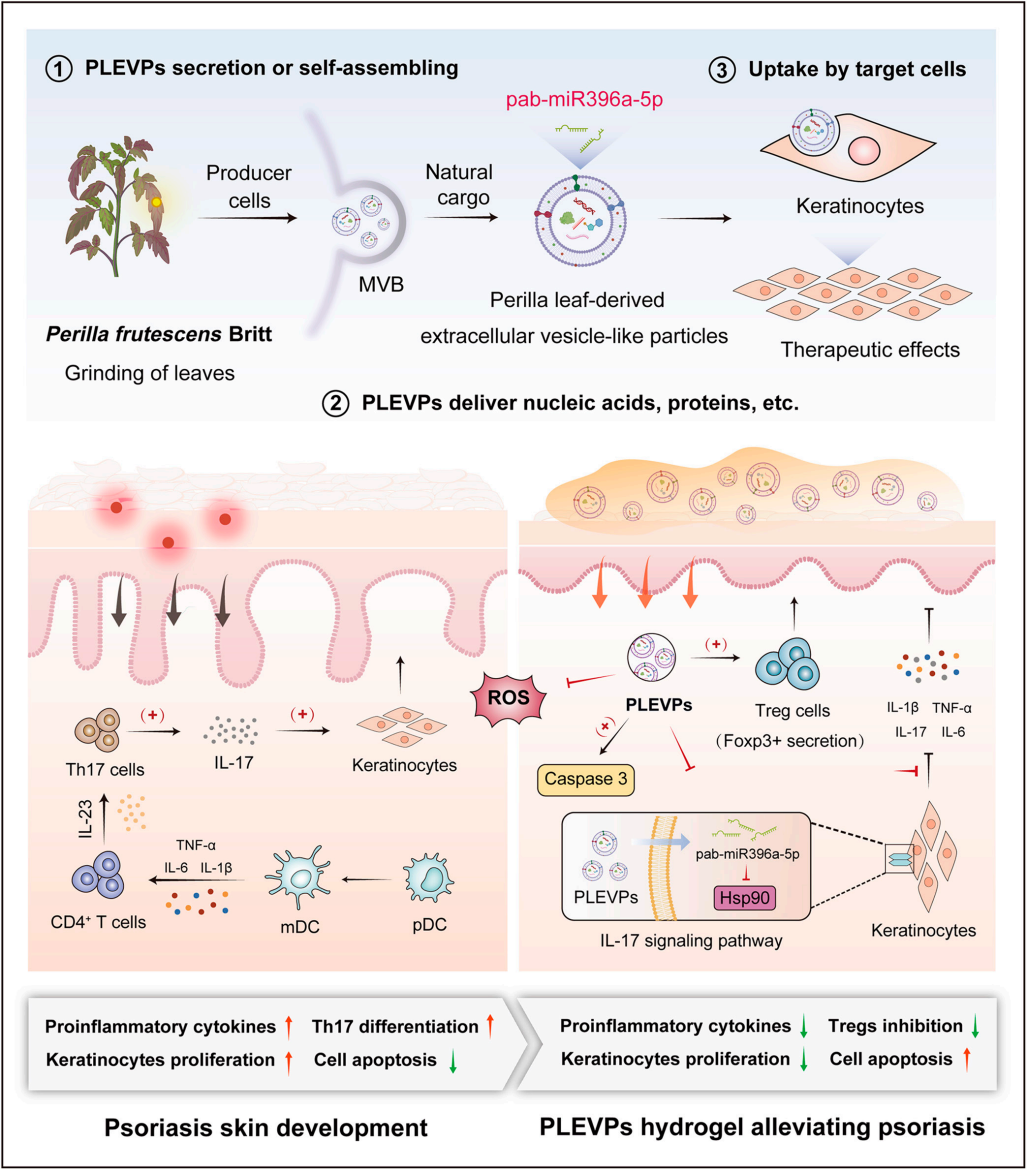
PLEVPs hydrogel alleviates psoriasis by modulating the IL-17 signaling pathway. PLEVPs are secreted or self-assembled from multivesicular bodies (MVBs) within *P. frutescens* leaf cells (①), delivering bioactive molecules including nucleic acids and proteins, with pab-miR396a-5p specially identified (②) and can be internalized by keratinocytes after topical application (③). Psoriasis progression is characterized by increased Th17 cell activation and IL-17 secretion, leading to keratinocyte hyperproliferation, inflammation, and apoptosis. The application of PLEVPs hydrogel leads to therapeutic effects of reducing reactive oxygen species (ROS), promoting apoptosis via caspase-3 activation, and increasing Treg (Foxp3^+^) cell activity. The pab-miR396a-5p delivered by PLEVPs targets HSP90, down-regulating the IL-17 signaling pathway and subsequently reducing pro-inflammatory cytokines (IL-17, TNF-α, and IL-6), inhibiting keratinocyte hyperproliferation and alleviating psoriasis symptoms.

Thus, in this study, we initially screen various medicinal plant-derived EVPs for their anti-inflammatory effects and identify promising candidate. To improve the stability and applicability of plant-derived EVPs in topical administration, a hydrogel system was further designed, which enhances their retention on the skin and facilitates prolonged drug release and improves therapeutic efficacy. Then, we aim to identify key miRNA components within PLEVPs and elucidate their regulatory impact on inflammatory signaling pathways associated with psoriasis through transcriptomic and small RNA analyses. By focusing on the molecular interactions and therapeutic efficacy, this study seeks to advance the understanding of plant-derived EVPs as natural and biocompatible therapeutic agents. Ultimately, our findings (Fig. 1) may offer a novel, plant-based approach to managing inflammatory skin disorders, expanding the possibilities for cross-kingdom therapeutics in clinical applications.

## Results

### PLEVPs exhibit potent antioxidant and anti-inflammatory effects in HaCaT cell models

**Fig. 2.**
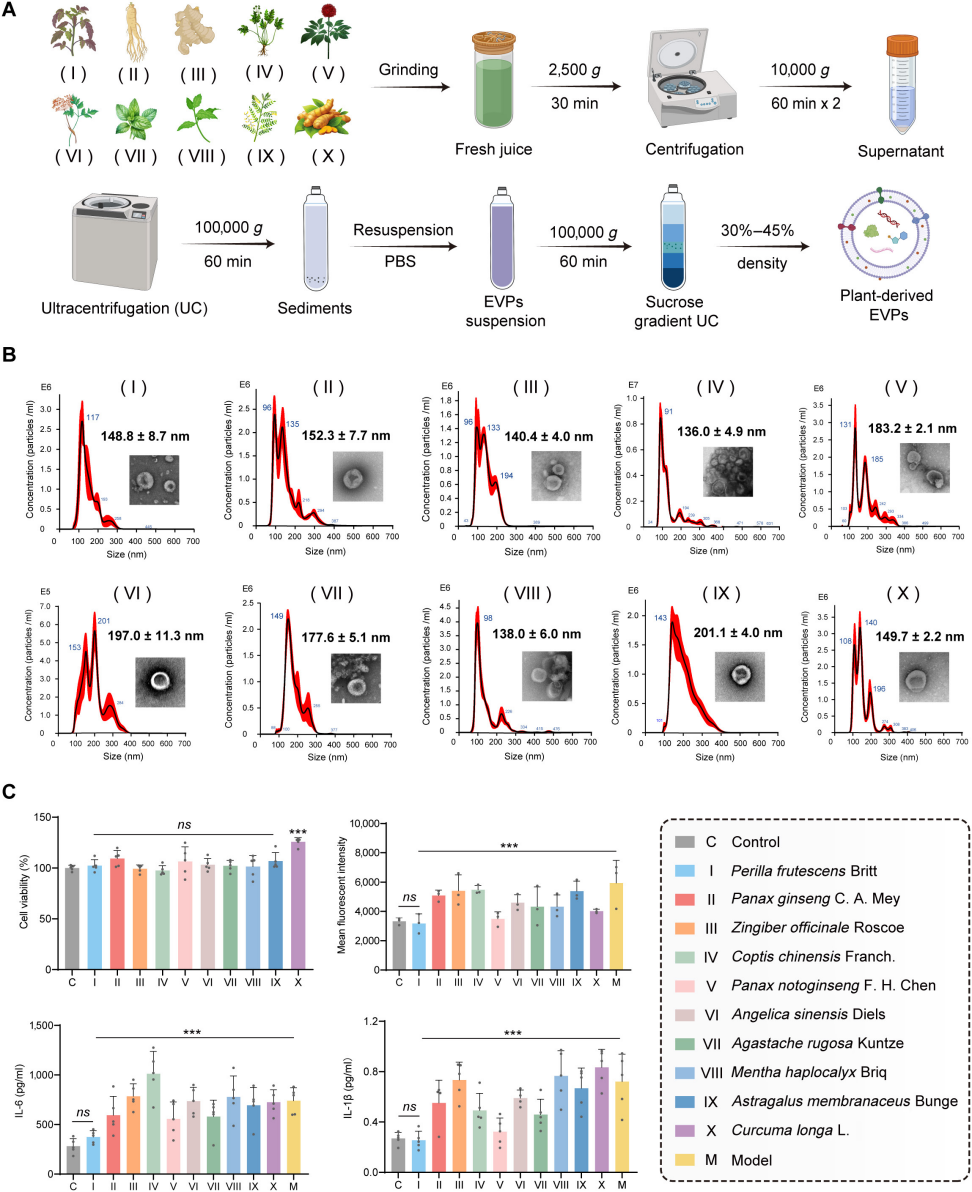
Extracellular vesicle-like particles (EVPs) from 10 different medicinal plants are successfully prepared and PLEVPs excel in reducing ROS and inflammatory cytokine levels in HaCaT cells. (A) Schematic of the preparation process, including grinding, centrifugation, ultracentrifugation, and sucrose gradient separation to obtain EVPs from different plants. (B) Particle size distribution and morphological images of 10 different plant-derived EVPs analyzed by NTA and TEM, respectively. (C) Effects of plant-derived EVPs on HaCaT cell viability, ROS production, and inflammatory cytokine secretion (IL-6 and IL-1β). Flow cytometry analysis was used to assess intracellular ROS levels. The corresponding plant sources are as follows: (I) *P. frutescens* Britt, (II) *Panax ginseng* C. A. Mey, (III) *Zingiber officinale* Roscoe, (IV) *Coptis chinensis* Franch., (V) *Panax notoginseng* F. H. Chen, (VI) *Angelica sinensis* Diels, (VII) *Agastache rugosa* Kuntze, (VIII) *Mentha haplocalyx* Briq, (IX) *Astragalus membranaceus* Bunge, and (X) *Curcuma longa* L. Data are presented as mean ± SD. Statistical analysis was performed using one-way ANOVA, with ***P < 0.001 indicating statistical significance. ns, no significance.

To identify EVPs with potential therapeutic efficacy in psoriasis treatment, we selected 10 medicinal plants based on their documented anti-inflammatory and antioxidant properties in the literature, as well as their frequent use in TCM formulations for treating inflammatory conditions [33]. Combining the process of differential centrifugation and sucrose density gradient ultracentrifugation, we successfully isolated EVPs from each plant (Fig. 2A), with their suspensions shown in Fig. S1. Transmission electron microscopy (TEM) confirmed their characteristic cup-shaped morphology, and nanoparticle tracking analysis (NTA) indicated their particle sizes ranging from 130 to 200 nm, consistent with previous studies on plant-derived EVPs (Fig. 2B).

To ensure the safety of these EVPs, we evaluated their cytotoxicity in HaCaT cells. As shown in Fig. 2C, all EVPs exhibited no significant cytotoxic effects, indicating their biocompatibility for further biological testing. We next evaluated the antioxidant and anti-inflammatory activities of these EVPs using IL-6-stimulated HaCaT cells as an in vitro inflammation model [34]. After treatment with them, ROS levels and inflammatory cytokine secretion in cell supernatants were determined. Among the 10 tested EVPs, PLEVPs notably reduced the ROS levels (Fig. S2) and pro-inflammatory cytokines, such as IL-6 and IL-1β, compared to the model group (Fig. 2C) and other EVPs, indicating superior antioxidant and anti-inflammatory effects. Thus, this initial screening suggests that PLEVPs have superior bioactive properties compared to the other EVPs, making them a promising candidate for further investigation in treating inflammatory skin diseases of psoriasis.

### Distinct composition and cellular effects of PLEVPs highlight their therapeutic potential

**Fig. 3.**
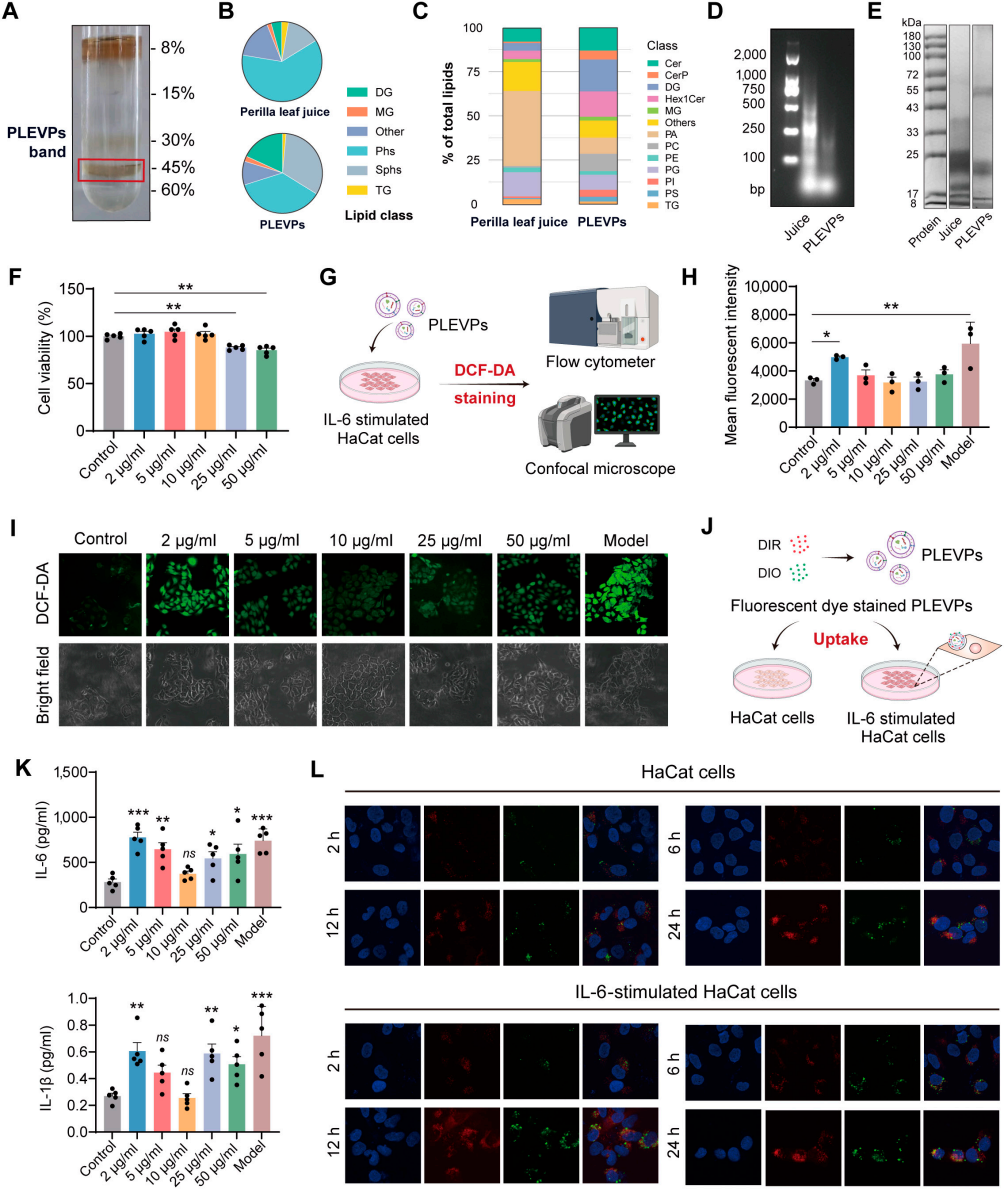
PLEVPs exhibit distinct profiles compared to *Perilla* leaf juice and inhibit oxidative stress and reduce pro-inflammatory cytokine secretion in HaCaT cells. (A) PLEVPs isolated from *Perilla* leaf juice using ultracentrifugation with a sucrose density gradient (8%, 15%, 30%, 45%, and 60%) and collected from the 45% interface. (B) Lipidomic analysis of major lipid categories comparing *Perilla* leaf juice and PLEVPs. (C) Detailed lipid subclass distribution in *Perilla* leaf juice and PLEVPs. (D) Protein patterns of *Perilla* leaf juice and PLEVPs analyzed by SDS-PAGE. (E) RNA profile of *Perilla* leaf juice and PLEVPs analyzed by agarose gel electrophoresis. (F) Cell viability of HaCaT cells treated with various concentrations of PLEVPs (2, 5, 10, 25, and 50 μg/ml) for 48 h (*n* = 5). (G) Schematic of PLEVPs treatment in IL-6-stimulated HaCaT cells followed by DCF-DA staining to assess intracellular ROS levels using flow cytometry and confocal microscopy. (H) Mean fluorescent intensity (MFI) of ROS levels in IL-6-stimulated HaCaT cells after PLEVPs treatment (2 to 50 μg/ml) (*n* = 3). (I) Representative DCF-DA fluorescence images for ROS levels in HaCaT cells. (J) ELISA quantification of IL-6 and IL-1β levels in the supernatants of IL-6-stimulated HaCaT cells following PLEVPs treatment (*n* = 5). (K) Schematic procedures of cellular uptake studies using DIR- and DIO-stained PLEVPs in both HaCaT cells and IL-6-stimulated HaCaT cells. (L) Representative confocal images of the uptakes of PLEVPs labeled with DIR (red) and DIO (green) by HaCaT cells and IL-6-stimulated HaCaT cells at different time points (2, 6, 12, and 24 h). Data are presented as mean ± SD. Statistical analysis was performed using one-way ANOVA, with *P < 0.05, **P < 0.01, ***P < 0.001 indicating statistical significance.

To further investigate the molecular basis of these bioactivities, we performed comprehensive analysis of PLEVPs and specially focused on their RNA, protein, lipid, and metabolite compositions compared with *Perilla* leaf juice. First, PLEVPs were successfully isolated from the 45% sucrose density layer with high purity and size distribution (Fig. 3A and Fig. S3). In lipidomic analysis, although both samples shared similar lipid classes, PLEVPs were particularly enriched in diacylglycerol (DG) and sphingolipids (Sphs) (Fig. 3B). Detailed analysis also revealed a notable enrichment of ceramides and hexosylceramides in PLEVPs (Fig. 3C), which are important for maintaining the skin barrier and modulating immune responses [35]. We then performed agarose gel electrophoresis to compare RNA profiles between *Perilla* leaf juice and PLEVPs. It shows that the juice contained a wider range of RNA fragments, including longer sequences, whereas PLEVPs were predominantly enriched in smaller RNA species (Fig. 3D). Protein profiles obtained from sodium dodecyl sulfate–polyacrylamide gel electrophoresis (SDS-PAGE) were also found to be markedly different. The juice exhibited a complex array of proteins, whereas PLEVPs contained a narrower range of proteins, mainly below 72 kDa (Fig. 3E). Relatively good consistency was also demonstrated by comparing metabolomic profiles and protein bands from different batches (Figs. S5 and S6). We also found in metabolomic analysis that PLEVPs contained a higher proportion of flavonoids and other secondary metabolites compared to *Perilla* leaf juice (Fig. S4). These compounds are known for their anti-inflammatory, antioxidant, and antibacterial effects, which likely contribute to their superior bioactivity. According to these findings, the enriched bioactive components in PLEVPs form the basis for their biological activity and contribute to their therapeutic potential.

Further, different concentrations of PLEVPs ranging from 2 to 50 μg/ml were tested to determine their optimal dose. The cell viability assay indicated that concentrations from 2 to 10 μg/ml had minimal impact on cell viability, while a slight reduction was observed at higher concentrations of 25 and 50 μg/ml (Fig. 3F). To evaluate the inhibitory effects of PLEVPs on keratinocyte proliferation, we employed an IL-6-induced proliferation model, as IL-6 is a well-established cytokine widely used to stimulate keratinocyte hyperproliferation in inflammatory conditions. As shown in Fig. S7, PLEVPs treatment (2, 5, 10, 25, and 50 μg/ml) significantly suppressed IL-6-induced proliferation. Notably, the inhibitory effect was most pronounced at 10 and 25 μg/ml, with significant reductions observed at 48 and 72 h, suggesting a sustained anti-proliferative effect of PLEVPs. Interestingly, when analyzing ROS levels, we observed a significant reduction in ROS across all tested concentrations (5 to 50 μg/ml), with the most notable decrease seen at 10 and 25 μg/ml based on flow cytometry data (Fig. 3G and H and Fig. S2). Confocal microscopy results further supported these findings, showing that the fluorescence intensity decreased consistently with PLEVPs treatment, with 10 μg/ml resulting in the lowest fluorescence signal, indicating the most effective reduction in ROS accumulation (Fig. 3I). In parallel, enzyme-linked immunosorbent assay (ELISA) results indicated different extent of the anti-inflammatory effects of PLEVPs. The 10 μg/ml concentration of PLEVPs led to the most significant decrease in cytokine levels, while both lower (2 μg/ml) and higher (50 μg/ml) doses exhibited reduced efficacy (Fig. 3K). This suggests an optimal anti-inflammatory concentration at 10 μg/ml, generally consistent with the ROS reduction results. Confocal microscopy further demonstrated that PLEVPs were efficiently internalized (Fig. 3J) by both HaCaT and IL-6-stimulated HaCaT cells in a time-dependent manner, with cellular uptake increasing over time (2, 6, 12, and 24 h, Fig. 3L). In this regard, 2 fluorescent dyes of DIR (red) and DIO (green) were used to label PLEVPs due to their differing properties. The internalization of both dyes implied that they had potential to deliver hydrophobic and hydrophilic bioactive substances.

### PLEVPs hydrogel exhibits improved stability, prolonged skin retention, and superior transdermal release properties

**Fig. 4.**
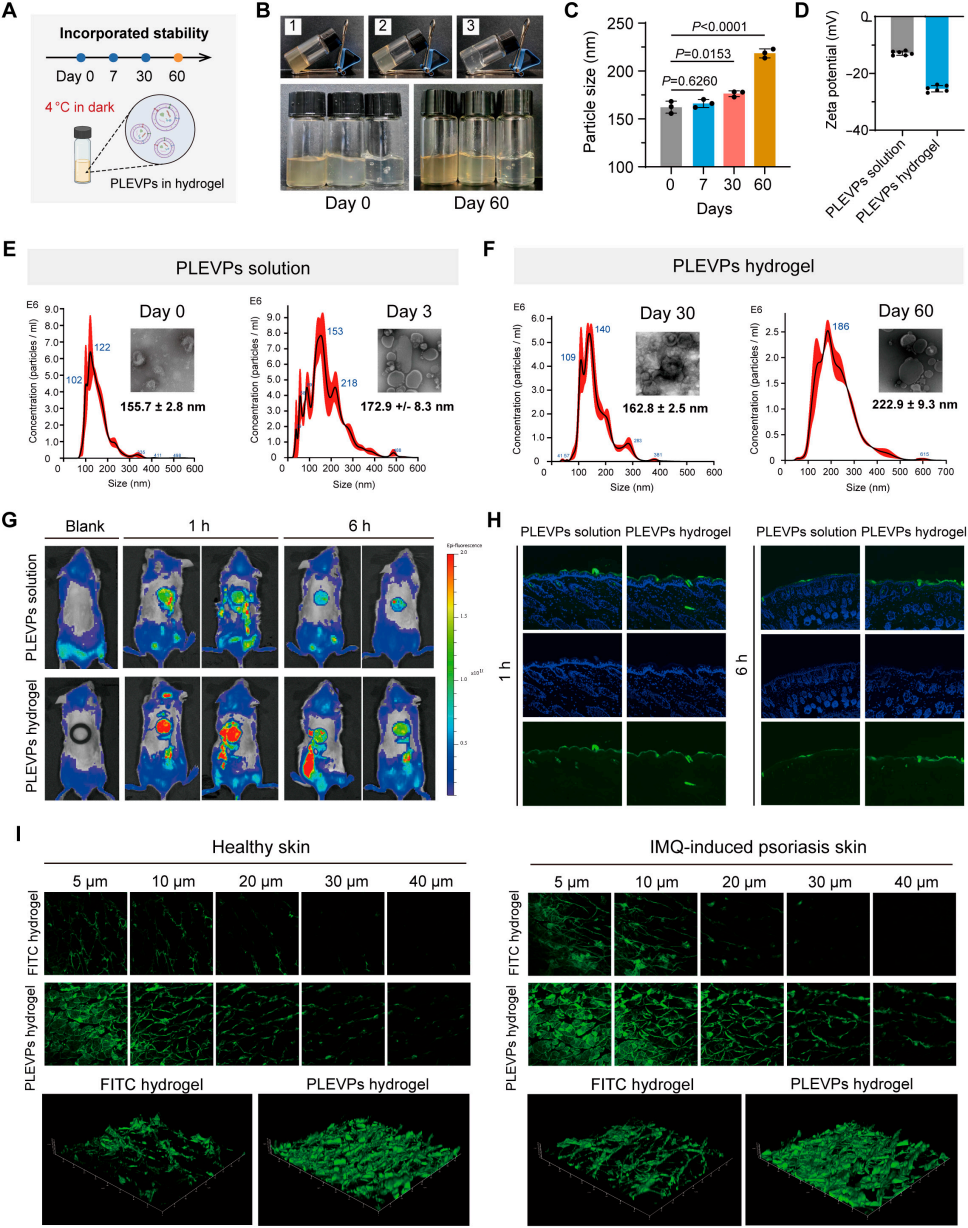
Stability characterization, retention, and release properties of PLEVPs incorporated into hydrogel formulations. (A) Schematic representation of the stability assay, with PLEVPs hydrogel stored at 4 °C under dark conditions and analyzed on days 0, 7, 30, and 60. (B) Representative images of Carbopol hydrogels containing PLEVPs at different concentrations 1, 0.5, and 0.2 mg/ml on day 0 and day 60. (C) Time-dependent changes in PLEVPs particle size in the hydrogel system, as determined by nanoparticle tracking analysis (NTA). (D) Zeta potential analysis comparing the surface charge stability of PLEVPs in solution versus hydrogel formulations (*n* = 6). (E and F) Particle size distribution and morphology of PLEVPs in solution and hydrogel formulations characterized by NTA and transmission electron microscopy (TEM). (G) In vivo fluorescence imaging of mouse skin showing enhanced retention of PLEVPs delivered via hydrogel compared to solution at 1 h and 6 h postapplication. (H) Cryosection images comparing PLEVPs release from solution and hydrogel in mouse skin. (I) Two-photon microscopy images showing transdermal penetration and distribution of PLEVPs hydrogel versus FITC hydrogel in both healthy and psoriatic mouse skin.

To enable effective topical application of PLEVPs, we developed a Carbopol hydrogel-based drug delivery system and systematically evaluated its effects on the stability, retention, and transdermal delivery of PLEVPs. As shown in Fig. S8, the PLEVPs hydrogel exhibited good adhesive properties, ensuring prolonged retention at the application site. Rheological analysis (Fig. S9) of the PLEVPs hydrogel further reveals its shear-thinning behavior and stable mechanical properties. The hydrogel maintains its viscosity and elasticity over time, confirming its stability under the tested conditions. Additionally, the in vitro release study (Fig. S10) indicated that the hydrogel provided sustained drug release over 72 h, in contrast to the PLEVPs solution, which exhibited a faster initial release. Stability tests of PLEVPs were conducted at 4 °C under dark conditions on days 0, 7, 30, and 60, comparing PLEVPs in solution and hydrogel formulations (Fig. 4A). Visual observations revealed that PLEVPs-loaded hydrogels at concentrations of 0.2, 0.5, and 1 mg/ml maintained a consistent appearance without sedimentation or phase separation over 60 days (Fig. 4B). NTA demonstrated stable particle sizes for PLEVPs in hydrogels over 30 days, with moderate increases observed by day 60, indicating that the hydrogel matrix effectively mitigated aggregation (Fig. 4C). In contrast, PLEVPs in solution exhibited significant particle size increases from 155.7 ± 2.8 nm on day 0 to 172.9 ± 8.3 nm on day 3, along with aggregation (Fig. 4E and F). Furthermore, zeta potential measurements confirmed that the hydrogel provided a stabilizing effect on the surface charge of PLEVPs (Fig. 4D), while TEM imaging reaffirmed the structural integrity of PLEVPs within the hydrogel matrix.

To assess the skin retention and distribution of PLEVPs, in vivo fluorescence imaging showed substantially stronger and longer-lasting skin retention of PLEVPs in the hydrogel group compared to the solution group. At 1 and 6 h, the fluorescence intensity in the hydrogel group remained much higher, indicating improved skin retention and prolonged drug action (Fig. 4G). Skin cryosection fluorescence imaging further confirmed the enhanced skin distribution of PLEVPs in the hydrogel group. At 1 h, PLEVPs from the hydrogel were observed in both the epidermis and dermis, with higher retention even at 6 h, while the solution group showed largely reduced distribution over time (Fig. 4H). To evaluate the transdermal delivery efficiency, 2-photon microscopy was employed using fluorescein isothiocyanate (FITC)-labeled PLEVPs hydrogel applied to both healthy and Imiquimod (IMQ)-induced psoriatic skin (Fig. 4I). In healthy skin, the hydrogel system demonstrated improved penetration compared to free FITC gel, although the outermost skin barrier limited the penetration depth. In psoriatic skin with compromised barrier integrity, PLEVPs showed obviously deeper and more extensive penetration, confirming their enhanced delivery potential. This superior transdermal delivery can be attributed to the phospholipid bilayer structure and nanoscale size of PLEVPs, which are consistent with previous findings on lipid-based nanocarriers overcoming skin barriers.

In summary, our study demonstrates that incorporation of PLEVPs into a hydrogel matrix highly enhances their stability during storage, prolongs their retention in skin tissues, and maintains superior transdermal delivery efficiency.

### Topically applied PLEVPs hydrogel effectively alleviates IMQ-induced psoriasis

**Fig. 5.**
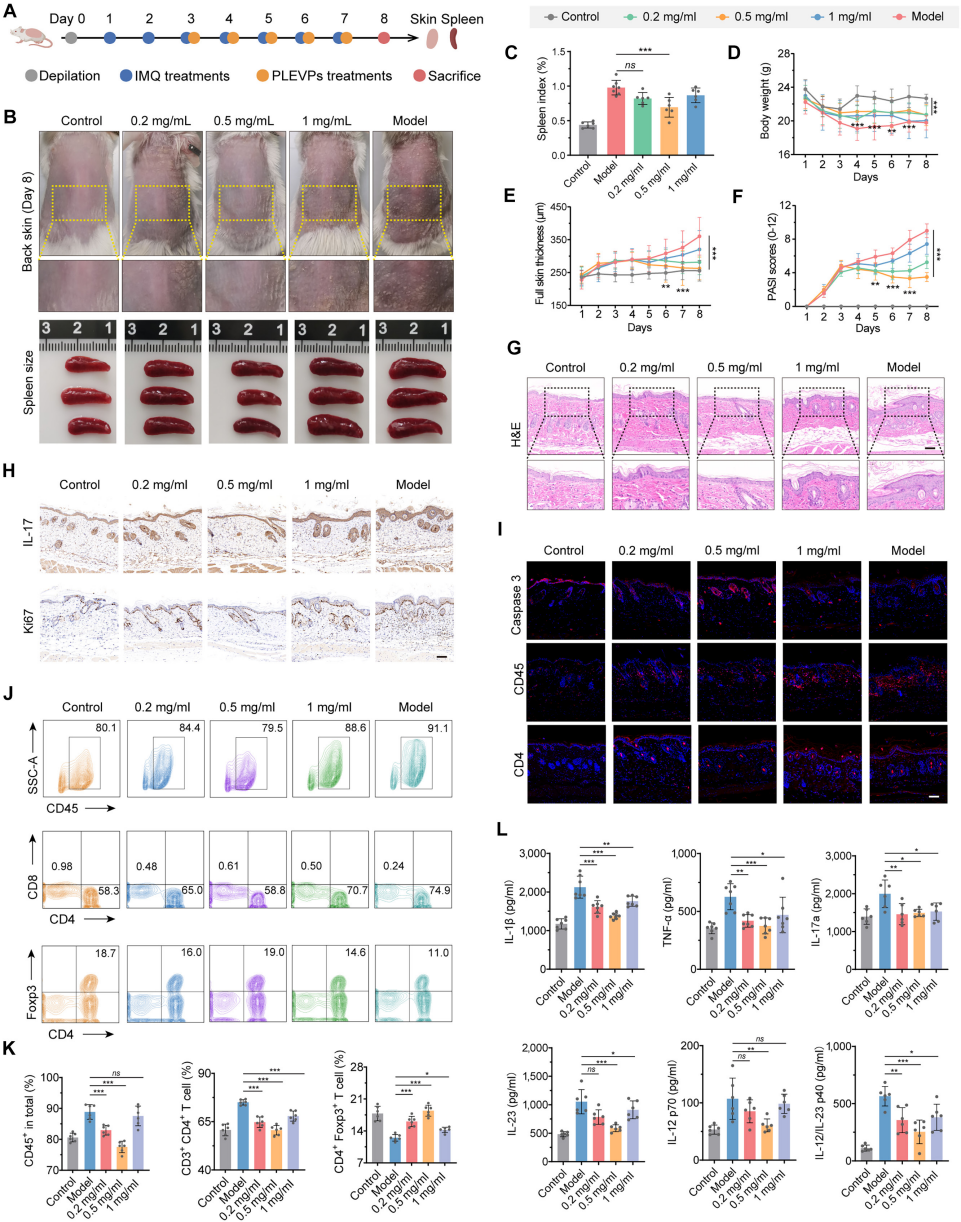
Topical administration of PLEVPs hydrogel demonstrate prevention efficacy in a psoriasis-like mouse model. (A) Experimental timeline covering the induction of psoriasis-like symptoms and the therapeutic administration of PLEVPs hydrogel. (B) Representative images of dorsal skin condition and spleen size in different treatment groups. (C) Spleen index comparison across different groups. (D) Skin thickness, (E) PASI scores, and (F) body weight profiles over course of treatments. The labels for the different treatment groups are shown in the gray box at the top of the figures. (G) H&E staining of sections with observation of epidermal thickness and inflammatory conditions. Scale bar, 100 μm. (H) Immunohistochemistry (IHC) analysis of IL-17 and Ki67 to assess inflammatory response and cell proliferation in the skin. Scale bar, 100 μm. (I) Immunofluorescence (IF) staining for Caspase-3, CD45, and CD4 in skin sections to assess apoptosis and immune cell infiltration. Scale bar, 100 μm. (J and K) Flow cytometry analysis of spleen single-cell suspensions, quantifying the proportions of CD8^+^ T cells, CD4^+^ T cells, and CD4^+^ Foxp3^+^ Treg cells. (L) ELISA quantification of inflammatory cytokines including IL-1β, TNF-α, IL-17a, IL-23p19, IL-12p70, and IL-12/23p40. Data are presented as mean ± SD (*n* = 6). Statistical analysis was performed using one-way ANOVA, with *P < 0.05, **P < 0.01, ***P < 0.001 indicating statistical significance.

To evaluate the therapeutic efficacy in alleviating IMQ-induced psoriasis, different concentrations (0.2, 0.5, and 1 mg/ml) of PLEVPs hydrogel were applied on BALB/c mice over 5 consecutive days (Fig. 5A). Representative images of dorsal skin and spleen size (Fig. S11 and Fig. 5B) indicated that mice in the model group developed severe psoriasis-like symptoms, including thickened, erythematous skin and splenomegaly. In contrast, PLEVPs treatment improved skin appearance and reduced spleen enlargement, with 0.5 mg/ml dose showing the most prominent therapeutic effect. Similar findings were reconfirmed in terms of the spleen index (Fig. 5C), who has been significantly reduced by the 0.5 mg/ml group compared with the model mice. Notably, from the first day of treatment, the PLEVPs-treated groups showed obvious improvement in body weight compared to the model group (Fig. 5D), although there was no significant dose-dependent difference. The therapeutic effect became more apparent when assessing skin thickness (Fig. 5E) and the Psoriasis Area and Severity Index (PASI) scores (Fig. 5F). Both parameters showed a noticeable reduction starting from the day of PLEVPs administration, with the 0.5 mg/ml dose being the most effective concentration, demonstrating its superior therapeutic potential. Histological analysis of skin tissue (Fig. 5G) further confirmed these findings, showing that PLEVPs treatment successfully reduced epidermal hyperplasia and thickening, especially at the 0.5 mg/ml dose. Subsequently, skin sections proceeded with immunohistochemical (IHC) staining (Fig. S12) for IL-17 and Ki67 (Fig. 5H), revealing a marked decrease in inflammation and keratinocyte proliferation in the PLEVPs-treated groups. Immunofluorescence (IF) analysis (Fig. S12) of key markers such as Caspase-3, CD45, and CD4 (Fig. 5I) showed increased keratinocyte apoptosis and reduced immune cell infiltration following treatment of PLEVPs hydrogel. Flow cytometry (Fig. 5J and K and Figs. S13 to S16) further supported these findings by showing a reduction in the proportion of CD45^+^ and CD3^+^CD4^+^ T cells, along with the restoration of CD4^+^Foxp3^+^ regulatory T cells in spleens of PLEVPs-treated mice, representing their restoration of immune balance. This modulation of Treg cells may be attributed to the potential contributions from bioactive compounds within PLEVPs. Finally, cytokine analysis (Fig. 5L) revealed that pro-inflammatory cytokines, including IL-1β, TNF-α, IL-17a, and IL-23, were significantly reduced following PLEVPs treatment. Meanwhile, the Th17 differentiation involved cytokines of IL-12/IL-23 p40 and IL-12 p70 that were also notably decreased.

In parallel, we sought to determine whether the optimal PLEVPs hydrogel could also alleviate psoriasis symptoms after the full induction of the disease (Fig. S17A). The treatment visibly reduced erythema, scaling, and plaque severity (Fig. S17B) with noticeably reduced spleen size and calculated spleen index (Fig. S17C and G). Mice treated with PLEVPs hydrogel also showed stabilized body weight, reduced skin thickness, and decreased PASI scores (Fig. S17D to F), further confirming their therapeutic benefits. Histological analyses (Fig. S17H to J) showed reduced epidermal hyperplasia and immune cell infiltration, consistent with earlier results. Flow cytometry and cytokine quantification (Fig. S17K to M) revealed restored immune balance and decreased pro-inflammatory cytokines, reinforcing their role in overcoming psoriasis symptoms.

Collectively, these findings suggest that PLEVPs effectively alleviated psoriasis symptoms from multiple aspects, whereas the underlying mechanisms were expected to be uncovered.

### Topical administration of PLEVPs hydrogel modulates IL-17 pathway and gene expression in psoriatic skin inflammation

**Fig. 6.**
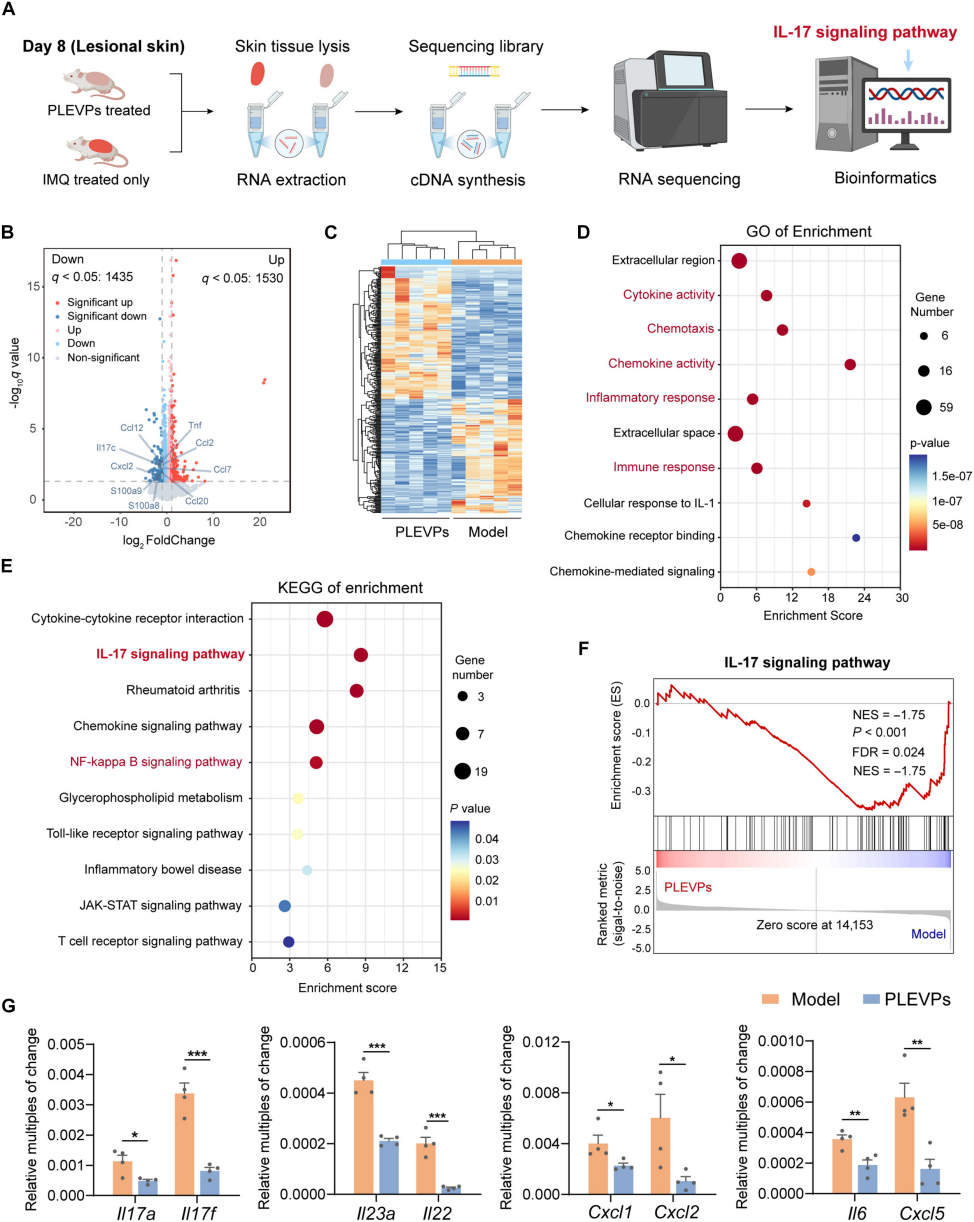
Transcriptomic analysis reveals significant down-regulation of IL-17 signaling pathway after topical treatment of PLEVPs hydrogel in psoriatic skin inflammation model. (A) Schematic workflow for RNA sequencing from lesional skin, including RNA extraction, cDNA synthesis, and bioinformatics analysis. (B) Volcano plot of differentially expressed genes between PLEVPs-treated and model groups (cutoff: |log2FC| > 1, *q* < 0.05). (C) Heatmap of significantly altered gene expression profiles between PLEVPs-treated and control groups (*n* = 5). (D) Gene Ontology (GO) enrichment analysis of biological processes related to cytokine activity, chemotaxis, and inflammatory response. (E) KEGG pathway enrichment analysis of key pathways modulated by PLEVPs treatment, including IL-17, NF-κB, and JAK-STAT signaling pathways. (F) Gene Set Enrichment Analysis (GSEA) plot of significantly enriched IL-17 signaling pathway comparing PLEVPs-treated and model groups. (G) qPCR validation of genes related to IL-17 signaling (*Il17a, Il17f, Il23a, Il22, Cxcl1, Cxcl2, Il6,* and *Cxcl5*) after PLEVPs treatment (*n* = 4). Data are presented as mean ± SD. Statistical analysis was performed using one-way ANOVA, with *P < 0.05, **P < 0.01, ***P < 0.001 indicating statistical significance.

To elucidate the regulatory mechanisms of PLEVPs in ameliorating IMQ-induced psoriasis symptoms, RNA sequencing analyses were further conducted. As shown in Fig. 6A, lesional skin from PLEVPs hydrogel-treated and IMQ-only groups was collected on day 8 for RNA extraction, followed by cDNA synthesis, sequencing, and bioinformatics analysis. First, principal component analysis (PCA, Fig. S18) demonstrated a clear separation between the PLEVPs-treated group and the IMQ-induced psoriasis model, suggesting distinct transcriptional profiles between these 2 groups. Notably, differential expression analysis (Fig. 6B) identified 1,530 up-regulated and 1,435 down-regulated genes in the PLEVP group compared to the model, with several psoriasis-related inflammatory markers (Il17a, Ccl2, Cxcl2, and Tnf) being significantly down-regulated. Further hierarchical clustering (Fig. 6C) and Gene Ontology (GO) enrichment analysis (Fig. 6D and Fig. S19) reinforced the role of PLEVPs in modulating immune and inflammatory responses. Specifically, enriched pathways included cytokine activity, immune response, and chemotaxis, all of which are critically involved in the progression of psoriasis. Specifically, Kyoto Encyclopedia of Genes and Genomes (KEGG) analysis (Fig. 6E) highlighted the IL-17 signaling pathway, known for its critical role in driving the inflammatory cascade in psoriasis, along with nuclear factor-kappa B (NF-κB) as another important pathway that is tightly involved in chronic inflammation. In this regard, Gene Set Enrichment Analysis (GSEA) confirmed both of their significant down-regulation (Fig. 6F and S20), together with heatmaps (Fig. S21) illustrating the overall suppression of genes. To validate these transcriptomic findings, we conducted quantitative polymerase chain reaction (qPCR) on select genes (Fig. 6G), focusing specifically on mediators of the IL-17 signaling pathway. The results confirmed significant reductions in primary effectors of *Il17a* and *Il17f*, along with their downstream targets of *Il23a*, *Cxcl1*, *Cxcl2*, and *Cxcl5*. Interestingly, *Il6* as not only a critical downstream target, but also an upstream trigger of positive-feedback loop through the activation of NF-κB and STAT [36], was also found to be significantly decreased. This suggests multistage modulation of PLEVPs on the IL-17 signaling pathway. Consistent with the qPCR results, the protein expression of IL-17 (Fig. S22) was also significantly down-regulated in the PLEVPs hydrogel-treated group. Taken together, these findings demonstrate that PLEVPs hydrogel exerts a comprehensive anti-inflammatory effect by specifically down-regulating the IL-17 signaling pathway.

### Endogenous Pab-miR396a-5p packaged by PLEVPs acts as a key regulator to mediate therapeutic effects

**Fig. 7.**
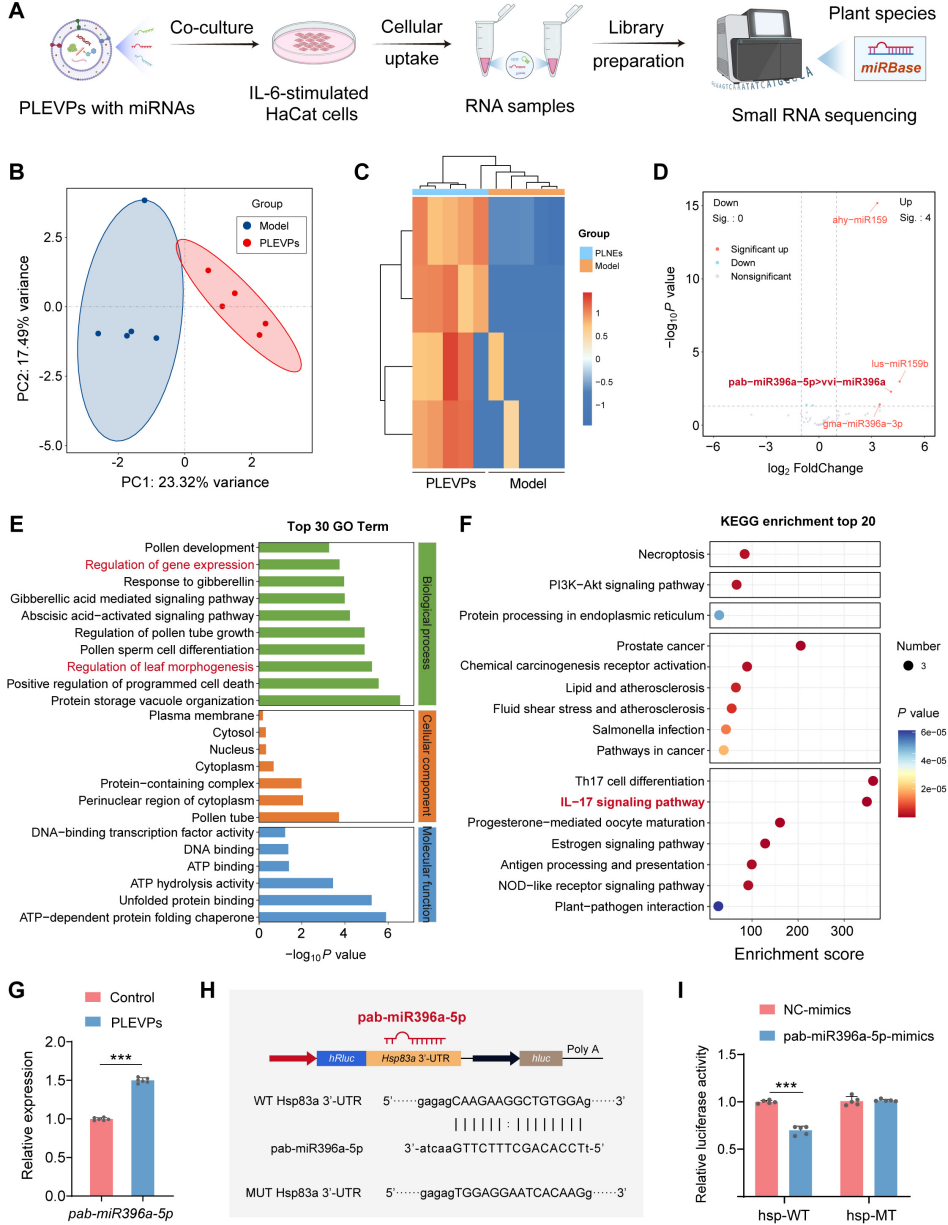
Small RNA sequencing reveals differential miRNA expression in IL-6-stimulated HaCaT cells treated with PLEVPs. (A) Schematic of the experimental workflow, including co-culture of PLEVPs with IL-6-stimulated HaCaT cells, followed by RNA extraction, library preparation, and small RNA sequencing using *Perilla* leaf-specific database. (B) PCA of small RNA expression profiles in PLEVPs-treated and control groups. (C) Heatmap of differentially expressed small RNAs between these 2 groups. (D) Volcano plot of significantly up-regulated small RNAs in PLEVPs -treated cells with emphasis on pab-miR396a-5p. (E) GO enrichment analysis of biological processes and molecular functions associated with gene expression regulation. (F) KEGG pathway enrichment analysis highlighting the IL-17 signaling pathway. (G) qPCR validation of pab-miR396a-5p expression in PLEVPs-treated and control groups. (H) Identification of HSP83a as a target mRNA of pab-miR396a-5p, followed by schematic of the dual-luciferase reporter assay constructs, showing wild-type (WT) and mutant (MT) sequences. (I) The interaction between pab-miR396a-5p and HSP83a validated by dual-luciferase assay. Data were presented as mean ± SD. Statistical analysis was performed using one-way ANOVA, with ***P < 0.001 indicating statistical significance.

Emerging evidence has pointed out that plant-derived miRNAs can regulate intercellular communication in human and animal cells [37], inspiring us to figure out whether miRNAs delivered by PLEVPs are key components to modulate IL-17 signaling pathway. To explore this, we co-cultured PLEVPs with IL-6-stimulated HaCaT cells and extracted RNA for small RNA sequencing (Fig. 7A). It is worth mentioning that the differential miRNA expression was identified using a *Perilla*-specific database, suggesting that the up-regulated genes were derived from PLEVPs due to successful internalization by the HaCaT cells. Among the detected miRNAs, pab-miR396a-5p was predicted as a key regulator through TargetFinder analysis, which evaluates sequence complementarity and binding stability, supporting its potential functional relevance in modulating IL-17-related inflammation. First, the PCA revealed clear separation between the model and PLEVPs-treated groups (Fig. 7B), indicating distinct small RNA expression profiles. Then, heatmap analysis of differentially expressed small RNAs (Fig. 7C) confirmed that several miRNAs were significantly up-regulated in the PLEVPs-treated group, including pab-miR396a-5p, as highlighted in the volcano plot (Fig. 7D). GO analysis (Fig. 7E) indicated significant enrichment in terms such as regulation of gene expression and programmed cell death, which are closely related to processes involved in psoriasis and inflammation. More importantly, KEGG pathway enrichment (Fig. 7F) revealed the IL-17 signaling pathway as a major target of these miRNAs, strongly linking the effects of PLEVPs to the regulation of this inflammatory pathway.

To further validate the role of pab-miR396a-5p as therapeutic cargo, we performed qPCR analysis on PLEVP-treated cells and confirmed a significant increase in their expression (Fig. 7G). Through bioinformatics analysis, we identified HSP83a as a plant-specific target of pab-miR396a-5p (Fig. 7H). Interestingly, the homologous target gene in animals is heat shock protein 90 (HSP90), a well-known molecular chaperone involved in various signaling pathways, such as IL-17 signaling. This finding aligns with previous studies reporting the involvement of HSP90 in skin inflammation [38]. To this point, we applied dual-luciferase reporter assay and confirmed that pab-miR396a-5p specifically binds to the HSP83a 3’-untranslated region (Fig. 7I), suggesting a potential regulatory role in animal cells as well.

In conclusion, our results demonstrate that pab-miR396a-5p delivered by PLEVPs could directly target HSP83a (HSP90) involved in the IL-17 signaling pathway, providing a potential mechanism for therapeutic effects of PLEVPs on psoriasis. Further studies are expected to investigate its regulatory impact on HSP90 and the downstream IL-17 pathway in target cells.

### Pab-miR396a-5p targets mammalian HSP90 to regulate IL-17 signaling and suppress psoriatic skin inflammation

**Fig. 8.**
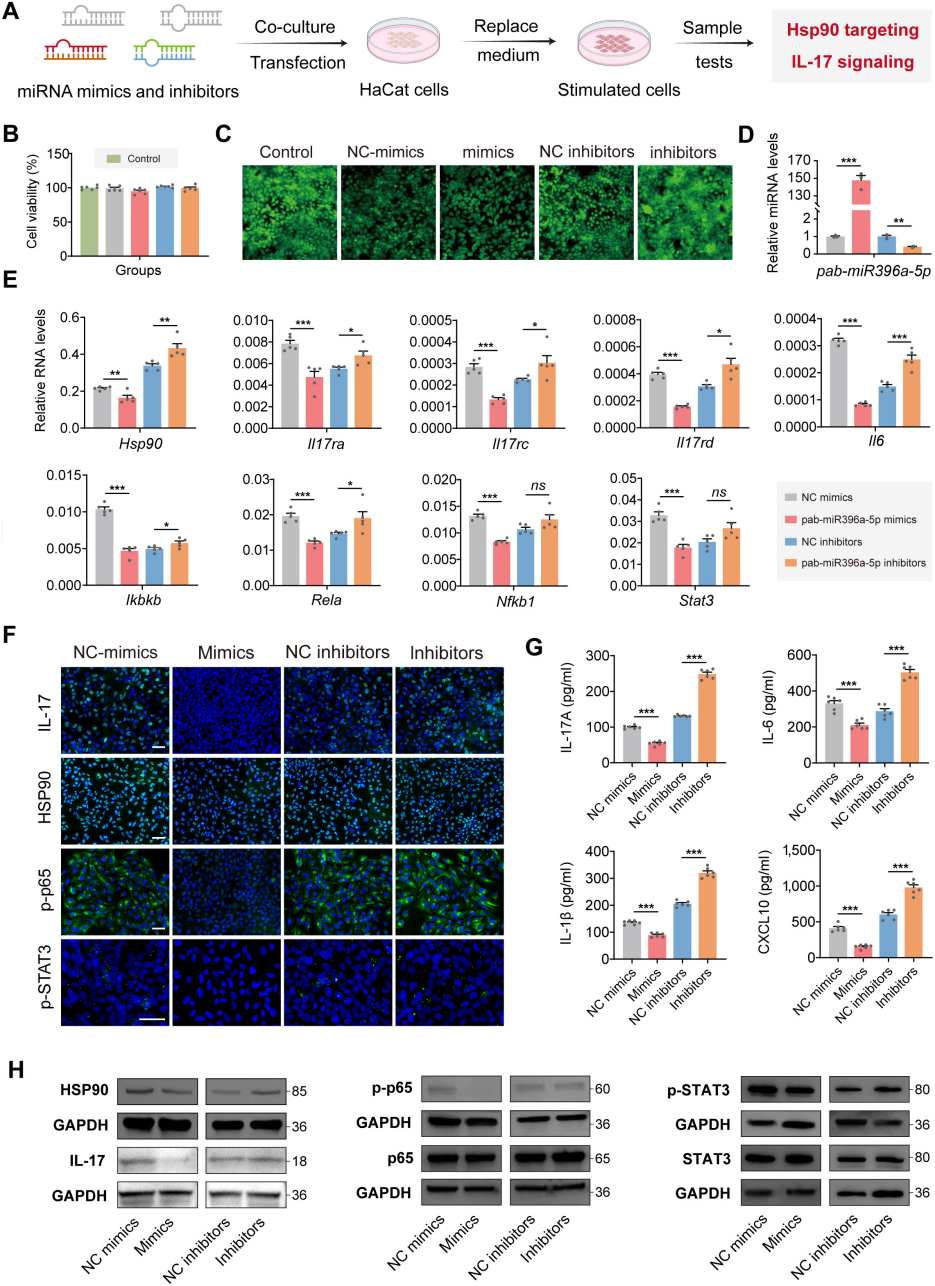
Overexpression and inhibition of pab-miR396a-5p regulate HSP90 and IL-17 signaling pathway activity in HaCaT cells. (A) Schematic diagram of the transfection and experimental workflow: pab-miR396a-5p mimics and inhibitors were transfected into IL-6-stimulated HaCaT cells, followed by the extraction of RNA and protein for downstream analyses. (B) Cell viability assay (CCK-8) after transfection with NC mimics, pab-miR396a-5p mimics, NC inhibitors, or pab-miR396a-5p inhibitors compared to the control group. (C) Live/dead cell staining images of HaCaT cells after transfection of miRNAs, where green fluorescence represents live cells. (D) qPCR analysis of pab-miR396a-5p expression levels in HaCaT cells transfected with miRNAs. (E) qPCR results for key genes in the IL-17 signaling pathway (*Hsp90, Il17ra, Il17rc, Il17rd,* and *Il6*) and genes involved in the NF-κB and JAK-STAT pathways (*Ikbkb, Rela, Nfkb1,* and *Stat3*) across different transfection conditions. (F) Immunofluorescence staining of IL-17, HSP90, p-p65, and p-STAT3 in HaCaT cells after transfection with different miRNA samples. Scale bar, 100 μm. (G) ELISA results of IL-17a, IL-6, IL-1β, and CXCL10 concentrations in the cell culture supernatants of transfected HaCaT cells. (H) Western blot analysis of HSP90, IL-17, p-p65, and p-STAT3 protein levels in HaCaT cells after different miRNA transfections. Data are presented as mean ± SD, *n* = 5. Statistical analysis was performed using one-way ANOVA, with *P < 0.05, **P < 0.01, ***P < 0.001 indicating statistical significance.

In our previous study, we demonstrated that PLEVPs could deliver pab-miR396a-5p to target HSP90 mRNA and potentially regulate the IL-17 signaling pathway in psoriasis. To further validate the specific role of pab-miR396a-5p in this regulatory mechanism, we synthesized its mimics and inhibitors along with their negative controls and transfected them into HaCaT cells to proceed with a series of functional validations (Fig. 8A). Cell viability was first assessed using the CCK-8 assay and live/dead cell staining, which confirmed that neither the mimics nor inhibitors caused significant cytotoxicity (Fig. 8B and C and Fig. S23), ensuring the feasibility of subsequent experiments.

After successful transfection, qPCR analysis revealed that *pab-miR396a-5p* expression in the mimics group increased by 147.7-fold compared to the negative control, while the inhibitors group exhibited a 60% reduction in miRNA levels (Fig. 8D). We next evaluated the expression of HSP90. As expected, this direct target of pab-miR396a-5p was significantly down-regulated in the mimics group. Following this, we evaluated critical genes involved in the IL-17 signaling pathway, including *Il17ra, Il17rc, Il17rd,* and *Il6*. These genes were markedly reduced in the mimics group, while their expression remained either slightly elevated or unchanged in the inhibitors group (Fig. 8E). This confirmed the suppressive effect of pab-miR396a-5p on IL-17 signaling. In addition, it has been evidenced that HSP90 acts as a molecular chaperone, stabilizing critical proteins involved in both the NF-κB and JAK-STAT pathways, which are upstream regulators of IL-17 signaling. Interestingly, the transcriptomic analysis also revealed significant enrichment of these pathways, aligning with our hypothesis of their involvement in IL-17 regulation. Therefore, relevant genes including *Ikbkb, Rela, Nfkb1, Ikbke, Jak2,* and *Stat3* were detected and observed to be significantly reduced in the mimics group (Fig. 8E and Fig. S24), showing the influence of pab-miR396a-5p down-regulating both the NF-κB and JAK-STAT pathways via HSP90 targeting.

Subsequent IF staining demonstrated these findings. The expressions of IL-17, HSP90, p-p65, and p-STAT3 were reduced in the mimics group, whereas the inhibitors group showed slightly elevated or similar expressions compared to the negative control (Fig. 8F). Consistent with these observations, ELISAs from the cell supernatants showed that inflammatory cytokines such as IL-17A, IL-6, IL-1β, and CXCL10 were significantly reduced in the mimics group but elevated in the inhibitors group (Fig. 8G). Finally, Western blot analysis (Fig. 8H) confirmed that critical protein levels of HSP90, IL-17, p-p65, and p-STAT3 were reduced in the mimics group, validating that pab-miR396a-5p inhibits HSP90 and its downstream inflammatory signaling pathways.

To further solidify our study, we conducted additional experiments combining PLEVPs with miRNA inhibitors to assess their role in modulating the IL-17 pathway. As shown in Fig. S25, PLEVPs treatment significantly reduced the expression of HSP90 and IL-17, while this effect was partially reversed upon pab-miR-396a-5p inhibition, as confirmed by Western blot analysis (Fig. S25A). The qPCR results (Fig. S25B) further demonstrated that PLEVPs down-regulated *Il17ra*, *Il17rd*, and *Il17rc*, whereas miRNA inhibition weakened this suppression. ELISA analysis (Fig. S25C) also showed that PLEVPs reduced key inflammatory cytokines (IL-1β, IL-17, and CCL2), but this effect was diminished when miRNA was blocked. Notably, NC inhibitors were included as scrambled controls in both PLEVPs and inhibitor groups to ensure specificity. These findings reinforce the critical role of pab-miR-396a-5p in mediating the therapeutic effects of PLEVPs, further elucidating their mechanism in modulating inflammatory signaling in psoriasis.

### Pab-miR396a-5p mimics encapsulated by lipid nanoparticles effectively prevent IMQ-induced psoriasis

**Fig. 9.**
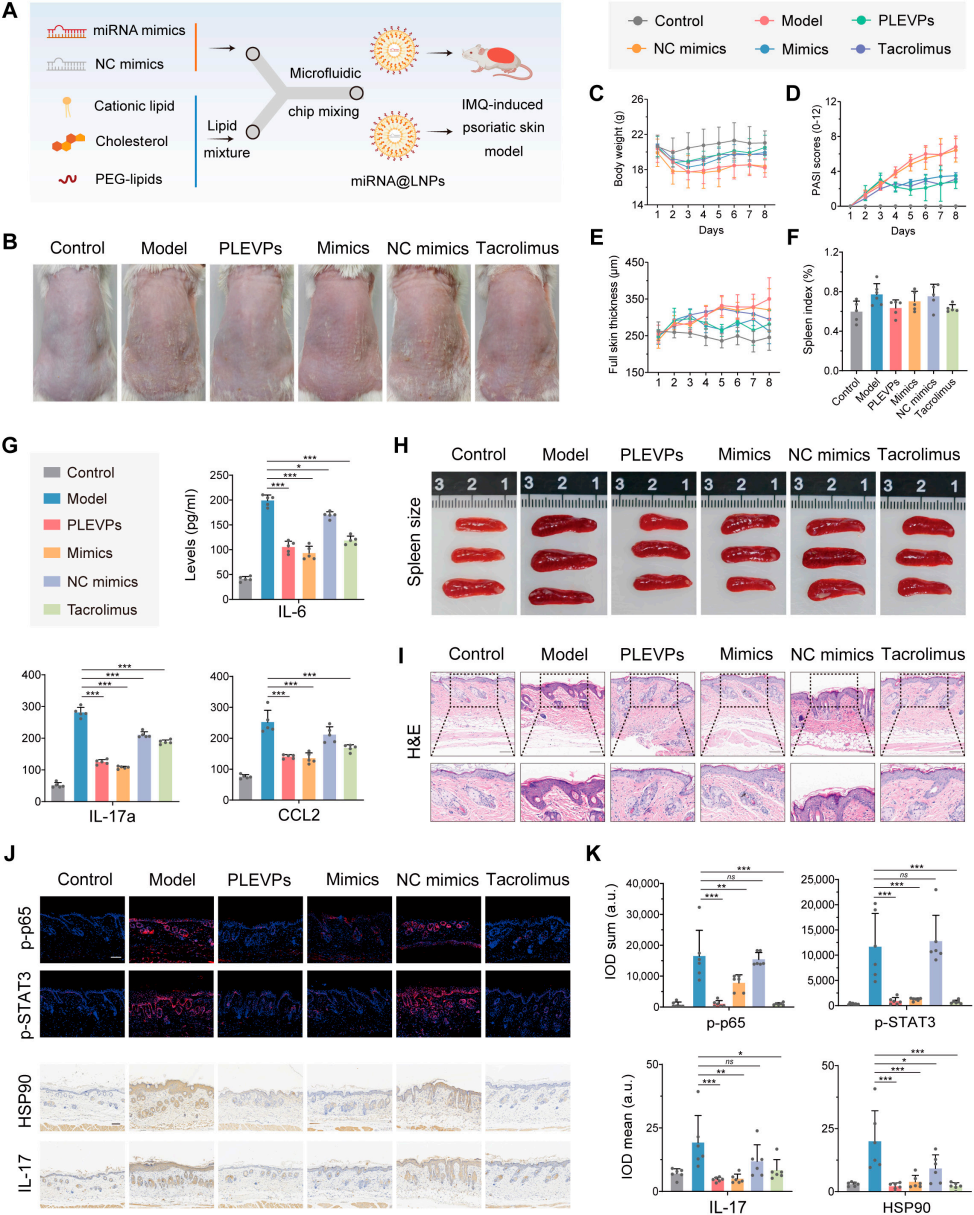
LNPs encapsulated pab-miR396a-5p mimics mitigate IMQ-induced psoriasis on mice. (A) Schematic of the process to encapsulate miRNA mimics and negative control (NC) mimics into lipid nanoparticles (LNPs) using microfluidic mixing, followed by administration to the IMQ-induced psoriasis-like mouse model. (B) Representative images of mouse dorsal skin lesions on day 8. (C) Body weight, (D) PASI scores, and (E) full skin thickness profile comparisons across all treatment groups over 8 days. The labels for the different treatment groups are shown in the gray box at the top of the figures. (F) Spleen index among different treatment groups. (G) ELISA analysis of serum inflammatory cytokines (IL-6, IL-17a, and CCL2) from different treatment groups. (H) Comparison of spleen size among different groups. (I) H&E staining of skin tissues from each group, highlighting histological differences and epidermal thickness. Scale bar, 100 μm. (J) IF staining of p-p65 and p-STAT3, and IHC analysis of IL-17 and HSP90 in skin tissue from each group. Scale bar, 100 μm. (K) Quantification of IF and IHC results, showing integrated optical density (IOD) measurements for p-p65, p-STAT3, IL-17, and HSP90. Data are presented as mean ± SD, *n* = 5. Statistical analysis was performed using one-way ANOVA, with *P < 0.05, **P < 0.01, ***P < 0.001 indicating statistical significance.

To further investigate the therapeutic efficacy of pab-miR396a-5p, we prepared lipid nanoparticles (LNPs) encapsulating miRNA mimics and their negative controls (NC mimics) using a microfluidic system (Fig. 9A). DLS measurements confirmed particle sizes of approximately 82.65 nm for pab-miR396a-5p mimics@LNPs and 79.74 nm for NC mimics@LNPs, with polydispersity index (PDI) values of 0.172 and 0.102 respectively, indicating uniform nanoparticle distribution (Fig. S26 and Table S1). TEM images revealed well-defined spherical morphologies for both LNPs (Fig. S27), and the encapsulation efficiencies of pab-miR396a-5p mimics and NC mimics were similarly high, at 98.37% and 98.62%, respectively (Table S1).

Regarding the in vivo application, we tested these LNPs in an IMQ-induced psoriasis mouse model, together with PLEVPs and Tacrolimus for comparison. Visual observation of dorsal skin (Fig. 9B and Fig. S28) indicated that PLEVPs showed the best skin conditions closer to the Control group. The mimics group also exhibited visible improvements compared to the NC mimics, which displayed similar scales and erythema to the model group. The results were consistent when analyzing spleen size, where both miRNA mimics and PLEVPs treatments significantly reduced spleen enlargement (Fig. 9H). Next, we produced body weight, PASI score, skin thickness profiles and monitored their spleen index (Fig. 9C to F). It can be observed that the miRNA mimics group regained body weight and exhibited significant reductions in PASI score and skin thickness, similar to PLEVPs and Tacrolimus. Notably, PLEVPs displayed the most pronounced improvement in PASI score, even outperforming Tacrolimus. Although spleen index differences were not statistically significant, miRNA mimics, PLEVPs, and Tacrolimus all trended toward reduced spleen index. Hematoxylin and eosin (H&E) staining (Fig. 9I) further supported these findings. NC mimics and the model group exhibited pronounced epidermal thickening and scaling. MiRNA mimics, while not as effective as PLEVPs, demonstrated reduced epidermal thickness and inflammation, reinforcing its potential role in modulating keratinocyte behavior.

**Fig. 10.**
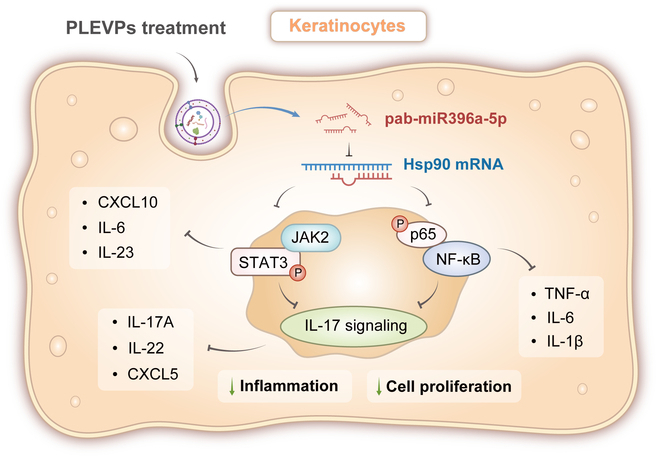
PLEVPs deliver pab-miR396a-5p to mediate down-regulation of the IL-17 signaling pathway. Upon PLEVPs treatment, pab-miR396a-5p is delivered into keratinocytes, where it targets and down-regulates HSP90 mRNA. This down-regulation inhibits both the JAK2/STAT3 and NF-κB (p65) signaling pathways. Inhibition of the JAK2/STAT3 pathway can reduce the production of CXCL10, IL-6, and IL-23, while suppression of the NF-κB pathway decreases TNF-α, IL-6, and IL-1β levels. Together, these effects lead to the down-regulation of IL-17 signaling, reducing the expression of IL-17a, IL-22, and CXCL5, ultimately resulting in decreased inflammation and keratinocyte proliferation.

ELISA results (Fig. 9G) showed that IL-6, IL-17a, and CCL2 levels were significantly reduced in both miRNA mimics and PLEVPs groups, with reductions comparable to Tacrolimus. Interestingly, miRNA mimics displayed a trend toward even lower levels of these cytokines compared to PLEVPs, although differences were not statistically significant. This might be due to the targeted overexpression of pab-miR396a-5p. Surprisingly, we observed the mild decrease in inflammatory cytokines in the NC mimics group, which is possibly attributed to the general anti-inflammatory properties of LNPs, which have been previously reported. In IF and IHC analyses (Fig. 9J and K), we observed significant down-regulation of p-p65 and p-STAT3 in the miRNA mimics and PLEVPs groups, consistent with our previous in vitro findings. This confirmed the suppression of IL-17 pathway activation through the inhibition of these upstream pathways. IHC results showed a pronounced reduction in IL-17 and HSP90 expression in the miRNA mimics group, indicating successful modulation of the target gene and suppression of psoriatic inflammation. In summary, the in vivo experiment validated the therapeutic potential of pab-miR396a-5p mimics in suppressing psoriasis by targeting HSP90 and inhibiting NF-κB, JAK-STAT, and IL-17 signaling (Fig. 10).

### PLEVPs demonstrate favorable safety

**Fig. 11.**
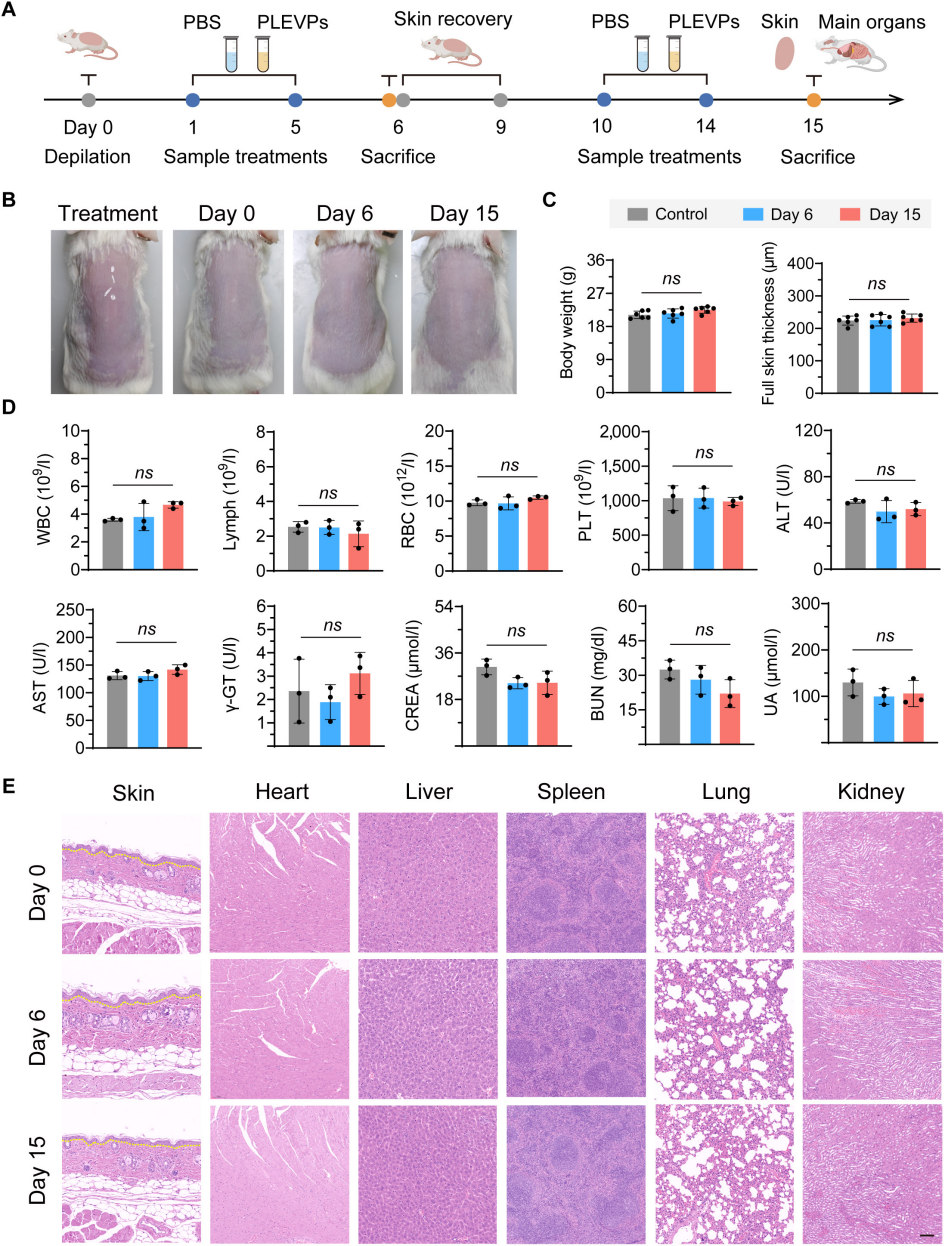
PLEVPs hydrogel show no significant toxicity in mice after repeated administration with recovery periods. (A) Experimental timeline of PLEVPs hydrogel administration and sample collection. Mice were depilated on day 0, followed by treatment with PLEVPs or PBS. After the initial treatment and skin recovery period, a second round of treatments was administered with sacrifices for safety assessments. (B) Representative images of dorsal skin during treatment and recovery periods at different time points (days 0, 6, and 15). (C) Measurements of body weight, full skin thickness at days 6 and 15 (*n* = 6). (D) Blood routine and biochemical examinations, including blood parameters (WBC, lymphocytes, RBC, and PLT) and serum biochemical markers for liver (ALT, AST, and γ-GT) and kidney (CREA, BUN, and UA) function (*n* = 3). (E) H&E examination of skin and major organs (heart, liver, spleen, lung, and kidney) at day 0, day 6, and day 15 to assess histopathological changes after PLEVPs treatment. Scale bar, 100 μm. Data were presented as mean ± SD.

To evaluate the safety of PLEVPs hydrogel, mice underwent 2 treatment cycles with recovery periods in between (Fig. 11A). Skin images on days 0, 6, and 15 showed no signs of irritation, with skin appearance comparable to controls (Fig. 11B). Body weight and skin thickness remained stable, with no significant differences between PLEVPs hydrogel and blank hydrogel (Fig. 11C). Additionally, trans-epidermal water loss (TEWL) measurements shown in Fig. S29A revealed no significant changes across treatment days, indicating that PLEVPs hydrogel did not compromise skin barrier integrity. Key inflammatory cytokines (IL-17A, IL-6, TNF-α, and IL-1β) of skin tissues were further evaluated with no significant differences between the PLEVPs hydrogel-treated groups and controls (Fig. S29B to E), suggesting minimal immune activation. Concerning the hematological and biochemical parameters including liver and kidney markers, no abnormalities were found comparing PLEVPs hydrogel-treated mice with control (Fig. 11D). The histopathology of skin and major organs (heart, liver, spleen, lung, and kidney) displayed no structural changes or inflammation (Fig. 11E). Furthermore, histological analysis of major organs from Control (blank hydrogel), PLEVPs, miRNA mimics, NC mimics, and Tacrolimus groups showed no signs of toxicity (Fig. S30), confirming the safety and biocompatibility of PLEVPs hydrogel and related formulations. These results collectively demonstrate the safety of PLEVPs, supporting their potential for therapeutic applications.

## Discussion

Psoriasis is a chronic inflammatory skin disorder driven by immune dysregulation, particularly through the IL-17 signaling pathway [39,40]. Conventional treatments including corticosteroids and biologics are often limited by side effects and efficacy concerns, highlighting the need for safer and more effective alternative [34]. Recently, plant-derived EVPs have gained attention due to their biocompatibility and potential to target inflammatory pathways [41]. In this work, we identified PLEVPs from multiple plant-derived EVPs as a potent candidate due to their strong anti-inflammatory and antioxidant effects in vitro. To explore the therapeutic potential, we incorporated PLEVPs into a simple hydrogel system and demonstrated that PLEVPs significantly reduced oxidative stress, modulated Treg cell levels, and enhanced keratinocyte apoptosis. Mechanistically, transcriptomic and small RNA analyses both revealed that PLEVPs down-regulate the IL-17 signaling. Further investigation pointed out the specific pab-miR396a-5p, as a key regulatory component within PLEVPs, whose overexpression and encapsulation in LNPs provide additional evidence of its therapeutic potential in psoriasis treatment.

Unlike mammalian-derived EVPs, often presenting immunogenic and zoonotic risks, plant-derived EVPs offer a more biocompatible alternative. We have previously shown, for instance, that ginseng-derived EVPs effectively modulate immune responses by influencing macrophage polarization [42]. Moreover, the scalability and sustainability of plant-derived EVPs perform more superior. They can be produced on a large scale at lower cost and bypass the ethical and resource-intensive challenges associated with animal sources. Additionally, compared to traditional TCM formulations, plant-derived EVPs demonstrate unique advantages, including enhanced stability, consistent delivery of bioactive components, and superior transdermal absorption. Likewise, they face challenges in storage stability since they are prone to aggregation and sedimentation, especially under long-term storage or varied environmental conditions. In this regard, we incorporated PLEVPs into a hydrogel matrix, which provided a stabilizing environment with a protective network to minimize interactions and prevent aggregation during prolonged storage. Meanwhile, psoriatic skin, characterized by a thickened epidermis and disrupted barrier function, poses critical challenges for drug penetration. The hydrogel enhances the retention of PLEVPs on the skin, allowing for sustained exposure and better therapeutic efficacy. In summary, this hydrogel system in our work serves as a useful tool, enabling practical and efficient studies of PLEVPs.

Next, to uncover the delivery cargo, recent studies have highlighted miRNAs within plant-derived EVPs, showing their anti-inflammatory and immune-modulating effects [29,30,43,44]. However, many of these studies either rely on mammalian small RNA libraries for miRNA identification or compare miRNA profiles between EVPs and plant juices. These approaches often face challenges in distinguishing plant-derived miRNAs from endogenous mammalian cellular responses [30,45]. In contrast, our study employed a *Perilla*-specific miRNA library, ensuring accurate identification of plant-derived miRNAs with minimal interference. This methodological advantage allowed us to identify pab-miR396a-5p as a unique regulatory miRNA packaged within PLEVPs. With the prefix of miR396a variants denoting specific plant source, it has been reported that Sp-miR396a-5p from tomato modulates stress tolerance [46], while aly-miR396a-5p from ginger has demonstrated antiviral and anti-inflammatory effects by inhibiting the NF-κB pathway [47]. Although these miRNAs may share similar or identical sequences, they can still show distinct biological activities and target preferences based on their plant origin and environmental context. This highlights the novelty of our work in demonstrating the specific regulatory role of *Perilla*-derived pab-miR396a-5p in modulating psoriasis-related pathways.

Concerning the mechanistic insight, the down-regulation of IL-17 signaling is critical in psoriasis pathogenesis. Notably, the small RNA sequencing also revealed enrichment in the IL-17 pathway, which is contributed by the pab-miR396a-5p within PLEVPs. Its target protein of HSP83a, as mammalian homolog of HSP90 [48,49], plays a pivotal role in psoriasis through its involvement in IL-17 signaling [50,51]. Studies have shown that the inhibition of HSP90 can attenuate IL-17-driven pathology, making it a promising target for managing psoriasis [52]. Furthermore, HSP90 serves a critical stabilizing function within NF-κB and JAK-STAT pathways [53,54], both upstream of IL-17 production. Lee et al. [55] found that the siRNA-mediated HSP90 knockdown considerably inhibited the NF-κB activation. Similarly, Kuusanmäki et al. [56] confirmed that the inhibition of HSP90 can disrupt STAT3 phosphorylation, directly impacting the JAK-STAT signaling pathway. Notably, while pab-miR396a-5p is enriched in the IL-17 pathway, our computational predictions did not identify direct binding sites for pab-miR396a-5p in the mRNAs of IL-17, IL-6, or CXCL10 (Table S2), indicating that its regulatory influence is exerted through upstream targets rather than direct cytokine suppression. This is consistent with the established principles of miRNA function, as miRNAs predominantly regulate intracellular signaling molecules and transcriptional regulators rather than directly targeting secreted cytokines [57,58]. In this context, the selective targeting of HSP90 by pab-miR396a-5p positions it as a critical modulator of IL-17 signaling, affecting cytokine expression indirectly by disrupting upstream regulatory networks. Collectively, these findings support the mechanistic pathway where pab-miR396a-5p-mediated HSP90 suppression leads to decreased stability and activation of NF-κB and JAK2/STAT3, ultimately down-regulating IL-17-driven inflammation.

In validating our miRNA findings, we selected LNPs to encapsulate miRNA mimics due to their ability to improve stability and cellular uptake, stimulating the encapsulation of EVPs. Interestingly, our ELISA results showed an unexpected anti-inflammatory effect in the NC mimics group, which may have resulted from the structure and components of LNPs that have demonstrated mild anti-inflammatory effects in prior studies. We also interestingly noticed that miRNA mimics yielded stronger target suppression compared to PLEVPs in ELISA analysis, which may be attributed to the direct gene interaction of the overexpressed miRNA. However, PLEVPs displayed superior overall therapeutic effects, likely due to their composite bioactivity, as they contain multiple functional components, as illustrated in Fig. S31. It outlines the potential drug component–target–disease–pathway network according to metabolomic analysis. The identified compounds possess extra potential to modulate IL-17 and associated pathways, contributing synergistic effects to PLEVPs. Meanwhile, the observed Treg cell modulation may also be attributed to these bioactive metabolites, together with the association to the anti-inflammatory environment established by the suppression of IL-17 signaling via pab-miR396a-5p.

While the study provides promising insights into the therapeutic potential of PLEVPs, a few limitations should be noted. First, the use of a single keratinocyte cell line, while suitable for initial studies, may not capture the full immune complexity of psoriasis, particularly the involvement of immune cells. To address this, future studies could incorporate immune cell-specific models to deepen our understanding of PLEVPs’ effects across different cellular environments. Secondly, the underlying mechanisms of skin penetration and cellular uptake of PLEVPs were not fully explored here. As transdermal delivery is essential for topical treatments, special focus can be put on in our future work as well. Additionally, the potential combination of PLEVPs with traditional TCM formulations offers a meaningful direction for future research and aligns with the modernization and integration of TCM and advanced drug delivery systems. While the hydrogel established in this study is simple and convenient, we are also making efforts to explore advanced formulation strategies, such as genetic engineering and cutting-edge drug delivery techniques, e.g., microneedles, to optimize PLEVPs for more effective and versatile therapeutic applications [59,60].

## Conclusion

In conclusion, our findings highlight the therapeutic potential of PLEVPs in alleviating psoriasis. We put the focus on understanding the active role of PLEVPs and their encapsulated miRNAs, specifically pab-miR396a-5p, in regulating key inflammatory pathways of IL-17 signaling and downstream inflammatory pathways. This research not only validates the efficacy of PLEVPs in alleviating psoriatic symptoms but also sheds light on their potential as natural nanocarriers for miRNA-based therapies, expanding the scope of plant-derived vesicles for targeted topical drug delivery and cross-kingdom therapeutic applications. The findings also lay the groundwork for future optimization and clinical exploration of plant-derived EVPs in dermatological therapies.

## Materials and Methods

### Cell lines

The human epidermal keratinocyte HaCaT cells and 293T cells were purchased from the Cell Bank of Shanghai Institutes for Biological Sciences, Chinese Academy of Sciences (China). The HaCaT cells were maintained at 37 °C with 5% CO_2_ and cultured in Dulbecco’s modified Eagles medium supplemented with 10% fetal bovine serum and 1% penicillin-streptomycin.

### Animals and ethical statements

Male Balb/c mice (4 to 6 weeks), weighing approximately 18 to 22 g, were supplied by GemPhar-matech (Nanjing, China). All animals were housed at room temperature (25 °C) with 40% to 70% humidity under a 12:12 h light–dark cycle. All animal procedures adhered to the Jiangsu Institute of Traditional Chinese Medicine’s guidelines [Approval No. SYXK (SU) 2021-0025].

### Preparation and characterization of PLEVPs

Ten kinds of plants (*Perilla frutescens* Britt, *Panax ginseng* C. A. Mey, *Zingiber officinale* Roscoe, *Coptis chinensis* Franch., *Panax notoginseng* F. H. Chen, *Angelica sinensis* Diels, *Agastache rugosa* Kuntze, *Mentha haplocalyx* Briq, *Astragalus membranaceus* Bunge, and *Curcuma longa* L.) were kept in a controlled environment for a period to help minimize the impact of external environmental factors. Then, they were washed and ground in a blender to obtain fresh juices. The juices were processed with sequential centrifugations (AVANTI J-26XP, Beckman, USA) at 800×*g* for 10 min, 2,500×*g* for 30 min, and 10,000×*g* for 1 h at 4 °C. Nano-exosomes in supernatants were then centrifuged at 100,000×*g* for 1 h with ultracentrifugation (Beckman XPN-100, Beckman, USA). The sediments were resuspended with ddH_2_O and further purified in a sucrose gradient (8%, 15%, 30%, 45%, and 60% sucrose) by ultracentrifugation at 100,000×*g* for 1 h. The band at 45% sucrose layer was collected and noted as the final plant-derived nano-exosomes. The nano-exosome suspensions were partially filtered with 0.22-μm microporous membrane and stored at −80 °C until use. The morphologies of nano-exosomes were observed by TEM (Talos L120C, Thermo Fisher Scientific, USA). The protein concentrations of nano-exosomes were determined using the BCA Protein Assay kit. The particle numbers and size distributions were measured with NTA using a NanoSight NS300 system (Malvern Instruments, UK). The surface charge was measured using laser diffraction spectrometry (Malvern Zetasizer 3000HS, Malvern, UK).

### Lipidomic analysis

PLEVPs lipids were extracted according to the methyl tert-butyl ether (MTBE) method. In brief, the PLEVPs samples were spiked with internal lipid standards, homogenized with water and methanol, and then subjected to lipid extraction by adding MTBE. After ultrasound treatment and centrifugation, the organic phase was collected, dried under nitrogen, and reconstituted in 90% isopropanol/acetonitrile. Lipid separation was performed using reverse-phase LC on a CSH C18 column (1.7 μm, 2.1 mm× 100 mm, Waters), and subsequent mass spectrometric analysis was conducted in both positive and negative modes on a Q-Exactive Plus instrument. Electrospray ionization (ESI) parameters were optimized, and data were processed using LipidSearch, with mass tolerance set to 5 ppm for both precursor and fragment ions.

### Metabolomic analysis and batch consistency

To compare metabolites between PLEVPs and *Perilla* leaf samples, metabolites were extracted and analyzed via mass spectrometry. Data were processed with R XCMS for feature detection, retention time correction, and alignment. Metabolites with an RSD > 30% in QC samples were excluded. Identification was performed by matching accurate mass and MS/MS data with databases including HMDB, LipidMaps, KEGG, and ChEBI, with a focus on identifying TCM metabolites using a specialized TCM database. Molecular formulas were predicted from *m*/*z* values, and MS/MS fragmentation patterns were matched to confirm metabolite identities.

To assess batch consistency, 3 independent PLEVPs batches were analyzed. SDS-PAGE was performed to visualize the protein composition of each batch, allowing for direct comparison of protein banding patterns and potential variations in vesicle-associated proteins. Additionally, metabolomic profiling was conducted by generating comparative pie charts based on identified metabolite categories across the 3 batches.

### Cell viability, proliferation assays, and cellular uptake

For the cell viability assay, HaCaT cells were seeded at a density of 6 × 10^3^ cells per well in a 96-well plate and incubated overnight to allow for cell attachment. Cells were then treated with various concentrations of PLEVPs (2, 5, 10, 25, and 50 μg/ml) for 48 h. After treatment, CCK-8 reagent was added to each well. The absorbance was measured at 450 nm using a microplate reader (Thermo Fisher Scientific Co., Ltd., China). Cell viability was calculated using the following equation: Cell viability (%) = OD_Treated_/OD_Control_ × 100%, where OD_Treated_ represents the optical density (OD) of treated cells, and OD_Control_ represents the OD of untreated cells.

For the cell proliferation assay, HaCaT cells were seeded at a lower density of 3 × 10^3^ cells per well in a 96-well plate to allow for continuous growth measurement over time. After IL-6 stimulation to induce a hyperproliferative state, cells were treated with varying concentrations of PLEVPs (2, 5, 10, 25, and 50 μg/ml) and incubated for 24, 48, and 72 h in a humidified incubator at 37 °C with 5% CO₂. Cell proliferation was measured at each time point using the CCK-8 assay, and the percentage of cell growth was normalized against untreated control cells.

For cellular uptake studies, HaCaT cells and IL-6-stimulated HaCaT cells were incubated with fluorescently labeled PLEVPs (DIR- and DIO-stained) for 24 h. After incubation, cells were fixed with 4% paraformaldehyde, followed by staining with an antifade mounting medium containing 4′,6-diamidino-2-phenylindole (DAPI). The cellular uptake of PLEVPs was visualized using a confocal laser scanning microscope (CLSM, Fluoview FV10i, Olympus Corporation, Japan).

### In vitro ROS detection

A ROS Assay Kit was utilized to detect the ROS level in HaCaT cells according to the manufacturer’s instruction. In brief, IL-6-stimulated cells were treated for 24 h. DCFH-DA was diluted with serum-free medium and added to the cell suspension, followed by incubation for 30 min at 37 °C in the dark. The fluorescence images were visualized and recorded using a CLSM. Following trypsin digestion, the cells were resuspended in DPBS, and the intracellular fluorescence intensity was assessed by flow cytometry (Guava easyCyte HT Flow Cytometry, Merck Millipore, Germany) and the fluorescence intensity was calculated and compared. On the other hand, the cells were incubated in confocal dishes following the same procedures. The ROS levels were monitored using CLSM to visualize the ROS scavenging abilities.

### Evaluation of inflammatory cytokines

After treating HaCaT cells with IL-6 for 24 h, the cells were co-cultured with various plant-derived nano-exosomes for an additional 24 h. After collecting the cell supernatant, the levels of inflammatory cytokines IL-6 and IL-1β were measured using ELISA kits (Hangzhou Lianke Biotechnology Co., Ltd., Hangzhou, China). Mouse skin tissues were collected to measure the expression levels of inflammatory cytokines (IL-1β, TNF-α, IL-17a, IL-23, IL-12 p70, and IL-12/IL-23 p40) with an ELISA Kit according to the manufacturer’s instructions. The cytokines of HaCaT cells with transfected miRNA mimics and inhibitors were also detected with ELISA Kit (IL-6 and IL-1β).

### Establishment of the IMQ-induced psoriasis mouse skin model

The day before establishing the psoriasis mouse skin model, a 2.5 × 2.5 cm area on the backs of the mice was depilated. Subsequently, 62.5 mg of IMQ was uniformly applied to the depilated area for 7 continuous days. The therapeutic efficacy of PLEVPs during psoriasis induction was first investigated. Briefly, the mice were randomly divided into 5 groups: Control, Model (phosphate-buffered saline, PBS), Low dose (0.2 mg/ml PLEVPs), Medium dose (0.5 mg/ml PLEVPs), and High dose (1 mg/ml PLEVPs), with 7 mice per group. From day 3 of IMQ administration, the treatment groups were applied with 200 μl of PLEVPs 4 h after the IMQ treatment, while the Control and Model groups were applied with an equal amount of PBS. The body weight, full skin thickness, and PASI scores were monitored daily. Then, the mice were sacrificed on day 8 for further analysis. The therapeutic efficacy of PLEVPs after the successful establishment of psoriasis mice skin models were also evaluated to reconfirm the therapeutic potential of PLEVPs. In brief, the mice were randomly divided into 3 groups: Control, Model (PBS), and PLEVPs (optimal dose concentration). After 7 days of IMQ application, 200 μl of PLEVPs was applied for an additional 5 days. The Control and Model groups were administered an equivalent amount of PBS. The mice were sacrificed on day 14 and the same parameters were recorded and analyzed.

### Preparation of PLEVPs incorporated hydrogel

Carbopol hydrogels were prepared by dispersing 1% (w/v) Carbopol 940 into deionized water with continuous magnetic stirring at 500 rpm for 2 h to allow complete swelling. Subsequently, PLEVPs were added to the swollen Carbopol solution at final concentrations of 0.2, 0.5, and 1 mg/ml. The mixture was stirred at 4 °C for 20 min to ensure uniform dispersion of the nanoparticles. The pH of the formulation was then adjusted to 7.0 using 5% NaOH under continuous stirring until a homogeneous hydrogel with suitable viscosity for topical application was obtained. The resulting hydrogel formulations were stored at 4 °C in dark conditions until further use.

### Storage stability of PLEVPs in hydrogel formulations

The stability of PLEVPs in hydrogel formulations was evaluated over 60 days by analyzing various parameters. Different concentrations of samples were collected on days 0, 7, 30, and 60, and stability was evaluated using NTA to measure particle size distribution, zeta potential to determine surface charge, and TEM to observe the morphological integrity of PLEVPs within the hydrogel matrix. Visual inspections were also performed to check for any phase separation or aggregation in the formulations. These parameters ensure comprehensive insights into the stability of PLEVPs when incorporated into the hydrogel system.

### In vitro drug release and skin retention studies

The in vitro release profile of PLEVPs from hydrogel and solution was evaluated using a dialysis bag method. Briefly, 1 ml of PLEVPs hydrogel or solution (containing 0.2, 0.5, or 1 mg/ml PLEVPs) was placed into a dialysis bag (molecular weight cutoff 8,000 to 12,000 Da) and immersed in 10 ml of PBS (pH 7.4) at 37 ± 0.5 °C under constant stirring (100 rpm). At predetermined time points (0, 6, 12, 24, 36, 48, 60, and 72 h), 1 ml of the release medium was collected and replaced with fresh PBS to maintain sink conditions. The released PLEVPs were quantified using a micro BCA protein assay kit (Beyotime, Shanghai, China) by measuring absorbance at 562 nm.

The in vivo retention studies were conducted using IMQ-induced psoriasis mouse models. After 7 days of topical IMQ application on the dorsal skin to induce psoriasis, mice were treated with 100 μl of FITC-labeled PLEVPs formulated as a solution or incorporated into a hydrogel. To evaluate retention and penetration efficiency, an in vivo imaging system (IVIS Lumina XR, PerkinElmer, USA) was used at designated time points (1 and 6 h) with an excitation wavelength of 488 nm and an emission wavelength of 520 nm. Before imaging, the dorsal skin was carefully cleaned to remove any remaining formulation on the surface, ensuring accurate fluorescence measurements. At 6 h posttreatment, mice were sacrificed, and their dorsal skin was immediately harvested and frozen in liquid nitrogen. The skin samples were embedded in optimal cutting temperature (OCT) compound, and 10-μm-thick cryosections were prepared using a cryostat. Sections were mounted onto glass slides, and nuclei were counterstained with DAPI for 10 min in the dark. Fluorescence distribution of FITC-labeled PLEVPs was imaged using a fluorescence microscope (Olympus FV10i, Olympus, Japan) with excitation/emission wavelengths of 495/520 nm for FITC and 360/460 nm for DAPI.

### Evaluation of transdermal penetration efficiency

In the depilated areas of healthy mice and psoriatic-like mice, FITC-labeled PLEVPs and free FITC solution were uniformly applied. A 2-photon microscope (STELLARIS 8 DIVE, Leica, Germany) was used to perform *z*-axis scanning of the skin, with the scanning depth set to 50 μm. The scanned images were extracted at different depths and the reconstructed 3D images were compared.

### Back lesion scoring and spleen index measurement

The severity of psoriatic-like inflammation of mice was assessed using the PASI scoring system. The principle is as follows: the severity of skin inflammation is evaluated daily using a 5-point scale (0 to 4) for erythema, scaling, and thickening. Each parameter is scored based on severity from 0 to 4: 0, none; 1, slight; 2, moderate; 3, severe; and 4, very severe. The sum of the 3 parameter scores represents the severity of psoriatic dermatitis (0 to 12 points). The spleens of the mice were also collected, measured, and weighed after sacrificing the mice. The spleen index was calculated for each group using the following equation: Spleen Index = Spleen weight (g)/Mouse body weight (g) × 100.

### Histological, IHC, and IF analyses

All back skin of mice were collected and fixed with 4% paraformaldehyde, Afterward, the skin was embedded into paraffin and sliced into sections with 4 μm thickness. For histological analysis, the skin sections were stained with H&E. The expression of Ki67, IL-17, and IL-22 was evaluated with IHC staining. The expressions of Caspase 3, CD45, and CD4 were assessed with IF staining. All stained tissues were photographed under a digital slide scanner (Pannoramic MIDI, 3DHISTECH, Hungary). Meanwhile, in the in vivo biocompatibility studies, major organs of mice were implemented with H&E staining following the same procedure of scanning and observation.

### Flow cytometry analysis

The spleen of each mouse was collected and grounded into a single-cell suspension for flow cytometry staining. Different fluorescent dye-conjugated antibodies (anti-CD45, anti-CD3, anti-CD8, anti-CD4, and anti-Foxp3) were added into the single-cell suspension. The Foxp3 buffer set was used for better Foxp3 staining. After the incubation at 4 °C for around 20 min, the cells were analyzed using a flow cytometry (FACS Aria II, BD, USA).

### Blood routine and blood biochemical examination

Blood samples of different groups were collected before the experiments. The serum was acquired by centrifugation for 15 min at 3,000 rpm and 4 °C. Then, the white blood cells (WBCs), lymph cells (Lymph), red blood cells (RBCs), and platelet (PLT) were measured for blood routine tests, and serum levels of alanine aminotransferase, aspartate aminotransferase (ALT), gamma-glutamyl transferase (γ-GT), creatinine (CREA), blood urea nitrogen (BUN), and uric acid (UA) were determined for blood biochemical examination.

### Trans-epidermal water loss

TEWL is commonly used as an indicator of skin barrier integrity. In this study, TEWL was measured using a closed-chamber VAPO SCAN AS-VT100RS (Asch Japan Co., Ltd., Tokyo, Japan) to evaluate potential skin irritation following PLEVPs hydrogel application. Measurements were conducted at room temperature (22 ± 1 °C) and 50% ± 5% relative humidity. For each mouse, TEWL was assessed on both the left and right dorsal skin, with 2 repeated measurements per site and was recorded on day 0 (pre-treatment), day 6, and day 15 in safety analysis to monitor potential changes in skin barrier function over time.

### RNA sequencing

The back dorsal skins from Control, Model, and PLEVPs-treated groups were collected after sacrificing the mice. The skin tissues were lysed with Trizol reagent to extract total RNA. The purity and quantity of RNA were detected using NanoDrop 2000 (Thermo Fisher Scientific, USA). The libraries were then constructed and sequenced on an Ilumina Novaseq 6000 platform. The differential expression analysis was performed with parameters of *q* value < 0.05 and foldchange > 2 or foldchange < 0.5 set as the threshold for significant expressed gene. Based on hypergeometric distribution, the GO enrichment analysis and KEGG database pathway enrichment analyses were conducted respectively. The GSEA was performed using GSEA software. OE Biotech Co., Ltd. (Shanghai, China) conducted the bioinformatic analyses.

### Western blotting analysis

Skin tissues and HaCaT cells were respectively collected and subjected with the RIPA lysis buffer (Beyotime Biotechnology Co. Ltd., Shanghai, China) added with protease and phosphatase inhibitors to fully extract protein. The supernatants were then collected after centrifugation and the protein concentration was measured with the BCA Protein Assay Kit. After heating the sample at 100 °C for 6 min, 5× loading buffer (Beyotime Biotechnology Co. Ltd., Shanghai, China) was added. Then, protein samples were separated using SDS-PAGE and subsequently transferred onto a polyvinylidene difluoride (PVDF) membrane through electrophoresis. Protein Free Rapid Blocking Buffer (1×) (Shanghai Epizyme Biomedical Technology Co., Ltd, China) was further used to block the membrane, followed by overnight incubation at 4 °C with primary antibodies including anti-IL-17, anti-GAPDH. The membrane was then washed 3 times with TBST and incubated with horseradish peroxidase-conjugated secondary antibodies for 1 h. Protein bands were visualized using the Omni-ECL Femto Light Chemiluminescence Kit (Shanghai Epizyme Biomedical Technology Co., Ltd, China). Then, the membrane was stripped using a stripping buffer for 30 min, re-blocked, and incubated with additional primary antibodies or the reference antibody (β-actin). The bands were then quantified with ImageJ software (National Institutes of Health, USA) for comparisons.

### Protein and RNA characterization of PLEVPs

The characterization refers to the comparison of PLEVPs and *Perilla* leaf samples. Protein extracts were prepared with the ultrasound method. After centrifugation, both PLEVPs and leaf samples were prepared by adding 5× SDS-PAGE loading buffer, followed by denaturation at 100 °C for 10 min. The proteins were then separated on a 12% SDS-PAGE gel and visualized to compare the protein profiles between the 2 sources. In addition, total RNA was isolated using a Trizol-based extraction method and analyzed on a 1% agarose gel to assess RNA integrity, with the 28S and 18S rRNA bands serving as indicators.

### Real-time RT-PCR arrays and RT-PCR

Skin tissue or HaCaT cells were collected for isolating total RNA with Trizol reagent (Invitrogen, Carlsbad, CA, USA). Following the manufacturer’s procedures, RNA was reverse-transcribed using the iScriptTM cDNA Synthesis kit (Bio-Rad, Hercules, CA, USA). Real-time PCR Mouse and Human Array Plates (WC-MRNA0217-M and WC-MRNA0217-H, Wcgene biotech, China) were used to analyze the gene expression profiles. Real-time PCR was carried out with a QuantStudio 3 PCR machine (Applied Biosystems, Waltham, MA). Relative gene expression levels were normalized with housekeeping gene GAPDH (glyceraldehyde-3-phosphate dehydrogenase). Concerning the miRNA extraction, kit was purchased from BAIDAI (51094, China).

### Small RNA sequencing

HaCaT cells were exposed to IL-6 and subsequently co-incubated with PLEVPs for 24 h. Following the incubation, both cells and PLEVPs-uptake cells were subjected to small RNA sequencing. In particular, total RNA of cells was isolated with Trizol reagent (Invitrogen, CA, USA). The RNA quantification and integrity were assessed as abovementioned. A small RNA library was prepared using NEBNext Small RNA Library Prep Set for Illumina kit according to the manufacturer’s instructions. After reverse-transcribing RNA to cDNA and performing PCR amplification, small RNA libraries were sequenced using the Illumina Novaseq 6000 platform. Mature miRNAs were identified through the miRBase database, and their expression profiles were generated. Differential expression of miRNAs was determined using a *q*-value threshold of <0.05 and fold change criteria of >2 or <0.5. GO enrichment and KEGG pathway enrichment analyses were performed separately for differentially expressed miRNAs. The sequencing and subsequent analysis were supported by OE Biotech Co., Ltd. (Shanghai, China).

### Dual-luciferase reporter assay

According to the small RNA sequencing results, miR-pab-396a-5p excels to potentially influence IL-17 signaling pathway by targeting upstream regulatory genes. Bioinformatics analysis predicted HSP83a as a potential target of pab-miR-396a-5p, based on a high binding score. Then, both wild-type (WT) and mutant (MT) versions of the sequence were cloned into the pmirGLO vector. To validate the interaction, 293T cells were co-transfected with the pmirGLO-HSP83a-WT or pmirGLO-HSP83a-MT plasmid along with either pab-miR-396a-5p mimics or a negative control (NC). This experimental design resulted in 4 groups: (a) NC mimics + hsp-WT, (b) pab-miR-396a-5p mimics + hsp-WT, (c) NC mimics + hsp-MT, and (d) pab-miR-396a-5p mimics + hsp-MT. After 48 h, luciferase activity was measured using a dual-luciferase reporter assay kit, and the ratio of Firefly to Renilla luciferase activity was calculated for comparisons.

### miRNA transfection and co-incubation investigation

HaCaT cells (2.0 × 10^5^ cells/well) were seeded into 24-well plates and cultured until they reached 60% to 70% confluence. Subsequently, Pab-miR396a-5p mimics (final concentration: 50 nM) and NC mimics (50 nM) were transfected into the prepared cells using CALNP RNAi reagent (D-Nano Therapeutics, DN001), followed by incubation for 24 h. Pab-miR396a-5p inhibitors (100 nM) and NC inhibitors (100 nM) were introduced into cells using Rfect V2 siRNA reagent (Baidai, Changzhou, China), followed by incubation for 24 h. Afterward, the medium was replaced with fresh medium containing 25 ng/ml IL-6 for stimulation, and after 24 h, the supernatant and cells were collected.

To further validate the role of pab-miR396a-5p in mediating the effects of PLEVPs, an additional co-incubation experiment was performed, where PLEVPs and miRNA inhibitors were co-administered to evaluate whether miRNA inhibition could attenuate the effects of PLEVPs. Similarly, all samples were stimulated with IL-6 and the NC inhibitor was used as a scrambled negative control, ensuring that any observed effects were specifically attributed to the inhibition of pab-miR396a-5p rather than nonspecific effects of inhibitor transfection. The experimental groups in this study included the following: (a) Model group + NC inhibitors, (b) Model group + PLEVPs + NC inhibitors, and (c) Model group + PLEVPs + miRNA inhibitors.

For qRT-PCR analysis, miRNA was extracted from the cells using a BIOG Cell miRNA Extraction Kit (51094, Baidai, Changzhou, China), and the expression level of miR396a-5p was analyzed by qRT-PCR using an All-in-One miRNA qRT-PCR Detection Kit 2.0 (QP115, GeneCopoeia, Inc.) and All-in-One miRNA qPCR Primer, with the primer sequences listed in Table S3. Total RNA was extracted from the cells using a YALEPIC Animal Cell & Tissue Total RNA Fast Isolation Kit (YR23017, YALI Biotech Co., Ltd), and after reverse transcription using a HiScript IV RT SuperMix for Qpcr Kit (R423-01, Nanjing Vazyme Biotech Co., Ltd), the expression of target genes was quantified using ChamQ Universal SYBR qPCR Master Mix (Q711, Nanjing Vazyme Biotech Co., Ltd) and PCR Array plate (Shanghai WcGene Biotechnology Co., Ltd).

For IF staining, HaCaT cells were seeded onto sterile coverslips and transfected with either mimics or inhibitors, followed by IL-6 stimulation as described above. Cells were washed with PBS, fixed with 4% paraformaldehyde for 15 min, and permeabilized with 0.1% Triton X-100 for 10 min. Nonspecific binding was blocked using 5% bovine serum albumin for 1 hour. Cells were then incubated overnight at 4 °C with primary antibodies against IL-17, HSP90, p-p65, and p-STAT3. After washing, cells were incubated with fluorescently labeled secondary antibodies for 1 h at room temperature in the dark. Finally, nuclei were counterstained with DAPI, and coverslips were mounted with antifade mounting medium before imaging under a fluorescence microscope.

### Statistical analysis

Statistical analyses were performed using GraphPad Prism 9.0 software. Data were presented as means ± SD, with biological replicates employed unless otherwise specified. Statistical significance was assessed using Student’s *t* test for 2 group comparisons and one-way analysis of variance (ANOVA) for multiple group comparisons. A *P* value of less than 0.05 was considered statistically significant; ns denotes no significance. Levels of significance are denoted as follows: *P < 0.05, **P < 0.01, ***P < 0.001.

## Data Availability

The data are freely available upon request.
