# Supplementary material for: Perilla frutescens Leaf-Derived Extracellular Vesicle-Like Particles Carry Pab-miR-396a-5p to Alleviate Psoriasis by Modulating IL-17 Signaling
Source: Research (Wash D C). 2025 Apr 17;8:0675. doi: 10.34133/research.0675 (PMC12003952; doi:10.34133/research.0675)
Supplement: Supplementary 1 — Figs. S1 to S31 Tables S1 to S3 [file research.0675.f1.docx]

**Supplementary Materials**

**Title**

***Perilla frutescens* Leaf-Derived Extracellular Vesicle-like Particles Carry pab-miR-396a-5p to Alleviate Psoriasis by Modulating IL-17 Signaling**

**Authors**

*Yali Liu^1,2,3^**^[*](https://ops.straive.com/eProofingSPJ/ExecuteProofHandler.ashx?ExecuteAction=ProofLoad&token=gtVxE16rve0/min2/Xbd5Q&ChapterOrArticleOrBook=Article&Redirect=yes&ProceedToProof=true#corr1)^**^[†](https://ops.straive.com/eProofingSPJ/ExecuteProofHandler.ashx?ExecuteAction=ProofLoad&token=gtVxE16rve0/min2/Xbd5Q&ChapterOrArticleOrBook=Article&Redirect=yes&ProceedToProof=true#afn1)^, Shanmin Tao^1,2^* [*^†^*](https://ops.straive.com/eProofingSPJ/ExecuteProofHandler.ashx?ExecuteAction=ProofLoad&token=gtVxE16rve0/min2/Xbd5Q&ChapterOrArticleOrBook=Article&Redirect=yes&ProceedToProof=true#afn1)*, Zhengwei Zhang**^1,2^, Tianjiao Li^1,2^, Haoran Wang^1,2,3^, Jiankang Mu^1,2^, Yunke Wu^1,2^, Ziheng He^1,2^, Cheng Zhang^2^, Dominique Jasmin Lunter^4^, Peng Cao^1,2,3*^*

**Affiliations**

^1^State Key Laboratory of Technologies for Chinese Medicine Pharmaceutical Process Control and Intelligent Manufacture, Nanjing University of Chinese Medicine, Nanjing 210023, China

^2^Jiangsu Provincial Medical Innovation Center, Affiliated Hospital of Integrated Traditional Chinese and Western Medicine, Nanjing University of Chinese Medicine, Nanjing, 210028, China

^3^Shandong Academy of Chinese Medicine，Jinan 250014, China

^4^Department of Pharmaceutical Technology, Faculty of Science, Eberhard Karls Universität Tübingen, Auf der Morgenstelle 8, 72076, Tuebingen, Germany

^*^Address correspondence to:

✉ [cao_peng@njucm.edu.cn](mailto:cao_peng@njucm.edu.cn) (P.C.); ✉ [yali.liu@njucm.edu.cn](mailto:yali.liu@njucm.edu.cn) (Y.L.)

[†](https://ops.straive.com/eProofingSPJ/ExecuteProofHandler.ashx?ExecuteAction=ProofLoad&token=gtVxE16rve0/min2/Xbd5Q&ChapterOrArticleOrBook=Article&Redirect=yes&ProceedToProof=true#afn1-r) These authors contributed equally to this work.

**Figs. S1 to S31**


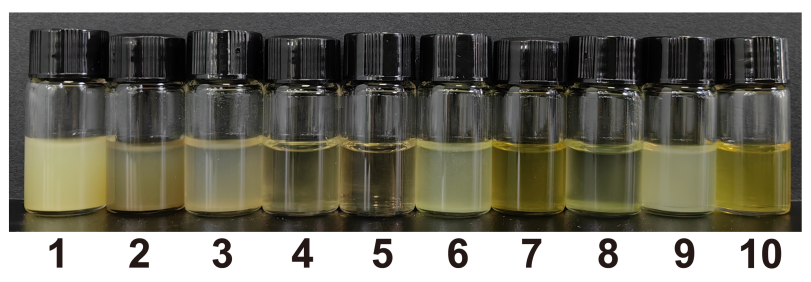


**Figure S1. Photographic demonstration of extracellular vesicle-like particles (EVLPs) derived from various medicinal plants.** Samples of EVLPs were isolated from different plant species following the same preparation process, showing variations in color and opacity. The numbered vials correspond to the following plants: 1: Panax ginseng C. A. Mey; 2: Panax notoginseng F. H. Chen; 3: Angelica sinensis Diels; 4: Mentha haplocalyx Briq; 5: Perilla frutescens Britt; 6: Zingiber officinale Roscoe; 7: Coptis chinensis Franch; 8: Agastache rugosa Kuntze; 9: Astragalus membranaceus Bunge; 10: Curcuma Longa L.


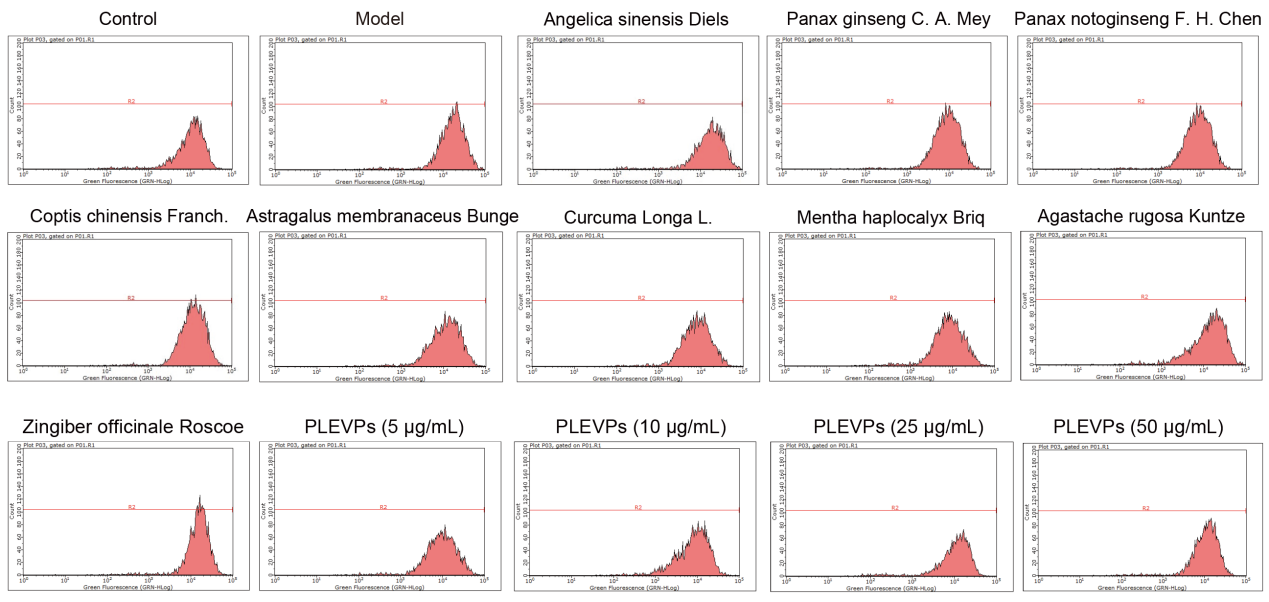


**Figure S2. Flow cytometric analysis of ROS levels in IL-6-induced HaCaT cells treated with various plant-derived EVPs and different concentrations of PLEVPs.** Representative histograms display the fluorescence intensity distribution for each group, including the control group, IL-6 model group, and cells treated with extracellular vesicles from different medicinal plants. Additionally, histograms for cells treated with PLEVPs at concentrations of 5, 10, 25, and 50 µg/mL are presented. The red shaded area indicates the fluorescence intensity corresponding to ROS levels, reflecting the cellular oxidative stress response to each treatment.


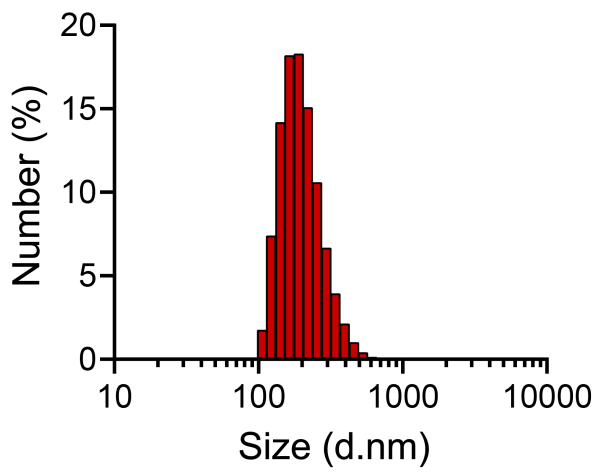


**Figure S3. Size distribution of PLEVPs analyzed by Dynamic Light Scattering (DLS).** The x-axis represents particle diameter (d.nm) on a logarithmic scale, and the y-axis indicates the percentage of particles within each size range. The histogram illustrates a narrow size distribution, highlighting the uniformity of PLEVPs.


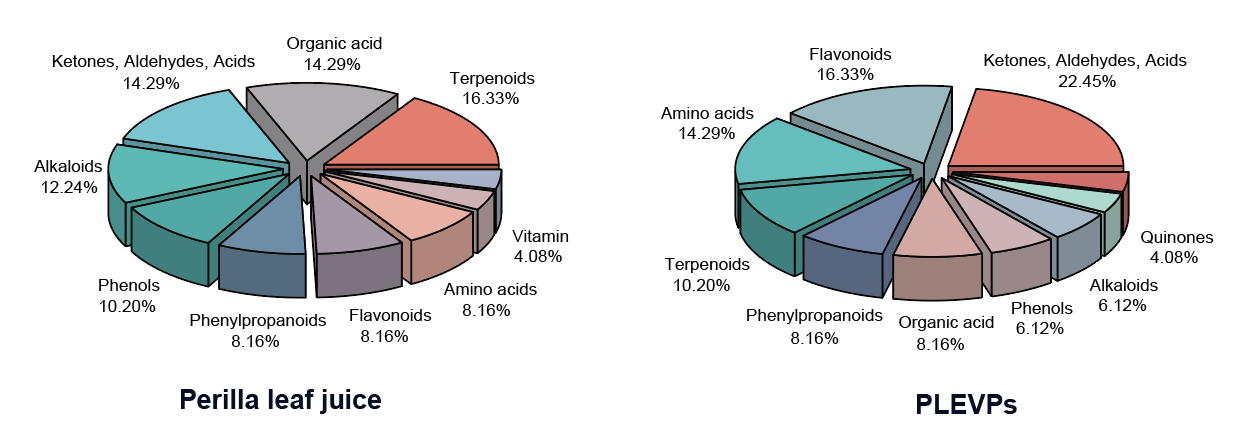


**Figure S4. Comparative metabolomic profiling of Perilla leaf juice and PLEVPs.** Pie charts of relative distribution of key bioactive metabolite classes, including flavonoids, terpenoids, alkaloids, ketones/aldehydes/acids, phenols, amino acids, phenylpropanoids, quinones, organic acids, and vitamins, in Perilla leaf juice and PLEVPs.


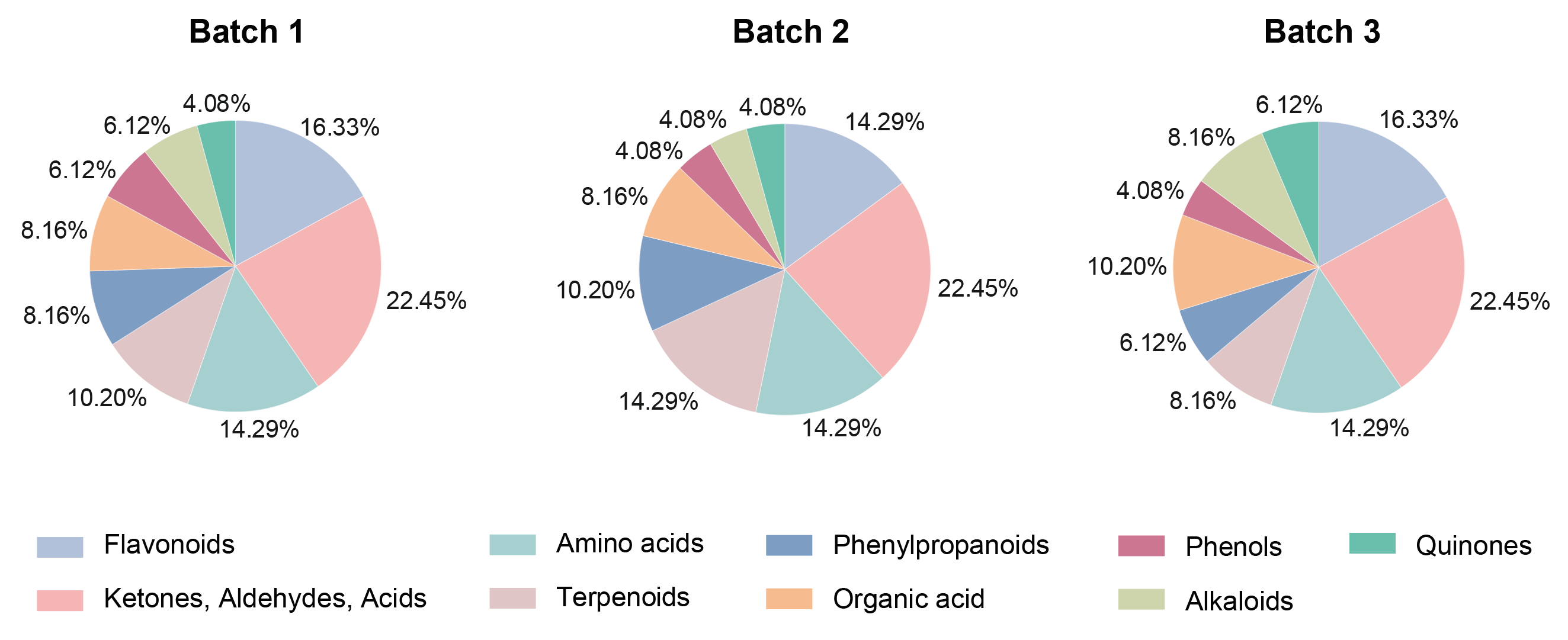


**Figure S5. Metabolomic profiling of PLEVPs from different plant batches.** The pie charts illustrate the relative composition of metabolite categories in three different batches of PLEVPs (Batch 1, Batch 2, and Batch 3). Major metabolite groups include flavonoids, ketones/ aldehydes/ acids, amino acids, terpenoids, phenylpropanoids, organic acids, phenols, alkaloids, and quinones. While minor batch-to-batch variations are observed, demonstrating that the overall metabolic consistency is maintained.


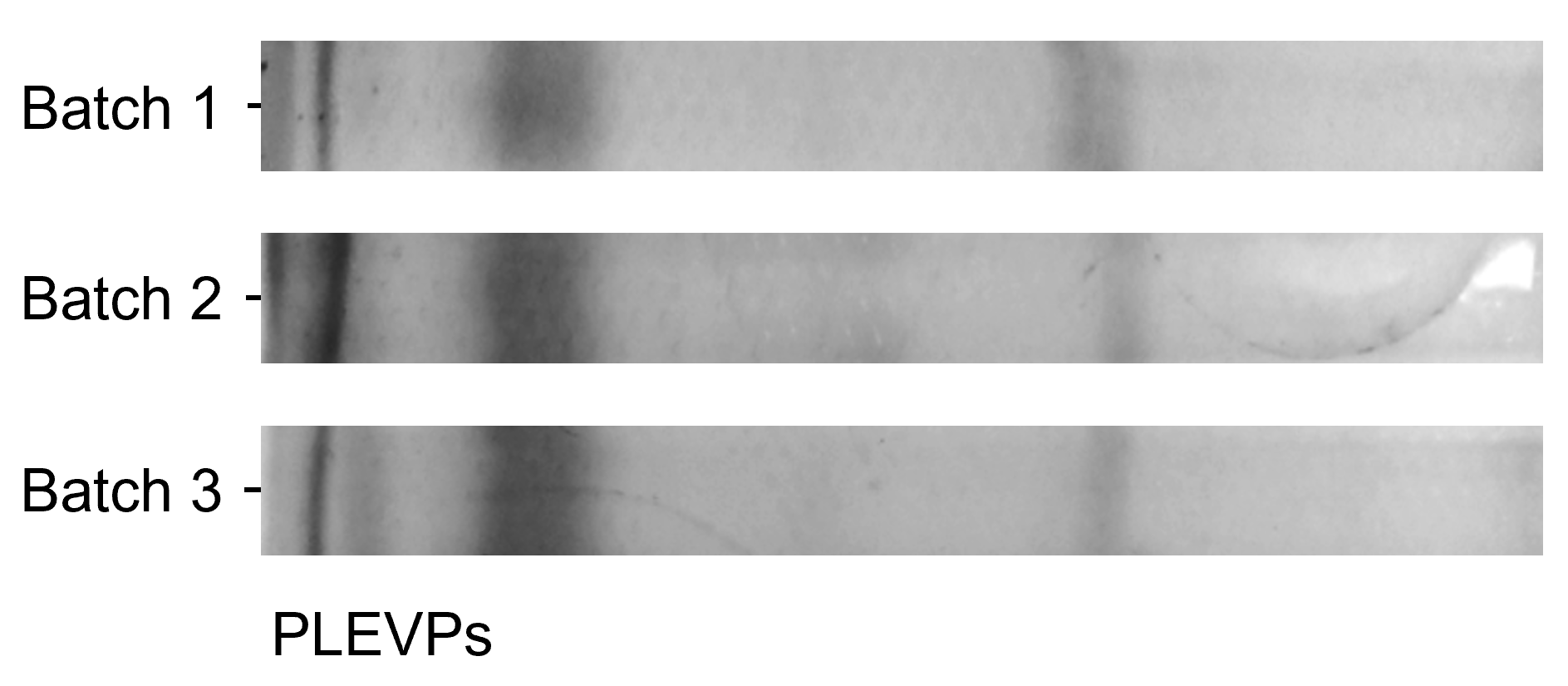


**Figure S6. SDS-PAGE analysis of PLEVPs from different plant batches.** The protein profiles of PLEVPs were analyzed using SDS-PAGE, with samples obtained from three different batches (Batch 1, Batch 2, and Batch 3). The protein bands were observed in each batch, confirming successful isolation of PLEVPs, and no major variations were noted between the batches, supporting relative consistency of PLEVPs.


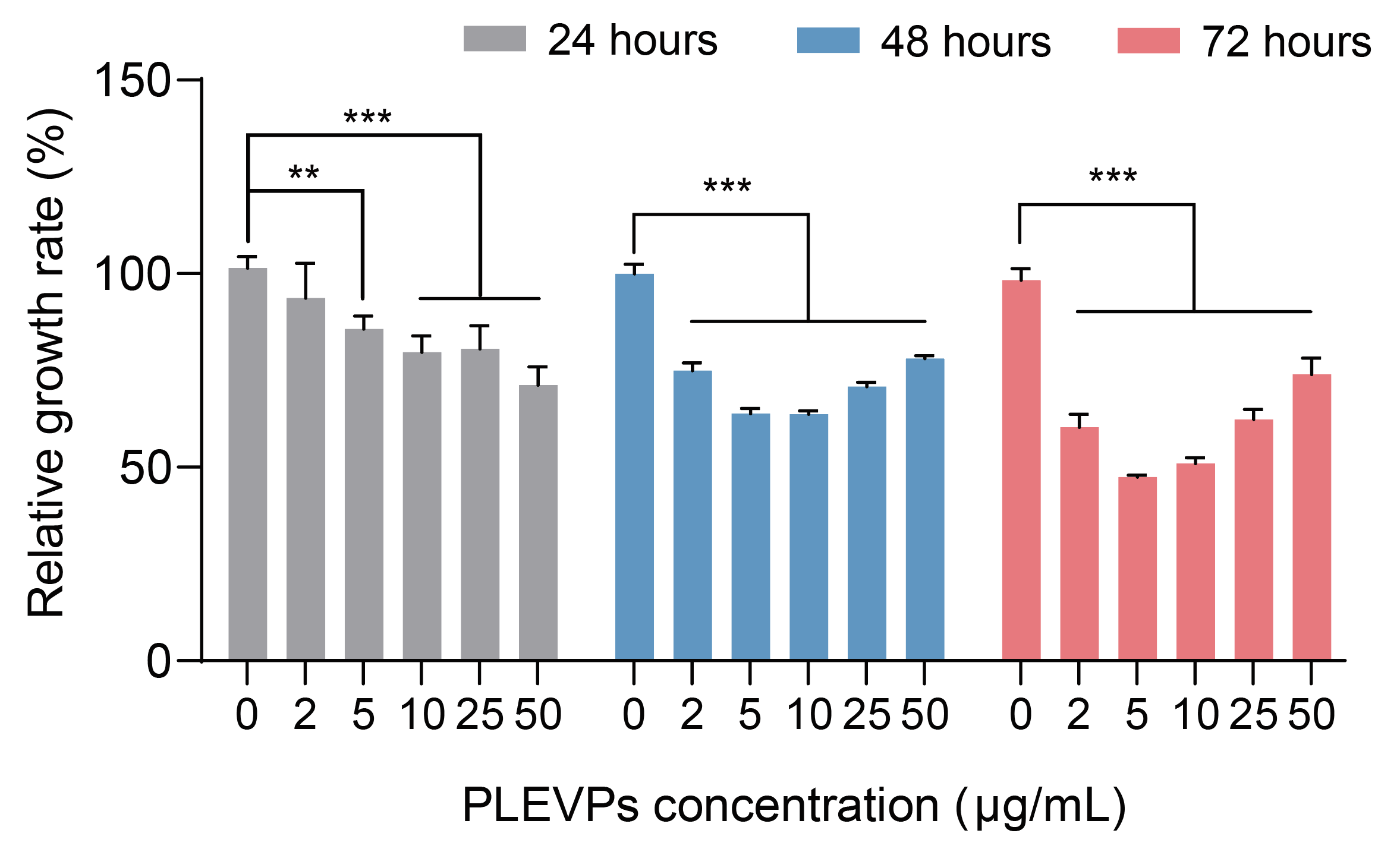


**Figure S7. Effect of PLEVPs on IL-6-stimulated HaCaT cell proliferation at different time points.** HaCaT cells were pre-stimulated with IL-6 and treated with various concentrations of PLEVPs (0, 2, 5, 10, 25, and 50 µg/mL) for 24, 48, and 72 hours. Cell proliferation was measured using CCK-8 assay and expressed as a percentage relative to the untreated control (0 µg/mL). Data are presented as mean ± SD, n = 5. Statistical analysis was performed using one-way ANOVA, with significance levels indicated as *P < 0.05, **P < 0.01, and ***P < 0.001.


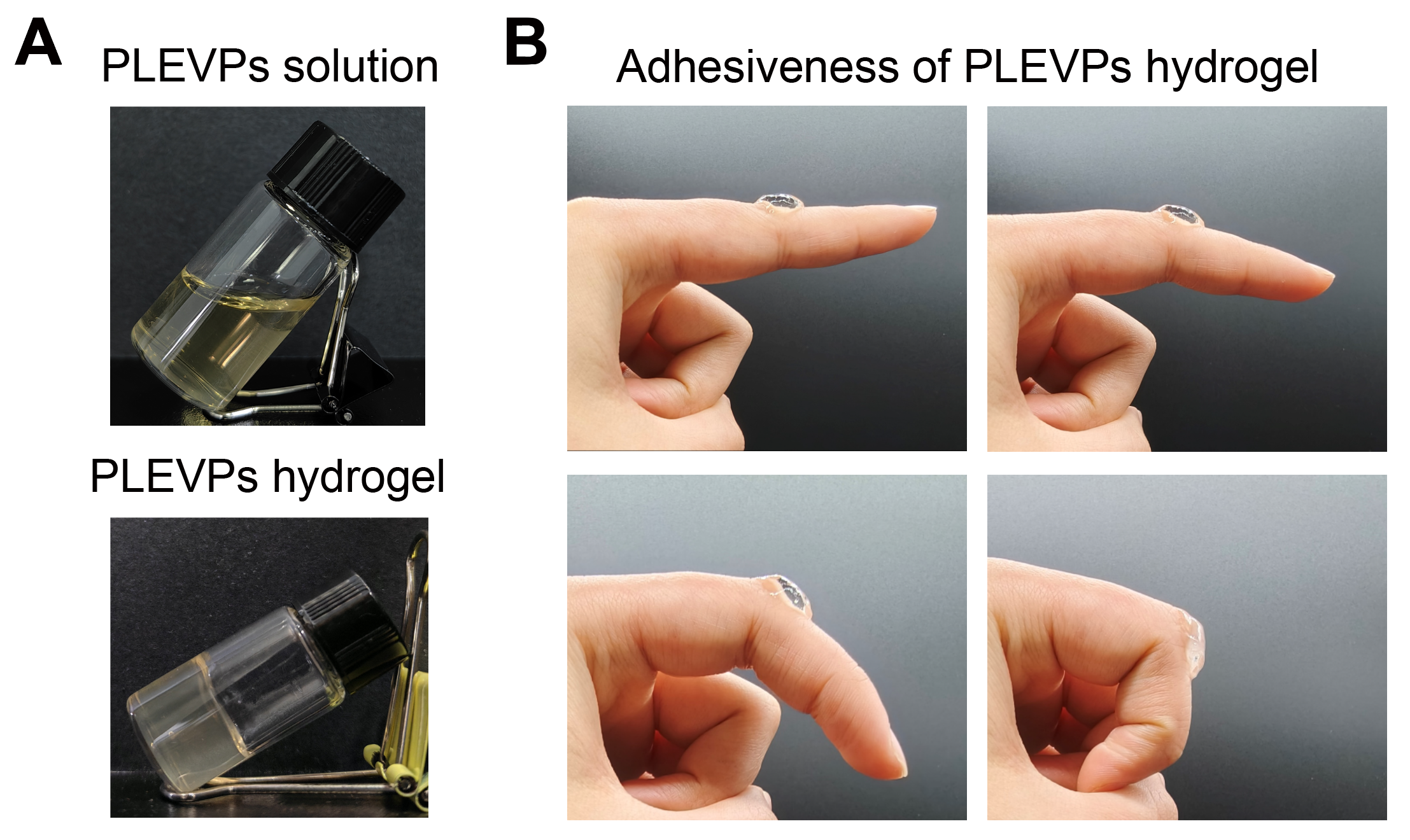


**Figure S8. Comparison of PLEVPs solution and PLEVPs hydrogel.** (A) Visual comparison of the PLEVPs solution (top) and PLEVPs hydrogel (bottom) showing the change in consistency after hydrogel preparation. (B) Adhesiveness of PLEVPs hydrogel, demonstrating its ability to adhere to the skin under different finger positions. The hydrogel maintains strong adhesion even when the finger is bent or straight.


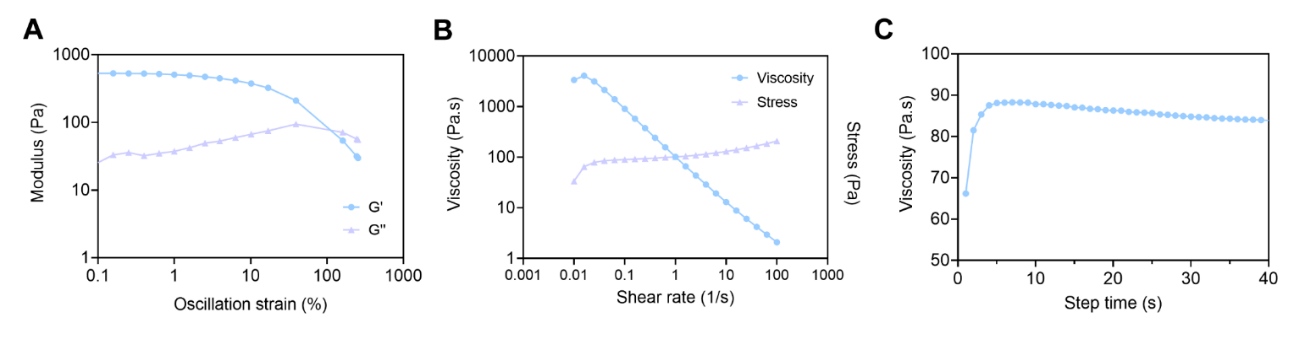


**Figure S9. Rheological characterization of PLEVPs-loaded Carbopol hydrogel.** (A) Oscillation strain sweep shows the storage modulus (G') and loss modulus (G") of the PLEVPs hydrogel at different strain values. (B) Shear rate sweep indicating viscosity and stress behavior as a function of shear rate. (C) Step time viscosity profile showing the stability of hydrogel over time under steady shear conditions.


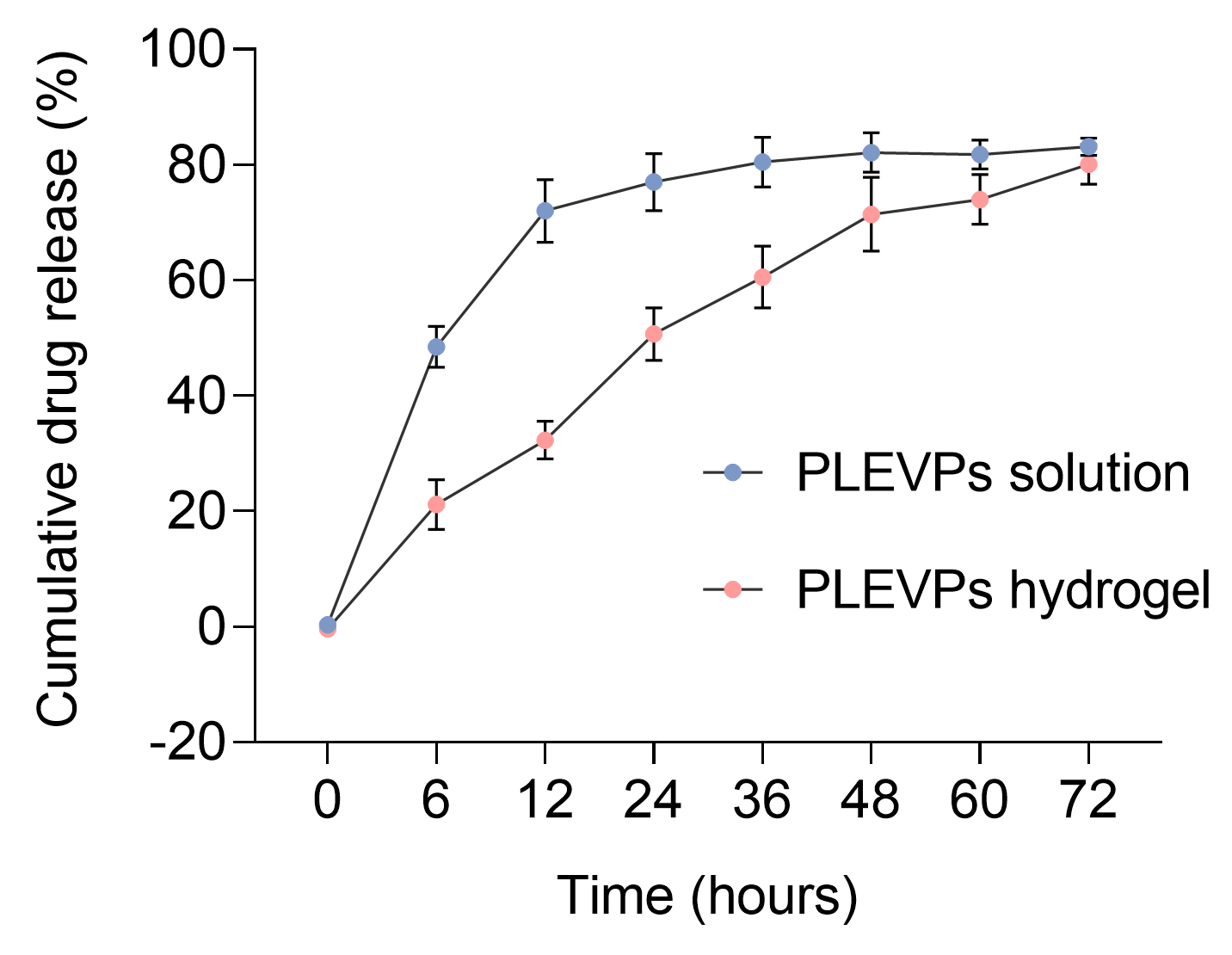


**Figure S10. Cumulative drug release profile of PLEVPs.** The release kinetics of PLEVPs solution and PLEVPs hydrogel were evaluated over a 72-hour period. PLEVPs solution exhibited rapid release, reaching approximately 80% within the first 24 hours, while PLEVPs hydrogel demonstrated sustained release with a gradual increase over time. Data are presented as mean ± SD (n = 3).


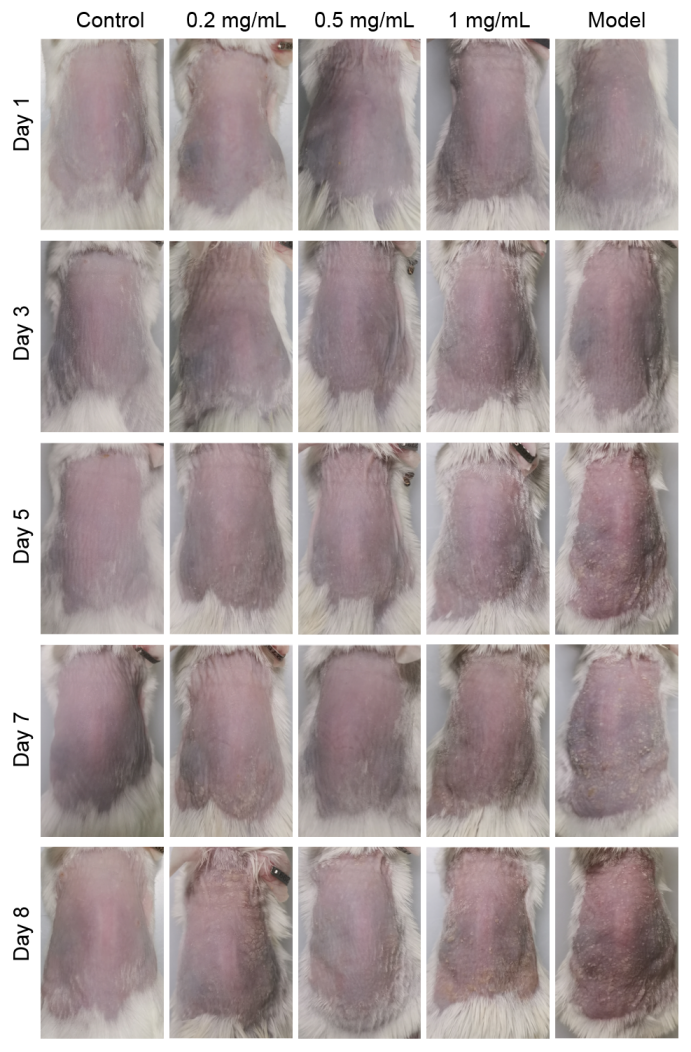


**Figure S11. Visual assessment of back skin in psoriasis prevention studies with topical application of PLEVPs hydrogel.** Representative images of mouse back skin lesions recorded on days 1, 3, 5, 7, and 8 across different treatment groups: control group, model group, and PLEVPs treatment groups with varying concentrations (0.2 mg/mL, 0.5 mg/mL, and 1 mg/mL). Mice treated with PLEVPs hydrogel showed progressive improvement in skin condition over time. In contrast, the model group displayed continuous skin deterioration, indicating significant psoriasis-like symptoms.


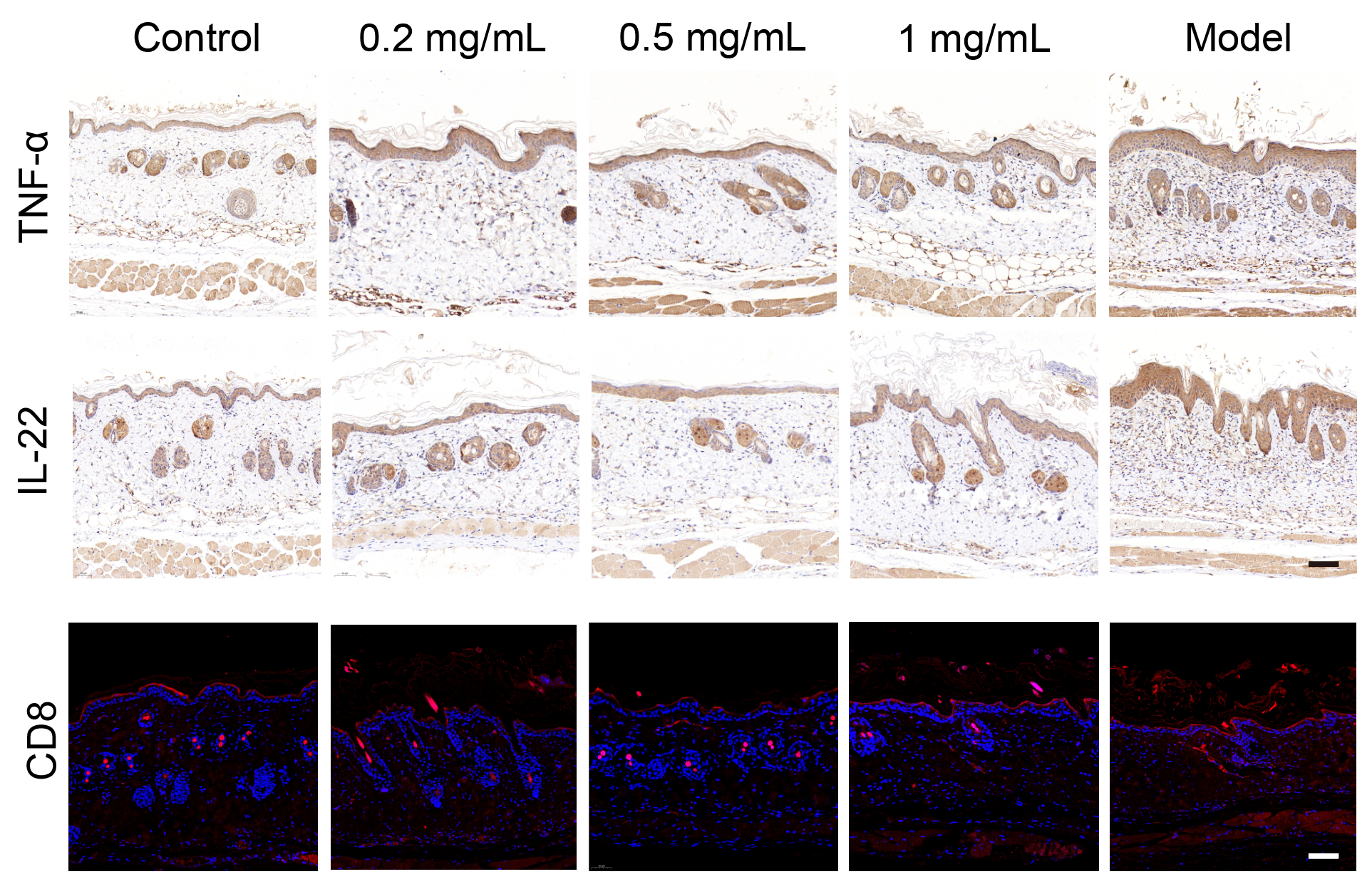


**Figure S12. Immunohistochemical (IHC) and immunofluorescence (IF) analyses of TNF-α, IL-22, and CD8 in mouse skin tissue from in vivo prevention studies.** Skin sections from different treatment groups (Control, 0.2 mg/mL, 0.5 mg/mL, 1 mg/mL PLEVPs hydrogel, and Model) were analyzed. The top two rows show IHC staining for TNF-α and IL-22, respectively. The bottom row displays IF staining for CD8+ cells, with red indicating CD8 expression and blue representing nuclear staining. Scale bar, 100 μm. Compared to the Model group, the PLEVPs treatment groups exhibit reduced expression of inflammatory markers and decreased immune cell infiltration.


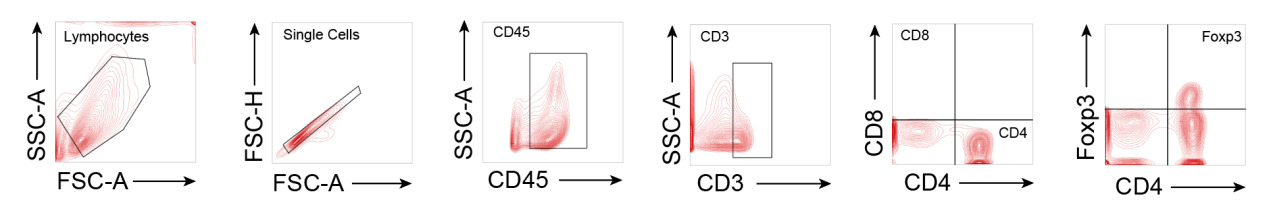


**Figure S13. Gating strategy for identifying CD45⁺ CD4⁺ Foxp3⁺ regulatory T cells.** Lymphocyte populations were initially gated based on the FSC-A vs. SSC-A scatter plot. Single cells were subsequently selected using the FSC-H vs. FSC-A plot to exclude doublets. CD45⁺ cells were then gated to identify leukocytes, followed by CD3⁺ gating to identify T cells. Within the T cell population, CD4⁺ and CD8⁺ T cell subsets were distinguished using the CD4 vs. CD8 plot. Finally, regulatory T cells (Tregs) were identified within the CD4⁺ population by Foxp3 expression.


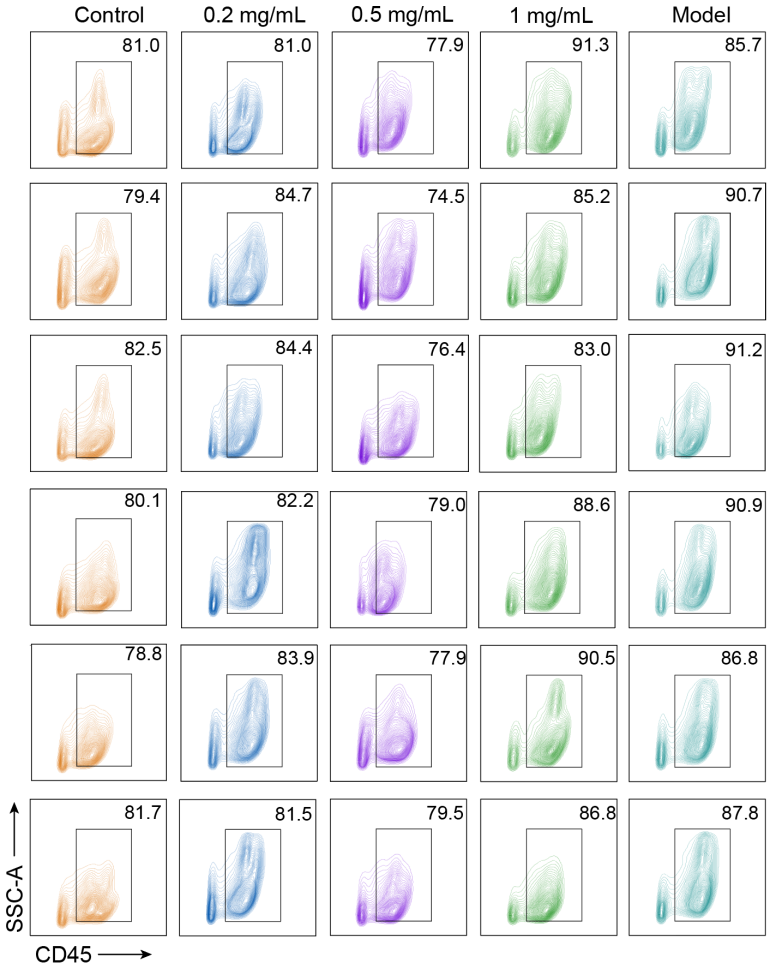


**Figure S14.** **Flow cytometric analysis of CD45⁺ cells in mouse skin single-cell suspensions from prevention studies.** Flow cytometry was used to assess the percentage of CD45⁺ cells in skin samples across different treatment groups: Control, 0.2 mg/mL, 0.5 mg/mL, 1 mg/mL PLEVPs hydrogel, and Model. The values in each panel indicate the proportion of CD45-positive cells.


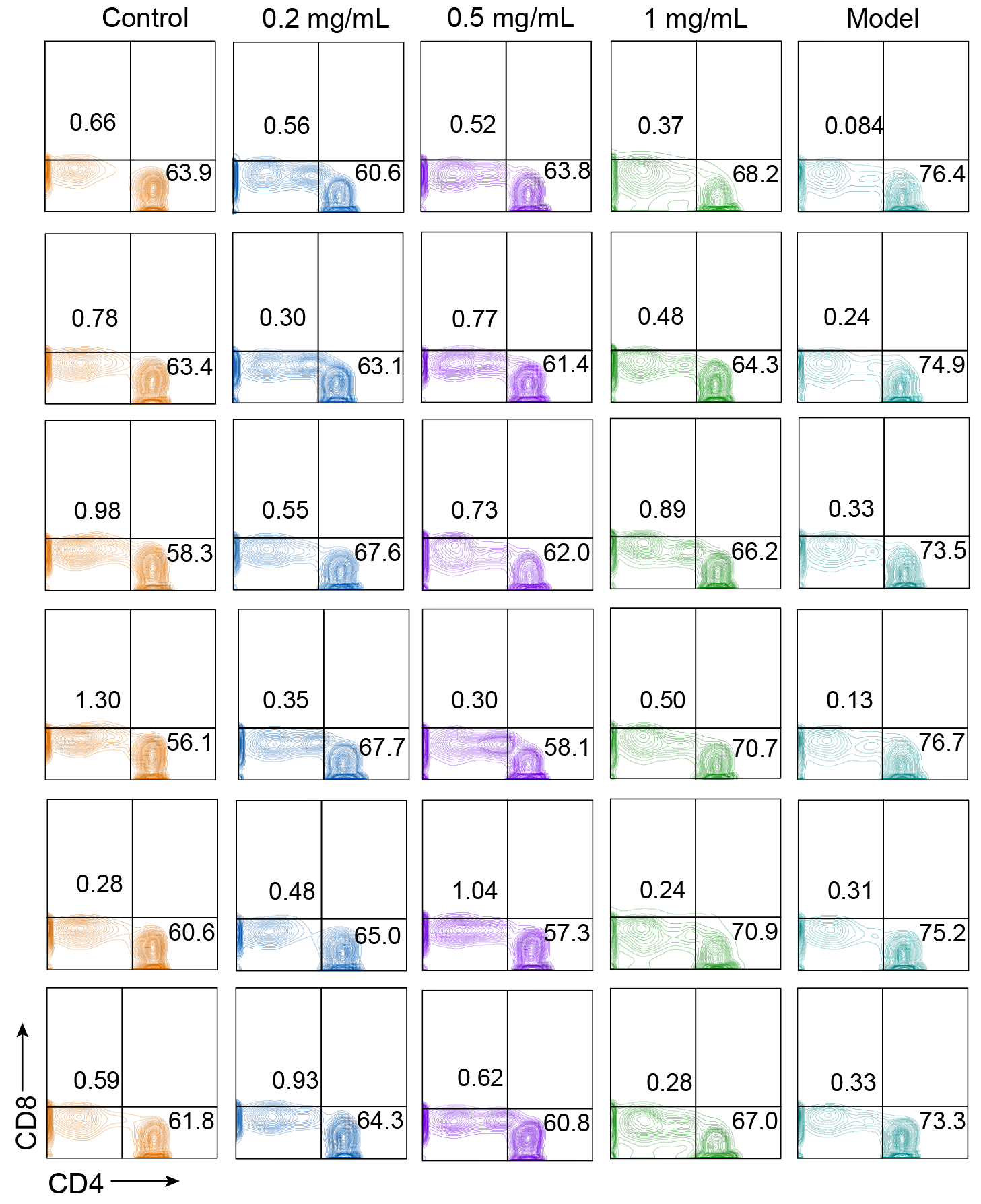


**Figure S15.** **Flow cytometric analysis of CD4⁺ and CD8⁺ T cell populations in mouse skin single-cell suspensions from prevention studies.** Flow cytometry was used to determine the proportions of CD4⁺ and CD8⁺ T cells across different treatment groups: Control, 0.2 mg/mL, 0.5 mg/mL, 1 mg/mL PLEVPs hydrogel, and Model. The values in each panel represent the percentage of CD4⁺ and CD8⁺ T cell subsets within the gated populations.


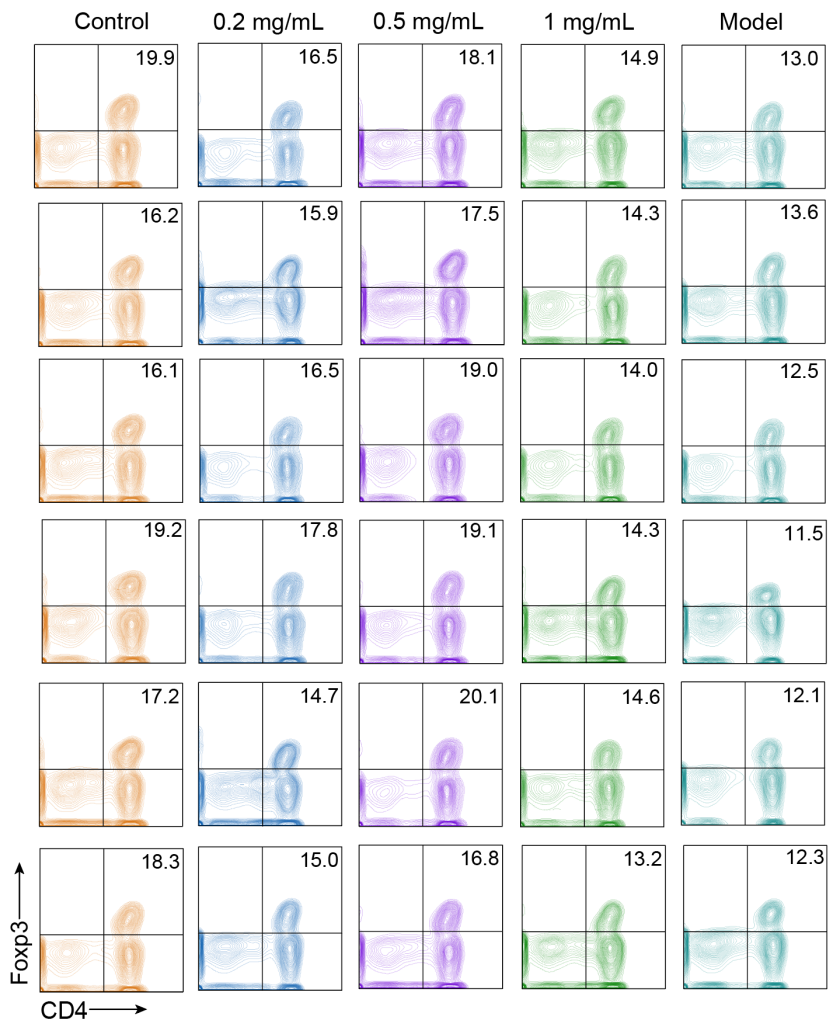


**Figure S16.** **Flow cytometric analysis of CD4⁺ Foxp3⁺ regulatory T cells (Tregs) in mouse skin single-cell suspensions from prevention studies.** Flow cytometry was performed to quantify the proportions of CD4⁺ Foxp3⁺ Tregs across different treatment groups: Control, 0.2 mg/mL, 0.5 mg/mL, 1 mg/mL PLEVPs hydrogel, and Model. The values in each panel represent the percentage of CD4⁺ Foxp3⁺ T cells within the gated populations.


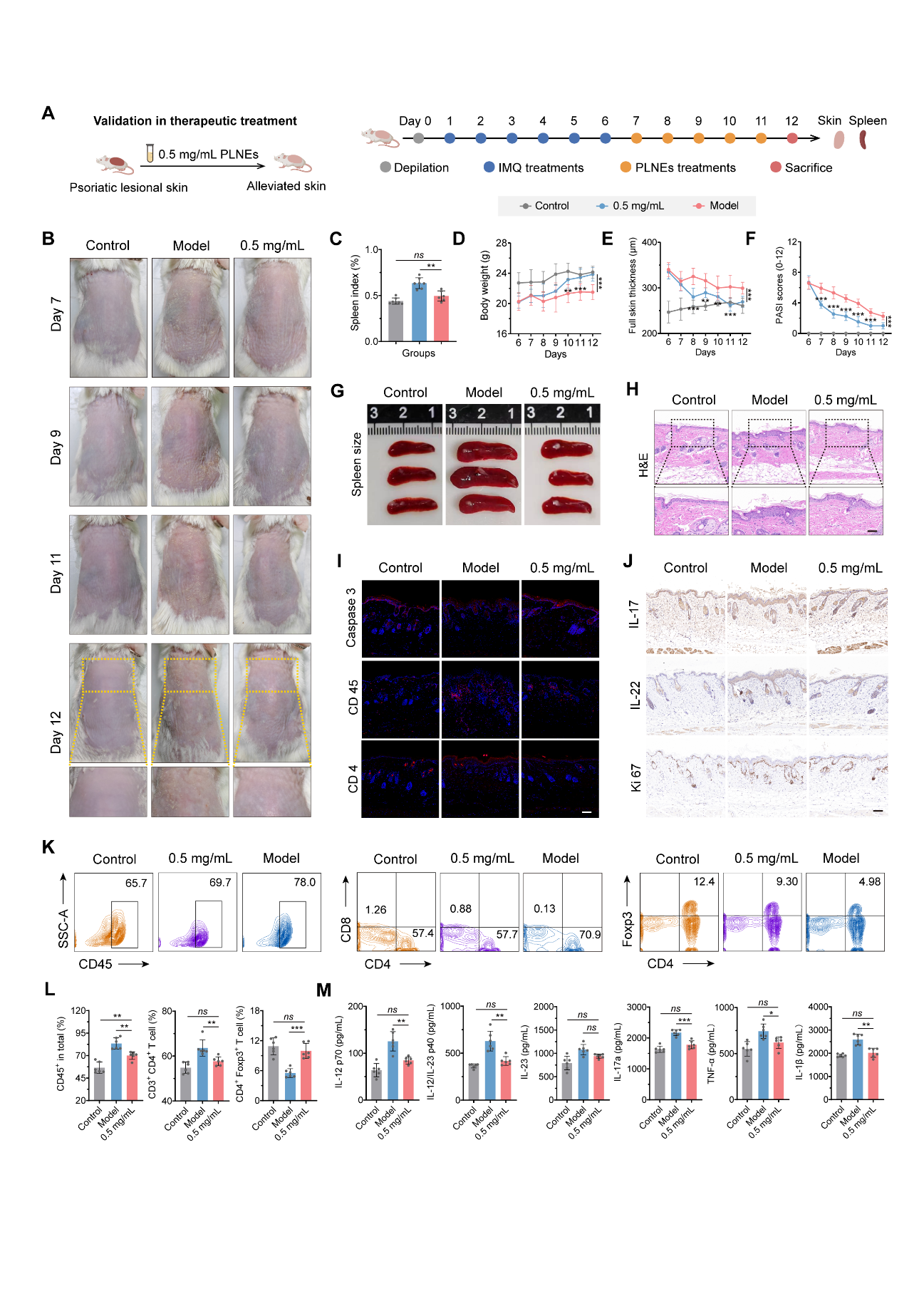


**Figure S17.** **Therapeutic effects of PLEVPs hydrogel in a psoriasis post-induction treatment model.** (A) Experimental timeline illustrating the induction of psoriasis-like symptoms with IMQ treatment, followed by therapeutic administration of PLEVPs hydrogel after model establishment. (B) Representative images of dorsal skin condition on days 7, 9, 11, and 12 across treatment groups: Control, Model, and 0.5 mg/mL PLEVPs hydrogel. (C) Spleen index comparison among groups. (D) Body weight, (E) skin thickness, and (F) PASI scores throughout the post-induction treatment course. (G) Hematoxylin and eosin (H&E) staining of skin sections with observation of epidermal thickness and inflammatory conditions. Scale bar, 100 μm. (H) Comparison of spleen size among different groups. (I) IF staining for Caspase-3, CD45, and CD4 in skin sections, indicating apoptosis and immune cell infiltration. Scale bar, 100 μm. (J) IHC for IL-17, IL-22, and Ki67, assessing inflammatory response and cell proliferation in skin tissue. Scale bar, 100 μm. (K) Flow cytometry analysis of spleen single-cell suspensions, quantifying CD8⁺ T cells, CD4⁺ T cells, and CD4⁺ Foxp3⁺ Tregs. (L) Quantification of immune cell populations in spleen tissue. (M) Measurement of inflammatory cytokines (IL-12p70, IL-12/23p40, IL-23p19, IL-17a, TNF-α, IL-1β) levels in serum using ELISA kits. Data are presented as mean ± SD, n = 6. Statistical analysis was performed using one-way ANOVA with *P < 0.05, **P < 0.01, and ***P < 0.001.


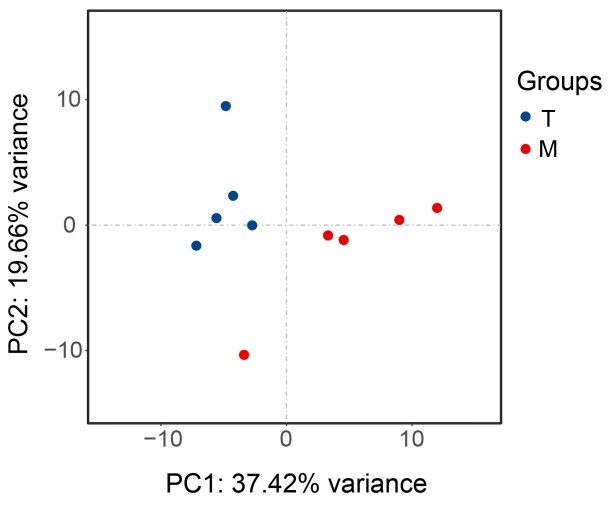


**Figure S18. Principal Component Analysis (PCA) of transcriptional profiles in the topical treatment of PLEVPs hydrogel and model groups.** The scatter plot shows the distribution of samples from the T (PLEVPs) group (blue) and the M (Model) group (red). Principal Component 1 (PC1) and Principal Component 2 (PC2) account for 37.42% and 19.66% of the variance, respectively. The distinct clustering of the two groups reflects differences in their transcriptional profiles.


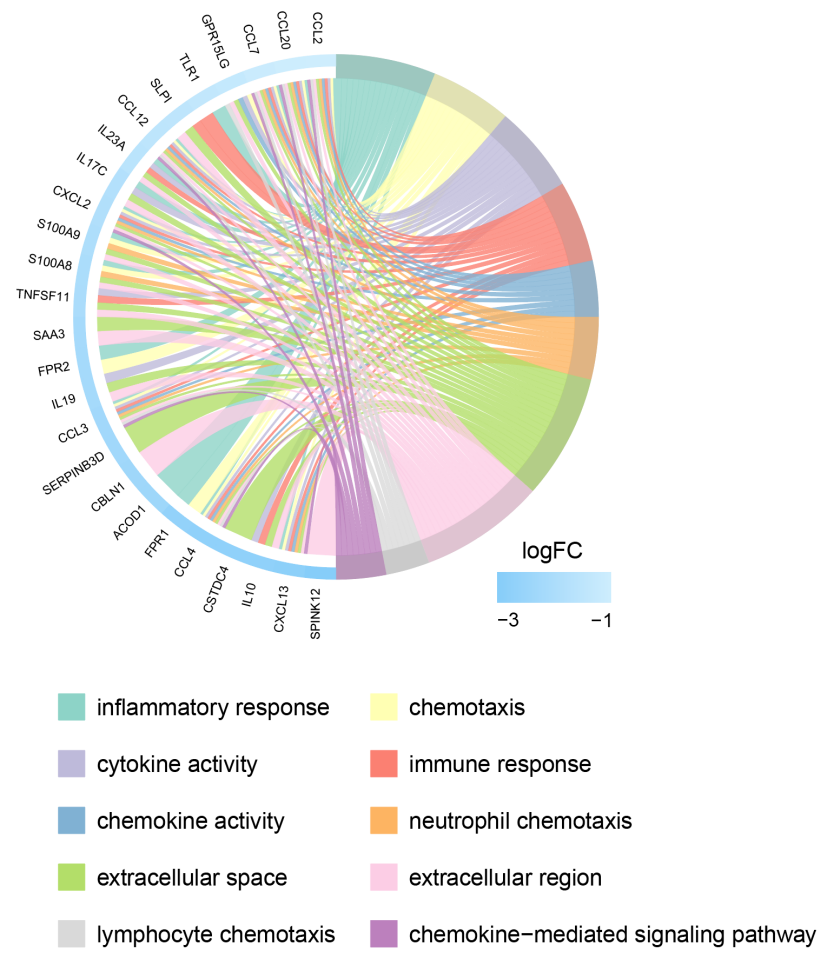


**Figure S19. Gene Ontology (GO) analysis of transcriptional changes in response to the treatment of PLEVPs hydrogel.** The chord diagram illustrates changes in gene expression associated with various biological processes, with each color representing a specific GO term, such as inflammatory response (green), chemotaxis (yellow), and immune response (red). The connecting lines indicate associations between genes and functional categories, while color intensity reflects logFC values, representing the magnitude of gene expression changes.


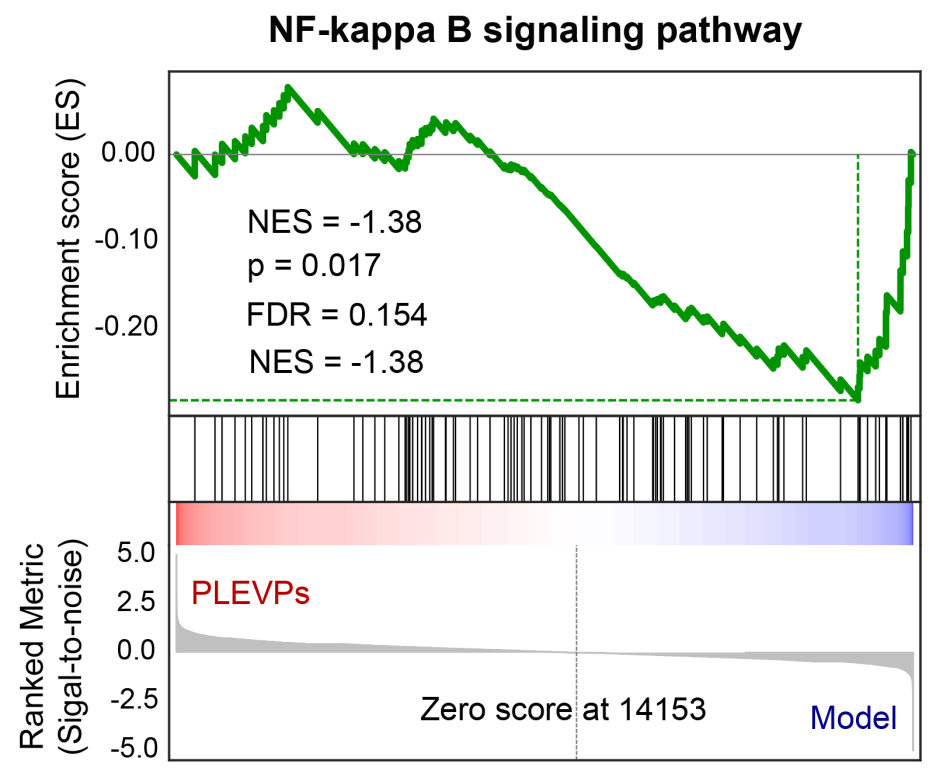


**Figure S20. Gene Set Enrichment Analysis (GSEA) of NF-kappaB signaling pathway in transcriptional studies.** The GSEA plot illustrates significant enrichment of the NF-kappaB signaling pathway in the model group (red) compared to the PLEVPs hydrogel-treated group (blue). The negative enrichment score (NES = -1.38) suggests downregulation of this pathway in the PLEVPs group. Statistical analysis shows a p-value of 0.017 and a false discovery rate (FDR) of 0.154.


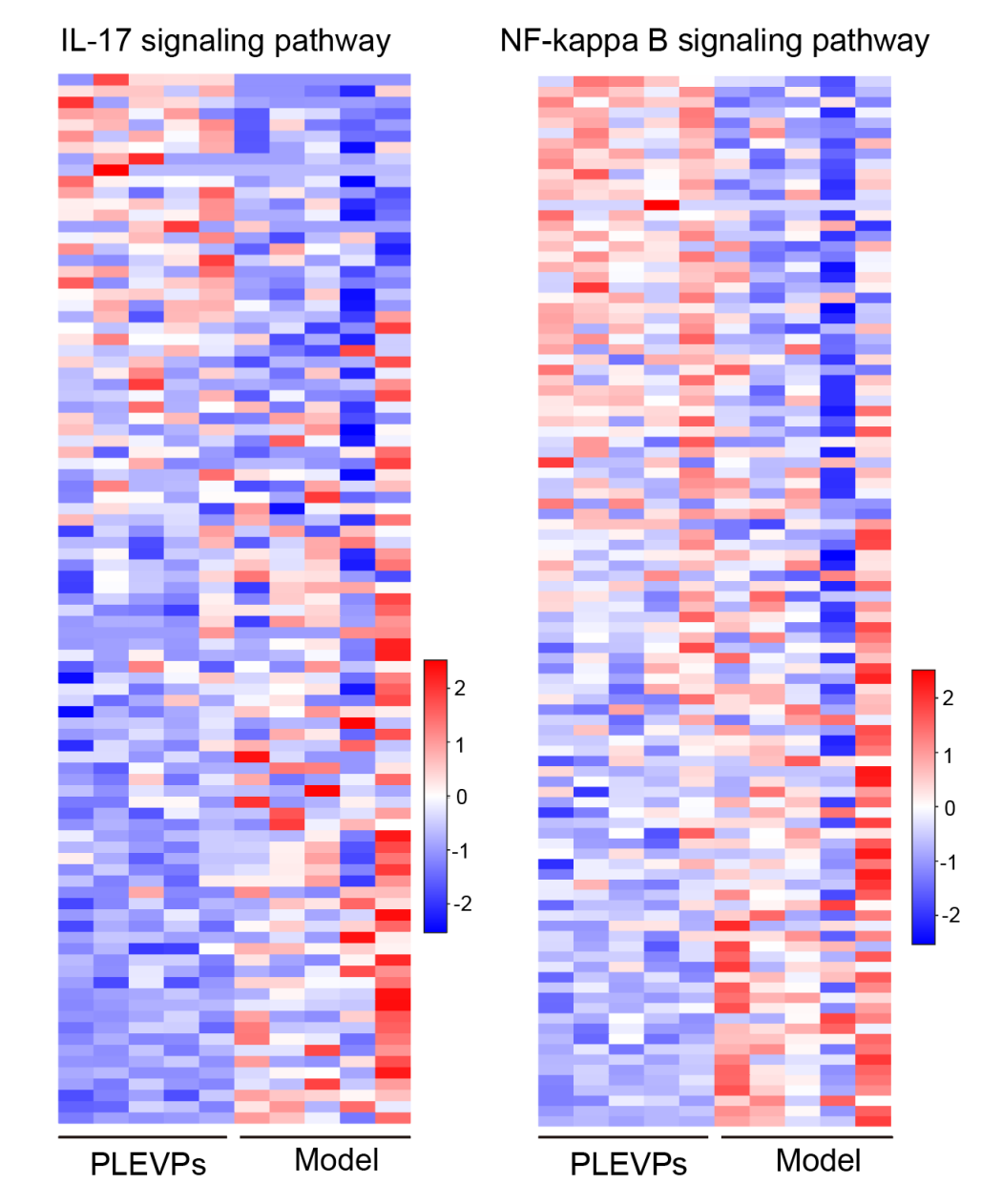


**Figure S21. Heatmap analysis of IL-17 and NF-kappa B signaling pathways in transcription studies.** The left panel represents the IL-17 signaling pathway, and the right panel represents the NF-kappa B signaling pathway. Color intensity indicates the level of gene expression, with red denoting upregulation and blue denoting downregulation, as shown by the color scale.


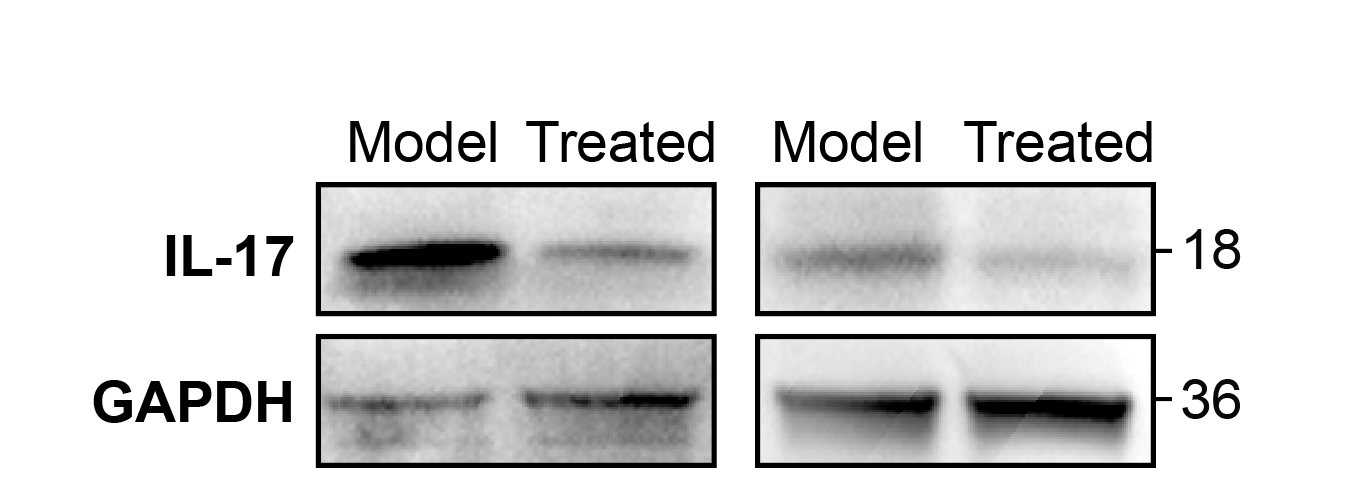


**Figure S22. Western blot analysis of IL-17 protein levels.** The upper panels show IL-17 protein expression in the Model and PLEVPs hydrogel-treated groups, with GAPDH used as a loading control in the lower panels. Molecular weight markers (in kDa) are indicated on the right (IL-17: 18 kDa; GAPDH: 36 kDa).


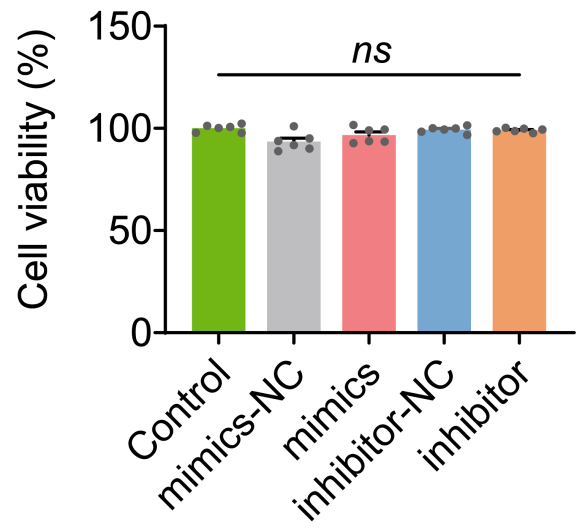


**Figure S23.** **Cell viability 24 hours after transfection.** Cell viability was measured in five groups: Control, mimics-NC, mimics, inhibitor-NC, and inhibitor. Results are presented as percentages relative to the control group, with no significant differences (ns) observed among groups, indicating that transfection with mimics or inhibitors did not affect cell viability under these experimental conditions. Data are shown as mean ± SD, n = 6, and were analyzed using one-way ANOVA.


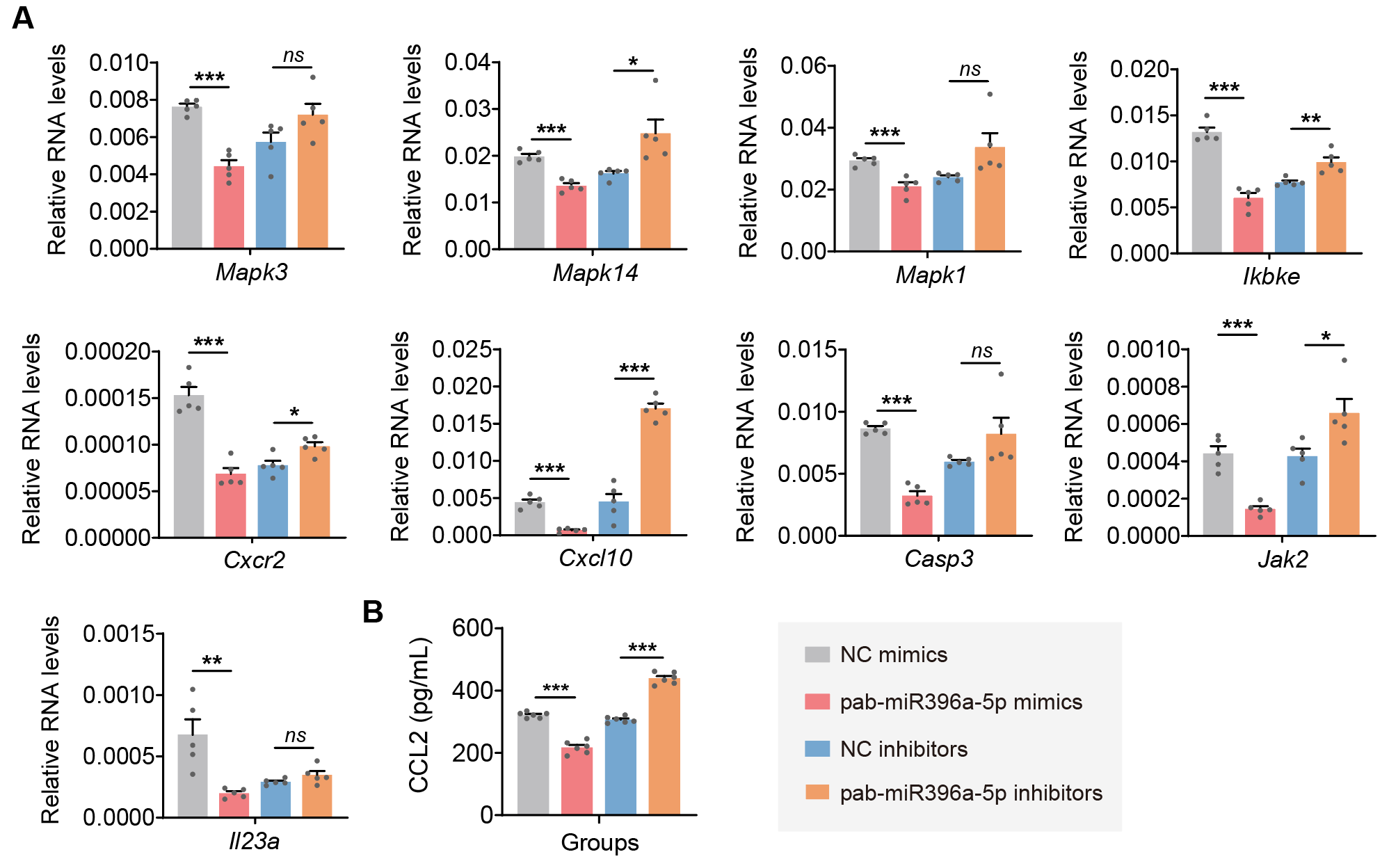


**Figure S24.** **mRNA and cytokine levels following cell transfection.** (A) Relative mRNA levels of *mapk3*, *mapk14*, *mapk1*, *ikbke*, *cxcr2*, *cxcl10*, *casp3*, *jak2*, and *il23a* were measured in cells treated with NC mimics, miR396a-5p mimics, NC inhibitors, and miR396a-5p inhibitors. Significant differences in gene expression were observed among the groups. (B) CCL2 cytokine levels were quantified using an ELISA kit in each treatment group, showing modulation by miR396a-5p mimics and inhibitors. Data are shown as mean ± SD, n = 5, ns indicates no significant difference. Statistical significance was determined by one-way ANOVA, with *P < 0.05, **P < 0.01, and ***P < 0.001.


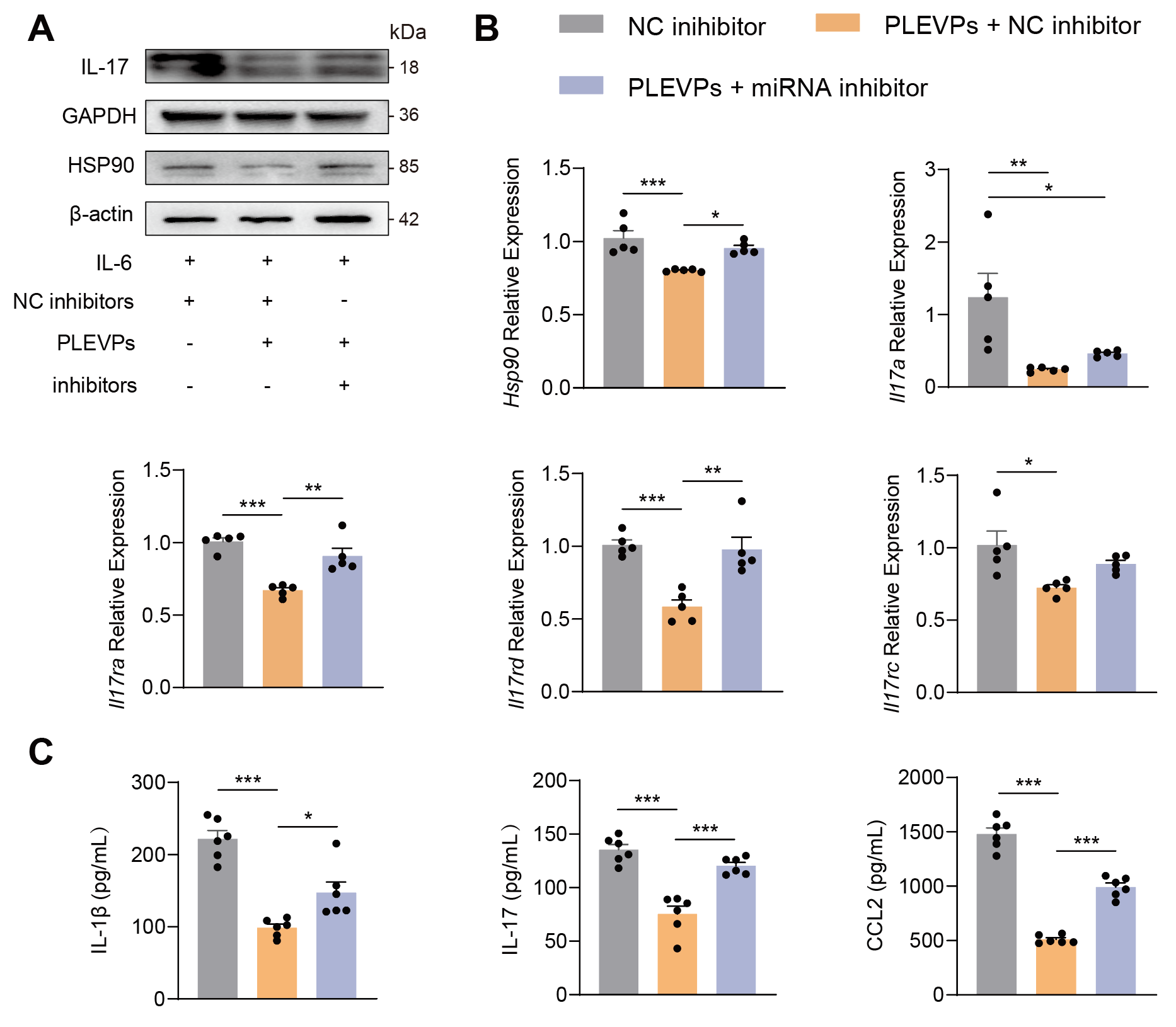


**Figure S25. Impact of PLEVPs and miRNA inhibitors on IL-17 signaling pathway in IL-6-stimulated HaCaT cells.** (A) Western blot analysis showing protein levels of IL-17, HSP90 in cells all treated with IL-6, and grouped with NC inhibitor, PLEVPs + NC inhibitor, and PLEVPs + miRNA inhibitors. (B) Relative mRNA expression of *Hsp90, Il17a Il17ra, Il17rd, and Il17rc,* measured by PCR in the same treatment groups. Data are presented as mean ± SD, n = 5. (C) ELISA quantification of IL-1β, IL-17, and CCL2 in cell culture supernatants. Statistical analysis was performed using one-way ANOVA, with *P < 0.05, **P < 0.01, ***P < 0.001 indicating statistical significance.


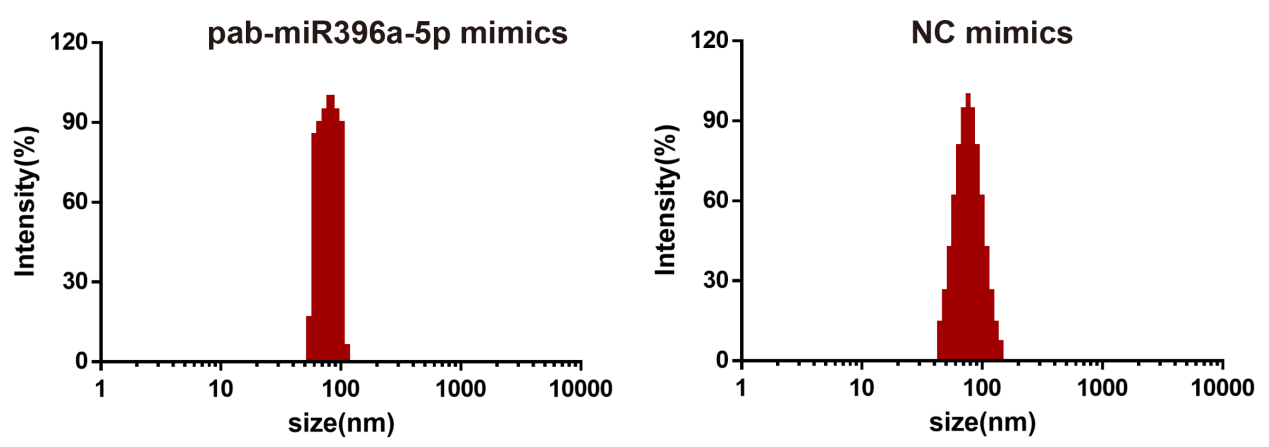


**Figure S26.** **Particle size distribution of lipid nanoparticles (LNPs) encapsulating pab-miR396a-5p mimics and NC mimics.** Dynamic light scattering (DLS) analysis was conducted to assess the particle size distribution of LNPs encapsulating pab-miR396a-5p mimics (left) and NC mimics (right). Both samples show a monodisperse distribution, with peak intensity around 80 nm, indicating a uniform particle size.


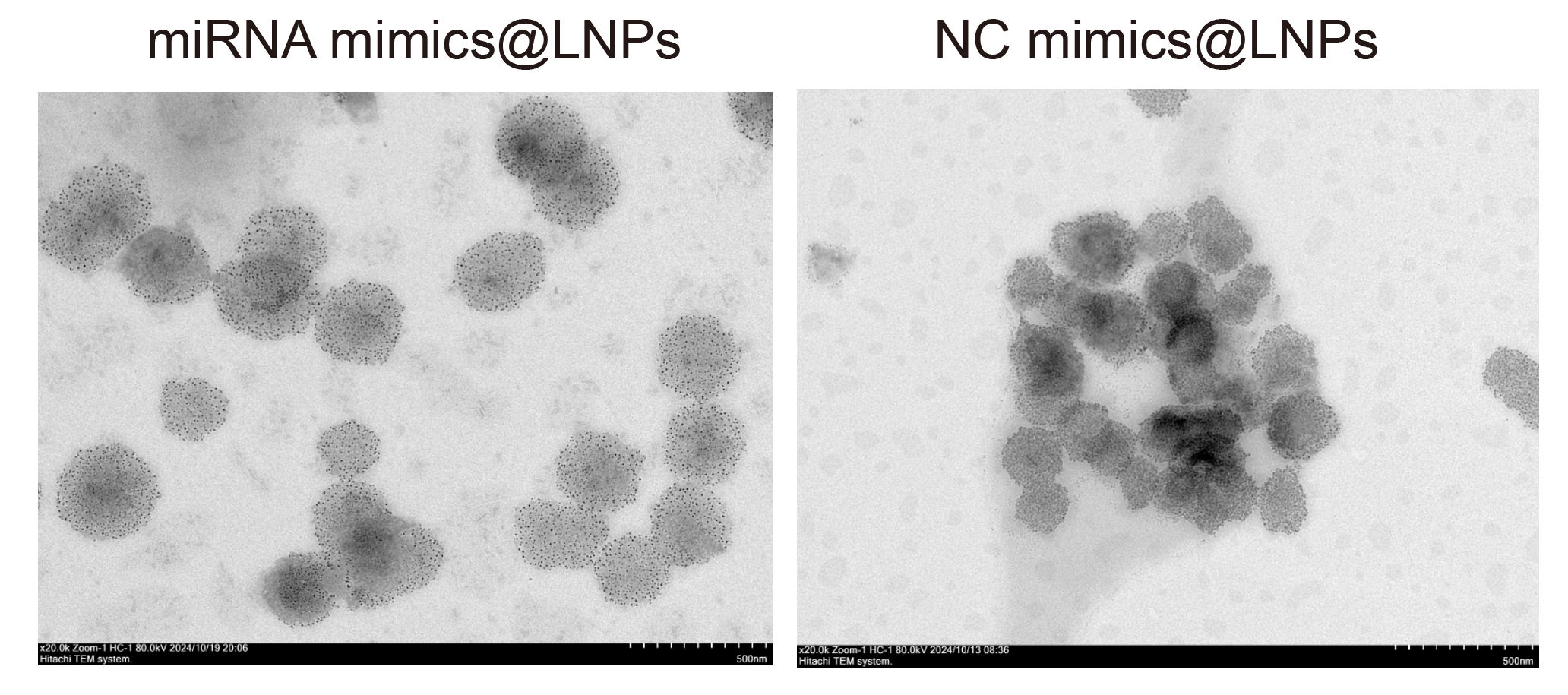


**Figure S27.** **Transmission electron microscopy (TEM) images of LNPs encapsulating miRNA mimics and NC mimics.** TEM images display the structural morphology of miRNA mimics@LNPs (left) and NC mimics@LNPs (right). Both types of LNPs exhibit spherical shapes with consistent particle size. Scale bars: 500 nm.


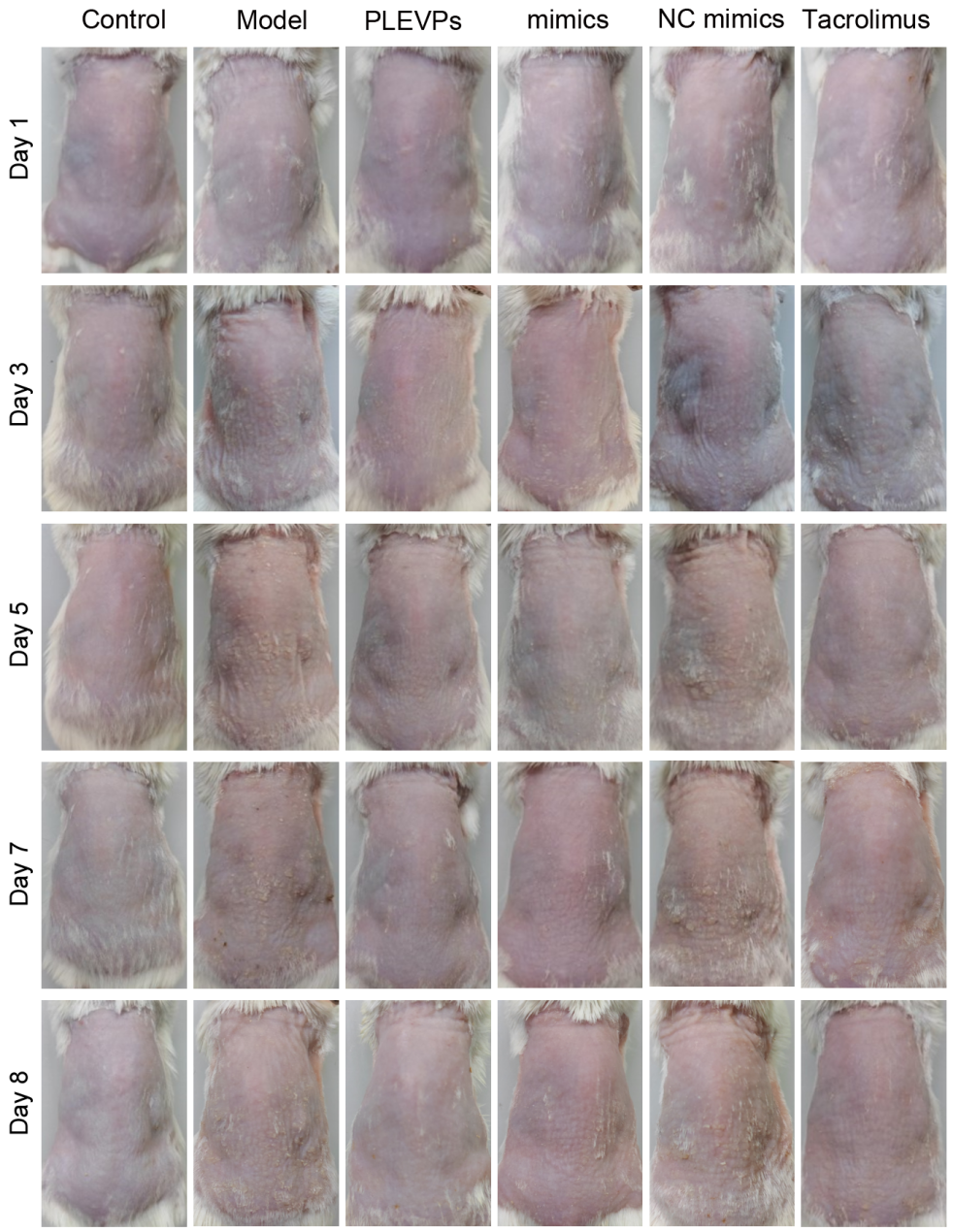


**Figure S28. Back skin condition during combined modeling and treatment across different groups.** Skin conditions of the mouse model were recorded on days 1, 3, 5, 7, and 8 across six groups: Control, Model, PLEVPs, mimics, NC mimics, and Tacrolimus. Photographs illustrate the progression of skin appearance in each group under simultaneous modeling and treatment conditions.


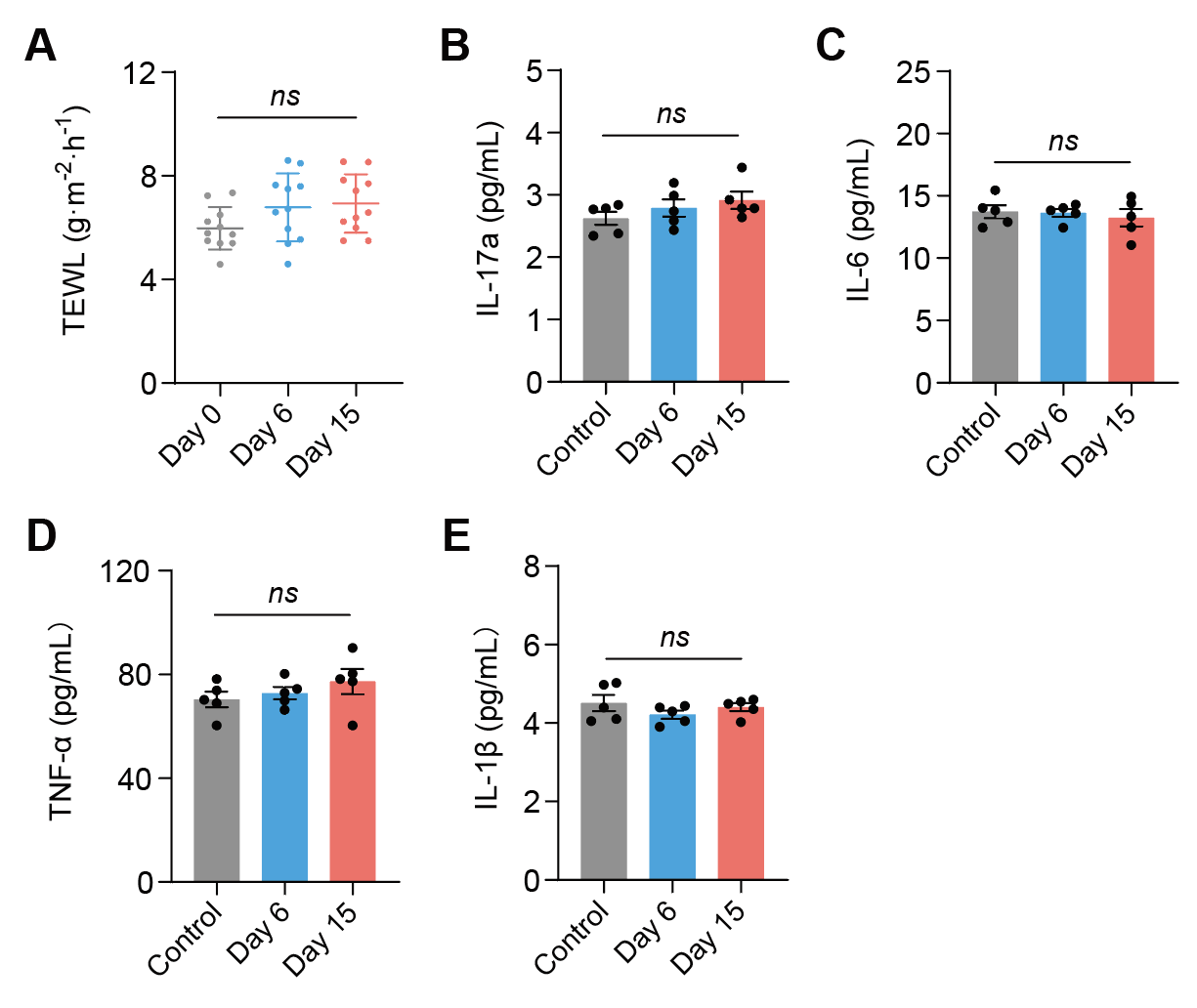


**Figure S29. Effects of PLEVPs hydrogel on skin barrier integrity and inflammatory cytokine levels after repeated administration.** (A) Trans-epidermal water loss (TEWL) measurements at Day 0, Day 6, and Day 15 after repeated PLEVPs hydrogel administration (n = 12). (B–E) ELISA quantification of inflammatory cytokines of skin samples (n = 6), including IL-17A (B), IL-6 (C), TNF-α (D), and IL-1β (E). Data are presented as mean ± SD. Statistical analysis was performed using one-way ANOVA. *ns* denotes no significance.


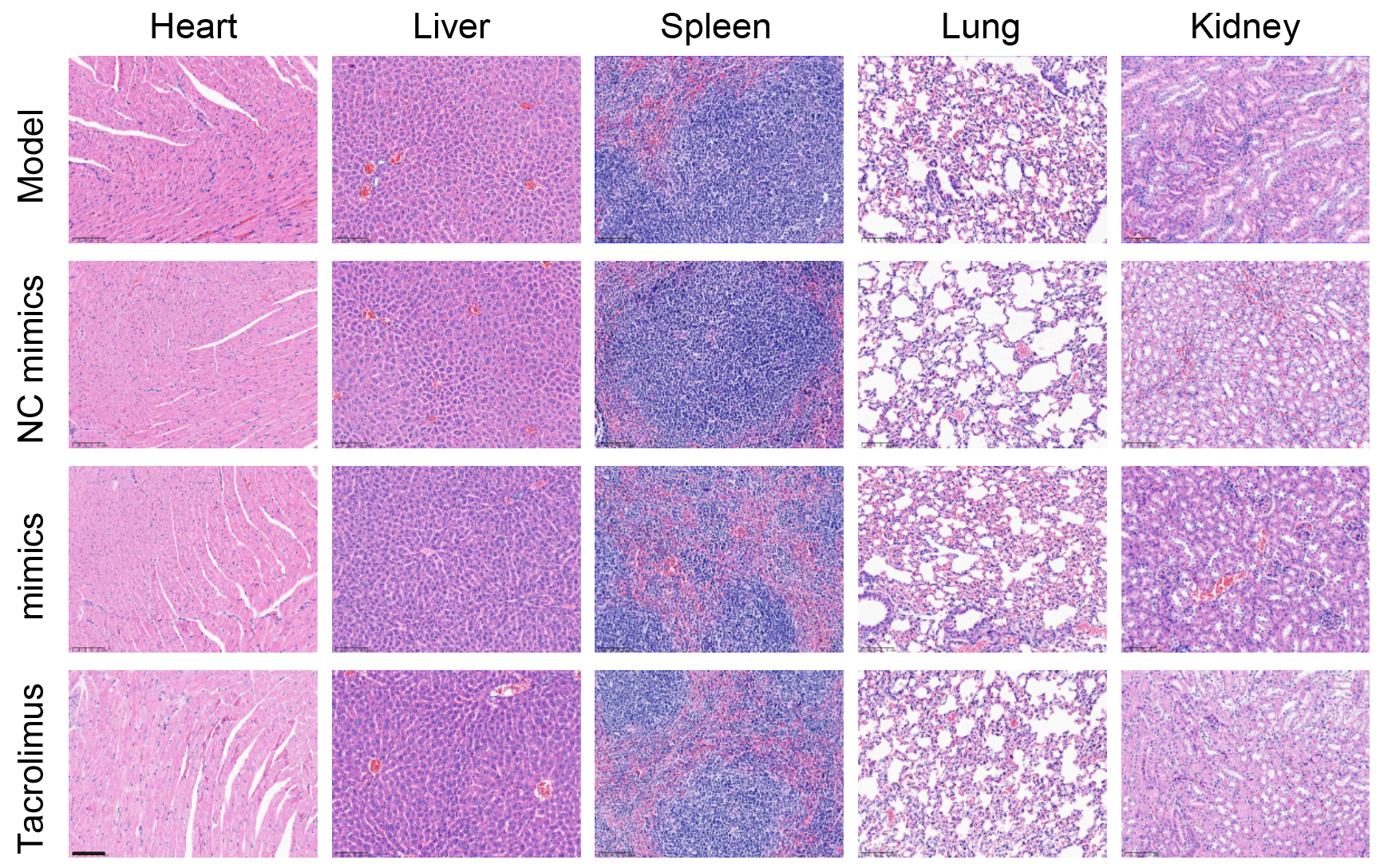


**Figure S30.** **Histological analysis of main organs following treatment.** H&E staining of major organs (heart, liver, spleen, lungs, kidneys) from five groups of Control, PLEVPs, mimics, NC mimics, and Tacrolimus on Day 8 to evaluate potential histopathologic changes after treatments. Scale bar, 100 μm.


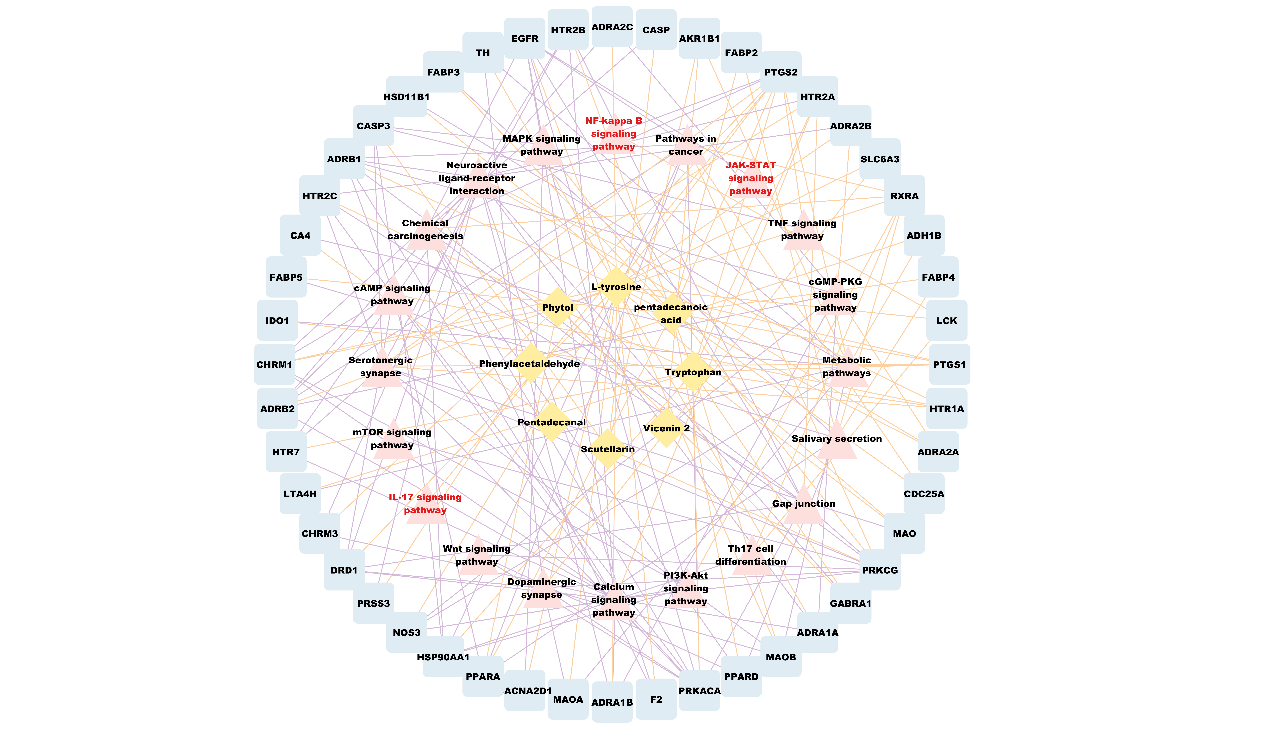


**Figure S31.** **Interaction network of active components from PLEVPs, signaling pathways, and target genes.** This circular network diagram illustrates the predicted interactions among active components in PLEVPs, relevant signaling pathways, and target genes. The innermost layer represents bioactive compounds identified in PLEVPs via metabolomics analysis. The middle layer displays signaling pathways modulated by these compounds, with notable emphasis on the NF-kB, JAK-STAT, and IL-17 pathways, which are key regulatory axes in psoriasis pathology. The outer layer consists of target genes involved in these pathways. Connections between layers highlight the interactions from PLEVPs components through specific pathways to genetic targets, suggesting a potential modulatory role of PLEVPs in these critical psoriasis-related pathways.

**Tables S1 to S3**

**Table S1. Characteristics of LNPs encapsulating pab-miR396a-5p mimics and NC mimics.** Summary of particle size, polydispersity index (PDI), and encapsulation efficiency for LNPs loaded with pab-miR396a-5p mimics and NC mimics.

| Name | Pab-miR396a-5p mimics | NC mimics |
| --- | --- | --- |
| Particle Size | 82.65 nm | 79.74 nm |
| PDI | 0.172 | 0.102 |
| Encapsulate Efficiency | 98.37% | 98.62% |

**Table S2. miRNA-mRNA interaction prediction.** Based on the scoring system using bioinformatics tools, genes with higher total scores and lower energy values are considered more likely to be directly targeted by pab-miR396a-5p. Multiple instances of predicted interactions with HSP83A and HSC80 were further validated experimentally.

| miRNA | Total Score | Total Energy | Max Score | Max Energy | Transcript Length | Symbol |
| --- | --- | --- | --- | --- | --- | --- |
| pab-miR396a-5p | 2298 | -287 | 176 | -30.4 | 1332 | HSP83A |
| pab-miR396a-5p | 2190 | -277.22 | 176 | -30.4 | 1338 | HSP83A |
| pab-miR396a-5p | 2082 | -242.49 | 176 | -30.4 | 2130 | HSC80 |
| pab-miR396a-5p | 2030 | -229.97 | 144 | -24.6 | 2100 | HSC80 |
| pab-miR396a-5p | 1639 | -186.04 | 144 | -23.41 | 2127 | HSC80 |
| pab-miR396a-5p | 1173 | -138.97 | 145 | -22.02 | 1332 | HSP83A |
| pab-miR396a-5p | 102 | -13.85 | 102 | -13.85 | 1338 | HSC80 |

**Table S3. Sequence information of pab-miR396a-5p.** Nucleotide sequence (5’ to 3’) of pab-miR396a-5p used in the study.

| Name | Sequence (5’ to 3’) |
| --- | --- |
| pab-miR396a-5p | TTCCACAGCTTTCTTGAACT |
